# Global disease burden linked to diet high in red meat and colorectal cancer from 1990 to 2019 and its prediction up to 2030

**DOI:** 10.3389/fnut.2024.1366553

**Published:** 2024-03-14

**Authors:** Xuesong Yang, Duozhi Wu, Yanbo Liu, Zhigang He, Anne Manyande, Hongjun Fu, Hongbing Xiang

**Affiliations:** ^1^Department of Anesthesiology and Pain Medicine, Hubei Key Laboratory of Geriatric Anesthesia and Perioperative Brain Health, Wuhan Clinical Research Center for Geriatric Anesthesia, Tongji Hospital, Tongji Medical College, Huazhong University of Science and Technology, Wuhan, China; ^2^Department of Anesthesiology, Hainan General Hospital, Haikou, Hainan, China; ^3^School of Human and Social Sciences, University of West London, London, United Kingdom; ^4^Department of Orthopedics, Xiangyang Hospital of Traditional Chinese Medicine, Xiangyang, Hubei, China; ^5^Key Laboratory of Anesthesiology and Resuscitation (Huazhong University of Science and Technology), Ministry of Education, Wuhan, China

**Keywords:** colorectal cancer, red meat, global burden of disease, mortality, disability-adjusted life years, health inequality, epidemiology

## Abstract

**Background:**

Numerous studies have already identified an association between excessive consumption of red meat and colorectal cancer (CRC). However, there has been a lack of detailed understanding regarding the disease burden linked to diet high in red meat and CRC.

**Objective:**

We aim to offer evidence-based guidance for developing effective strategies that can mitigate the elevated CRC burden in certain countries.

**Methods:**

We used the data from the Global Burden of Disease (GBD) Study 2019 to evaluate global, regional, and national mortality rates and disability-adjusted Life years (DALYs) related to diet high in red meat. We also considered factors such as sex, age, the socio-demographic index (SDI), and evaluated the cross-national inequalities. Furthermore, we utilized DALYs data from 204 countries and regions to measure cross-country inequalities of CRC by calculating the slope index of inequality and concentration index as standard indicators of absolute and relative inequalities.

**Discussion:**

The results show that globally, the age-standardized mortality rate (ASMR) and age-standardized disability adjusted life year rate (ASDR) related to CRC due to diet high in red meat have decreased, with estimated annual percent change (EAPCs) of −0.32% (95% CI −0.37 to −0.28) and-0.18% (95% CI −0.25 to −0.11). Notably, the burden was higher among males and the elderly. The slope index of inequality rose from 22.0 (95% CI 18.1 to 25.9) in 1990 to 32.9 (95% CI 28.3 to 37.5) in 2019 and the concentration index fell from 59.5 (95% CI 46.4 to 72.6) in 1990 to 48.9 (95% CI 34.6 to 63.1) in 2019. Also, according to our projections, global ASDR and ASMR might tend to increase up to 2030.

**Conclusion:**

ASMR and ASDR for CRC associated with high red meat diets declined globally from 1990 to 2019, but the absolute number of cases is still rising, with men and the elderly being more affected. CRC associated with diets high in red meat exhibits significant income inequality, placing a disproportionate burden on wealthier countries. Moreover, according to our projections, ASMR and ASDR are likely to increase globally by 2030. In order to address this intractable disease problem, understanding changes in global and regional epidemiologic trends is critical for policy makers and others.

## 1 Introduction

Colorectal cancer (CRC), also known as colorectal adenocarcinoma, primarily arises from the colon’s glands and epithelial cells (1). Worldwide, CRC is ranked third in cancer prevalence among males and second among females (2). It is worth noting that more than half of the cases occur in more developed and industrialized countries (3, 4). In these countries, CRC is the second most common tumor, with a lifetime incidence of 5% (5–7). In regions with varying levels of economic development, there are significant differences in the 5-year survival rate of CRC. For instance, the overall 5-year survival rate in the United States is over 60%, whereas in developing countries, it is less than 40% (5).

The International Agency for Research on Cancer (IARC) defines red meat as unprocessed mammalian muscle meat (8, 9). Dietary red meat, including beef, veal, pork, lamb, and mutton, provides us with several important essential nutrients (10, 11). These essential nutrients comprise protein, essential amino acids, vitamins (including vitamin B12), minerals (including heme iron and zinc), and other micronutrients (12). With the recent economic growth, the global demand for red meat has surged, in both developed and developing countries (13). However, abundant evidence indicates that the diet high in red meat is associated with a range of health issues (14–16), including two major chronic diseases: cardiovascular disease and CRC (17–19). A 1975 survey revealed a robust correlation (*r* = 0.9) between *per capita* meat consumption and the incidence of CRC among women from 23 different countries (20). Other research also reported that an increase in red meat consumption by 100 grams per day is associated with an 11–51% greater risk of various cancer incidence and appears to be unrelated to any health benefits (21). IARC has classified red meat consumption as a probable human carcinogen based on evidence related to CRC, pancreatic cancer, and prostate cancer (Group 2A) (9). Recent guidelines recommend that the public should moderate their daily intake of red meat (22).

Previous Global Burden of Disease (GBD) studies predominantly emphasized the disease burden from high dietary red meat, rather than specifically addressing the CRC burden attributable to such consumption (23, 24). Currently, no detailed report exists based on the GBD dataset focusing on the CRC burden from high global red meat consumption. Furthermore, there is a lack of research that predicts the CRC disease burden based on GBD study results. Hence, we utilized data from GBD 2019 to determine the temporal trends in incidence and disability-adjusted life years (DALYs) at the global, regional, and national levels, stratified by sex, age, and SDI. We systematically summarized the global burden and health development status, including the unequal distribution of disease burden among countries and the forecast for the CRC disease burden up to 2030, in order to provide evidence for policymakers. Given the public accessibility of the data, ethical approval and informed consent were not required for our study.

## 2 Materials and methods

### 2.1 Data source

The data from the Global Burden of Disease (GBD) for the year 2019 were used to estimate the incidence and burden of CRC. Data on CRC were sourced from various outlets, including hospital records, emergency department records, insurance claims, surveys, and the Global Vital Registration system. The DisMod-MR 2.1, a Bayesian mixed-effects meta-regression tool designed by the Institute for Health Metrics and Evaluation (IHME) in Seattle, Washington, United States, was used for modeling and generating estimates of the disease burden across various conditions in 204 countries and territories from 1990 to 2019 (25, 26). It incorporates all the aforementioned available information for each disease and applies established bias correction procedures to derive prevalence and disease burden estimates specific to individual countries, as previously described in prior studies (27).

We extracted data from the Global Health Data Exchange website^1^ for the years 1990 to 2019, stratified by sex, age, and region. This data included CRC-related deaths, DALYs (Disability-Adjusted Life Years), age-standardized mortality rates (ASMR), and age-standardized DALY rates (ASDR). Using the extracted data, we described the epidemiological trends of colorectal cancer burden attributable to diet high in red meat across different sexes, age groups, years, and in different SDI regions.

Additionally, the relationship between socio-demographic index (SDI) and the burden of disease was investigated. SDI is a composite index that considers a country’s *per capita* income, average years of education, and fertility rate. It categorized 204 countries and regions into five groups: low SDI (<0.45), medium SDI (≥0.45 and < 0.61), medium SDI (≥0.61 and < 0.69), medium SDI (≥0.69 and < 0.80), and high SDI (≥0.80) (27, 28).

High red meat consumption in the diet was defined as a daily intake of red meat (beef, pork, lamb, and goat, excluding poultry, fish, eggs, and all processed meats) exceeding 23 g (ranging from 18 g to 27 g) as an optional level (29). Detailed information about inclusion and exclusion criteria can be found in the preceding sections (30). This research aimed to assess the incidence and burden of CRC while exploring its relationship with social demographic indices and diet high in red meat.

### 2.2 Statistical analyses

We analyzed the burden of CRC at the global national level. To account for demographic differences, we used age-standardized incidence rates (ASIR), mortality rates, and Disability-Adjusted Life Years (DALY) rates to better reflect actual incidence and mortality rates. All rates are expressed as per 100,000 people.

Additionally, estimated annual percentage changes (EAPC) were calculated using a linear regression equation: y = α + βx + ε [where y represents ln (ASMR or ASDR), and x represents the calendar year] to assess trends in ASMR and ASDR. The exact calculation is EAPC = 100 (exp(β) - 1) (31). EAPC and its 95% confidence interval (CI) greater than 0 indicate an increasing trend in ASMR or ASDR over the years, while values lower than 0 indicate a decreasing trend. If EAPC is close to 0, it suggests stability (32).

Furthermore, to explore the factors affecting the EAPC of the CRC burden related to a diet high in red meat, we also assessed the correlation between the age-standardized indicator of diet high in red meat in 1990 and SDI in 2019 with EAPC at the national level using the Spearman rank test.

We used the concentration index (CI) and the slope index to quantify the health inequalities. The slope index of inequality and concentration index, are two standard indicators of absolute inequality and relative inequality, respectively (33). The slope index of inequality is calculated by regressing the national DALYs ratio for all age groups on a relative positional scale associated with SDI, defined as the midpoint of the population cumulative range ranked by SDI (34). Heteroscedasticity is explained by a weighted regression model. The concentration index is calculated by numerically integrating the area under the Lorenz concentration curve, which is fitted using the cumulative scores of DALYs and the cumulative relative distribution of the population based on SDI (35).

### 2.3 Bayesian age-period-cohort analysis

Using the GBD data spanning from 1990 to 2019, we conducted forecasts for the disease burden from 2020 to 2030. Our methodology comprised two primary steps: Firstly, we collected data on mortality and DALYs rates for colorectal cancer attributable to diet high in red meat across all age brackets (categorized in 5-year intervals) globally and regionally from 1990 to 2019. Subsequently, by applying a specific formula—the ratio of mortality (or DALYs) cases to the corresponding rate for all age groups in the same year—we recalibrated the annual total populations (36). Following this, we utilized the Bayesian Age-Period-Cohort (BAPC) model to project the disease burden from 2020 to 2030. APC models scrutinize registry data, taking into consideration the individual’s age group, the event’s date (period), and the birth cohort to which the individual belongs (37). Bayesian APC models are especially valuable in projecting the future burden of cancer, as they steer clear of parametric assumptions (38). Bray compared projections from linear power models with both the classical and Bayesian versions of the APC model, concluding that the Bayesian APC model yielded more rational projections (39). All statistical analyses were performed with R software (version 4.2.3).

## 3 Results

### 3.1 Global spatial and temporal colorectal cancer burden attributable to diet high in red meat

**Figure 1:**
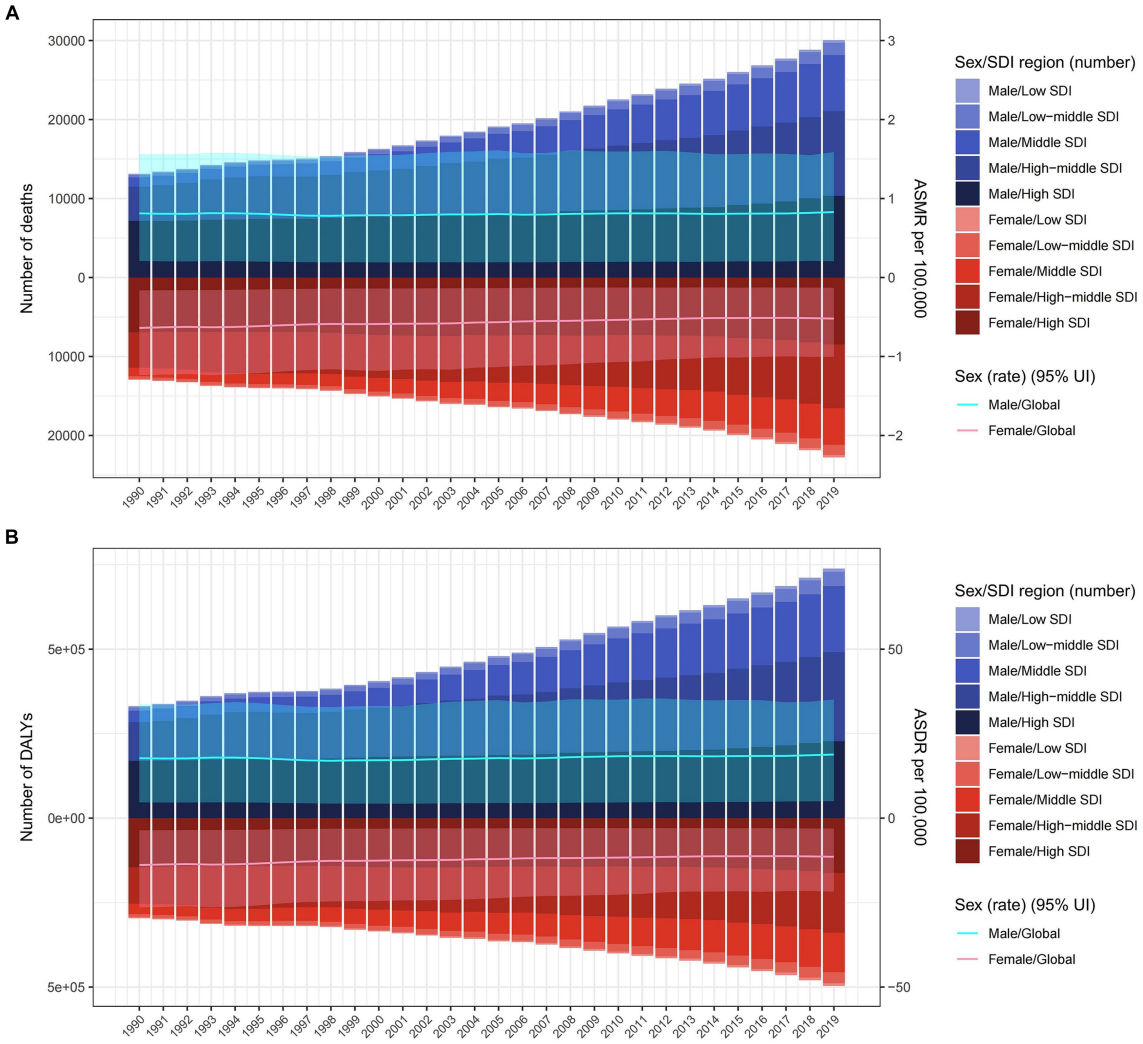
The burden of colorectal cancer deaths **(A)** and DALYs **(B)** due to diet high in red meat from 1990 to 2019 by sex and SDI region. The bar is the number of colorectal cancer deaths and DALYs attributable to diet high in red meat. The line with 95% UI represents ASMR and ASDR attributable to diet high in red meat. ASMR, age-standardized mortality rate; DALYs, disability-adjusted life-years; ASDR, age-standardized DALY rate; UI, uncertainty interval; SDI, sociodemographic index.

Globally, in 1990 and 2019, approximately 0.026 million and 0.052 million deaths from CRC were linked to a diet high in red meat. The male-to-female ratio for these deaths was around 1.3 and 1.5, respectively, for CRC. During this period, there was a rapid increase in deaths and DALYs associated with diet high in red meat. However, the ASMR and ASDR showed little change, with EAPC in ASMR for females being less than 0, and greater than 0 for males (Tables 1, 2; Figure 1).

**Table 1:** Deaths of colon and rectum cancer attributable to diet high in red meat in 1990 and 2019 for both sexes and all locations, with estimated annual percentage change from 1990 to 2019.

Location	Deaths cases in 1990	ASMR per 100,000 in 1990	Deaths cases in 2019	ASMR per 100,000 in 2019	EAPC (1990–2019)
Global Both	26,087 (6,690 to 50,231)	0.71 (0.18 to 1.38)	52,811 (13,598 to 100,688)	0.66 (0.17 to 1.26)	−0.32% (−0.37 to −0.28)
Female	12,951 (3,302 to 24,856)	0.64 (0.16 to 1.23)	22,760 (5,663 to 44,004)	0.52 (0.13 to 1.01)	−0.83% (−0.88 to −0.78)
Male	13,136 (3,427 to 25,156)	0.81 (0.21 to 1.56)	30,051 (7,627 to 57,328)	0.83 (0.21 to 1.59)	0.07% (0.01 to 0.12)
Region High SDI	14,063 (3,944 to 25,419)	1.34 (0.38 to 2.42)	18,849 (4,893 to 35,349)	0.97 (0.26 to 1.79)	−1.29% (−1.37 to −1.21)
High-middle SDI	8,864 (2,276 to 17,225)	0.87 (0.22 to 1.7)	18,793 (5,033 to 35,132)	0.93 (0.25 to 1.74)	0.09% (0.01 to 0.17)
Middle SDI	2,278 (297 to 5,662)	0.23 (0.03 to 0.59)	11,723 (2,560 to 23,587)	0.49 (0.1 to 0.99)	2.95% (2.77 to 3.12)
Low-middle SDI	649 (122 to 1,497)	0.12 (0.02 to 0.27)	2,826 (617 to 5,904)	0.22 (0.05 to 0.45)	2.38% (2.28 to 2.48)
Low SDI	221 (32 to 565)	0.1 (0.01 to 0.26)	598 (98 to 1,464)	0.12 (0.02 to 0.3)	0.81% (0.72 to 0.9)
Central Sub-Saharan Africa	27 (5 to 67)	0.13 (0.02 to 0.32)	61 (10 to 150)	0.12 (0.02 to 0.3)	−0.18% (−0.51 to 0.15)
East Asia	2,851 (408 to 6,970)	0.34 (0.05 to 0.84)	16,498 (4,056 to 31,569)	0.82 (0.2 to 1.58)	3.6% (3.35 to 3.86)
Eastern Europe	2,995 (772 to 5,768)	1.07 (0.28 to 2.07)	2,708 (468 to 5,947)	0.79 (0.14 to 1.72)	−1.91% (−2.21 to −1.62)
Eastern Sub-Saharan Africa	88 (10 to 231)	0.12 (0.01 to 0.32)	241 (27 to 626)	0.16 (0.02 to 0.4)	0.9% (0.79 to 1.02)
Andean Latin America	36 (4 to 92)	0.18 (0.02 to 0.47)	154 (17 to 392)	0.28 (0.03 to 0.71)	1.81% (1.68 to 1.95)
High-income Asia Pacific	910 (87 to 2,421)	0.47 (0.05 to 1.25)	2,196 (275 to 5,546)	0.47 (0.06 to 1.14)	−0.25% (−0.35 to −0.15)
High-income North America	4,798 (1,332 to 8,719)	1.36 (0.38 to 2.44)	6,386 (1712 to 11,567)	1.02 (0.28 to 1.82)	−1.21% (−1.33 to −1.09)
Caribbean	83 (8 to 220)	0.33 (0.03 to 0.87)	209 (20 to 560)	0.4 (0.04 to 1.08)	0.9% (0.79 to 1.01)
Australasia	630 (276 to 959)	2.72 (1.19 to 4.15)	888 (367 to 1,387)	1.75 (0.74 to 2.71)	−1.8% (−1.97 to −1.63)
Central Europe	1,538 (322 to 3,190)	1.06 (0.22 to 2.23)	2,862 (699 to 5,678)	1.33 (0.33 to 2.61)	1.04% (0.92 to 1.17)
Central Latin America	189 (24 to 461)	0.24 (0.03 to 0.58)	807 (130 to 1946)	0.34 (0.05 to 0.84)	1.38% (1.33 to 1.42)
Central Asia	271 (70 to 524)	0.57 (0.14 to 1.11)	389 (98 to 756)	0.56 (0.14 to 1.11)	0.39% (−0.13 to 0.91)
North Africa and Middle East	301 (29 to 820)	0.18 (0.02 to 0.5)	882 (86 to 2,352)	0.22 (0.02 to 0.57)	0.76% (0.51 to 1.02)
Oceania	6 (1 to 16)	0.22 (0.03 to 0.59)	15 (2 to 41)	0.24 (0.03 to 0.64)	0.11% (−0.02 to 0.24)
South Asia	267 (70 to 583)	0.05 (0.01 to 0.12)	947 (228 to 2,161)	0.07 (0.02 to 0.16)	0.84% (0.71 to 0.97)
Southeast Asia	406 (51 to 1,036)	0.17 (0.02 to 0.42)	1822 (220 to 4,550)	0.31 (0.04 to 0.77)	2.15% (2.08 to 2.21)
Southern Latin America	896 (376 to 1,387)	2 (0.82 to 3.09)	1768 (705 to 2,777)	2.1 (0.84 to 3.3)	0.15% (0.06 to 0.23)
Southern Sub-Saharan Africa	83 (11 to 211)	0.32 (0.04 to 0.84)	209 (30 to 498)	0.4 (0.06 to 0.95)	1.01% (0.84 to 1.18)
Tropical Latin America	388 (78 to 834)	0.45 (0.09 to 0.98)	2,403 (890 to 3,925)	1 (0.36 to 1.64)	3.02% (2.41 to 3.64)
Western Europe	9,232 (2,731 to 16,476)	1.59 (0.48 to 2.82)	11,104 (2,953 to 20,565)	1.16 (0.32 to 2.11)	−1.33% (−1.44 to-1.22)
Western Sub-Saharan Africa	92 (10 to 244)	0.11 (0.01 to 0.3)	262 (29 to 684)	0.16 (0.02 to 0.41)	1.35% (1.23 to 1.48)

The deaths cases and ASMR of colon and rectum cancer attributable to diet high in red meat in 1990 and 2019 for all regions were directly downloaded from the official website (https://vizhub.healthdata.org/gbd-results/) of the GBD study 2019. The EAPC of ASMR were additional calculations in this study. ASMR, age-standardized mortality rate; EAPC, estimated annual percentage change.

**Table 2:** DALYs of colon and rectum cancer attributable to diet high in red meat in 1990 and 2019 for both sexes and all locations, with estimated annual percentage change from 1990 to 2019.

Location	DALYs in 1990	ASDR per 100,000 in 1990	DALYs in 2019	ASDR per 100,000 in 2019	EAPC (1990–2019)
Global Both	627,834 (165,197 to 1,192,278)	15.63 (4.08 to 29.8)	1,234,678 (332,704 to 2,306,844)	14.95 (4.02 to 27.99)	−0.18% (−0.25 to −0.11)
Female	296,273 (76,863 to 562,565)	13.86 (3.6 to 26.4)	496,397 (132,360 to 944,785)	11.43 (3.05 to 21.75)	−0.77% (−0.84 to −0.71)
Male	331,561 (88,528 to 627,390)	17.73 (4.67 to 33.68)	738,281 (198,240 to 1,373,853)	18.83 (5.03 to 35.12)	0.24% (0.16 to 0.32)
Region High SDI	314,924 (92,479 to 557,564)	30.93 (9.16 to 54.6)	389,921 (108,477 to 703,495)	22.76 (6.57 to 40.43)	−1.2% (−1.27 to −1.12)
High-middle SDI	221,808 (58,212 to 422,210)	20.29 (5.29 to 38.8)	440,051 (124,026 to 802,903)	21.86 (6.2 to 39.94)	0.09% (−0.01 to 0.19)
Middle SDI	65,863 (8,875 to 161,394)	5.77 (0.75 to 14.25)	311,976 (73,750 to 608,407)	11.96 (2.77 to 23.44)	3% (2.82 to 3.19)
Low-middle SDI	18,653 (3,612 to 42,686)	2.83 (0.54 to 6.52)	75,490 (17,505 to 155,025)	5.22 (1.19 to 10.78)	2.39% (2.29 to 2.49)
Low SDI	6,319 (893 to 16,233)	2.43 (0.35 to 6.21)	16,780 (2,643 to 41,401)	2.92 (0.47 to 7.18)	0.73% (0.64 to 0.82)
Central Sub-Saharan Africa	796 (141 to 1950)	3.15 (0.57 to 7.77)	1771 (274 to 4,466)	2.92 (0.46 to 7.19)	−0.17% (−0.48 to 0.14)
East Asia	83,024 (12,430 to 200,519)	8.61 (1.25 to 20.89)	436,252 (115,075 to 813,024)	20.8 (5.44 to 38.62)	3.72% (3.44 to 3.99)
Eastern Europe	77,518 (20,849 to 145,612)	27.49 (7.36 to 51.58)	62,825 (11,277 to 137,394)	18.92 (3.42 to 41.18)	−2.2% (−2.53 to −1.88)
Eastern Sub-Saharan Africa	2,553 (286 to 6,777)	3.04 (0.35 to 8)	6,912 (738 to 18,081)	3.71 (0.41 to 9.69)	0.79% (0.68 to 0.91)
Andean Latin America	912 (96 to 2,357)	4.18 (0.44 to 10.82)	3,690 (443 to 9,188)	6.43 (0.76 to 16.05)	1.8% (1.66 to 1.93)
High-income Asia Pacific	22,154 (2,126 to 59,218)	10.88 (1.05 to 29.1)	41,772 (5,719 to 101,560)	10.94 (1.65 to 25.79)	−0.2% (−0.32 to −0.08)
High-income North America	110,891 (32,929 to 193,179)	32.99 (9.96 to 57.08)	144,668 (42,179 to 251,558)	25.35 (7.51 to 43.92)	−1.08% (−1.19 to −0.96)
Caribbean	1956 (185 to 5,166)	7.39 (0.7 to 19.52)	4,608 (437 to 12,277)	8.91 (0.85 to 23.75)	0.87% (0.75 to 0.98)
Australasia	14,698 (6,625 to 22,077)	63.82 (28.97 to 95.77)	18,262 (7,951 to 28,011)	39.61 (17.84 to 60.29)	−1.91% (−2.08 to −1.74)
Central Europe	37,988 (8,506 to 76,660)	25.72 (5.77 to 51.88)	63,016 (16,256 to 122,005)	31.44 (8.24 to 60.21)	0.99% (0.84 to 1.14)
Central Latin America	5,030 (675 to 11,890)	5.5 (0.72 to 13.21)	20,610 (3,627 to 48,284)	8.47 (1.47 to 19.89)	1.59% (1.54 to 1.65)
Central Asia	8,019 (2,143 to 15,197)	15.9 (4.21 to 30.17)	10,914 (2,929 to 21,059)	13.59 (3.49 to 26.08)	−0.19% (−0.7 to 0.33)
North Africa and Middle East	8,526 (802 to 23,203)	4.5 (0.43 to 12.31)	23,444 (2,225 to 62,629)	4.98 (0.48 to 13.24)	0.51% (0.25 to 0.77)
Oceania	187 (23 to 484)	5.47 (0.66 to 14.24)	454 (49 to 1,206)	5.65 (0.59 to 15.03)	−0.02% (−0.14 to 0.1)
South Asia	7,509 (2045 to 16,221)	1.23 (0.32 to 2.67)	24,347 (5,853 to 55,572)	1.66 (0.4 to 3.79)	0.86% (0.73 to 0.99)
Southeast Asia	11,633 (1,431 to 29,882)	4.08 (0.51 to 10.45)	49,492 (5,926 to 123,345)	7.56 (0.91 to 18.86)	2.1% (2.04 to 2.17)
Southern Latin America	20,605 (9,149 to 31,548)	44.3 (19.61 to 67.88)	38,064 (16,077 to 58,942)	46.7 (19.81 to 72.14)	0.17% (0.1 to 0.24)
Southern Sub-Saharan Africa	2,216 (302 to 5,423)	7.39 (0.98 to 18.32)	5,485 (878 to 12,736)	9.1 (1.4 to 21.36)	1.09% (0.91 to 1.27)
Tropical Latin America	10,572 (2,212 to 22,079)	10.65 (2.16 to 22.52)	61,167 (23,985 to 97,095)	24.61 (9.58 to 39.24)	3.15% (2.49 to 3.81)
Western Europe	198,617 (60,793 to 339,982)	36.14 (11.18 to 61.52)	210,073 (59,872 to 372,018)	25.63 (7.6 to 44.82)	−1.42% (−1.51 to −1.34)
Western Sub-Saharan Africa	2,429 (262 to 6,498)	2.61 (0.29 to 6.94)	6,853 (714 to 18,052)	3.39 (0.37 to 8.86)	1.16% (1.04 to 1.28)

The DALYs and ASDR of colon and rectum cancer attributable to diet high in red meat in 1990 and 2019 for all regions were directly downloaded from the official website (https://vizhub.healthdata.org/gbd-results/) of the GBD study 2019. The EAPC of ASDq22R were additional calculations in this study. DALYs, disability-adjusted life years; ASDR, age-standardized disability adjusted life year rate; EAPC, estimated annual percentage change.

On a global scale, regions with high SDI had the most deaths related to diet high in red meat (0.018 million) in 2019, while high-middle SDI regions had the highest DALYs (0.44 million), together accounting for over 35% of cases worldwide. However, the highest ASMR and ASDR were observed in high SDI regions. Over the years, ASMR and ASDR increased in low, low-middle, middle, and high-middle SDI regions, with middle and low-middle SDI regions experiencing a faster increase compared to low and high-middle SDI regions. In contrast, high SDI regions had a significant decrease in ASMR [EAPC −1.29% (95% CI: −1.37 to −1.21)] and ASDR [EAPC −1.2% (95% CI: −1.27 to −1.12)] (Tables 1, 2).

At the regional level according to the Global Burden of Disease (GBD) classification, East Asia carried the heaviest burden in 2019, with 0.016 million deaths and 0.43 million DALYs worldwide. However, Australasia had the highest ASMR (1.75 per 100,000) and ASDR (39.61 per 100,000). The most significant increase in ASMR and ASDR from 1990 to 2019 was observed in Tropical Latin America and East Asia, with Estimated Annual Percent Changes (EAPCs) exceeding 3 in both regions. On the other hand, Eastern Europe experienced the most substantial decrease in ASMR [EAPC −1.91% (95% CI: −2.21 to −1.62)] and ASDR [EAPC −2.2% (95% CI: −2.53 to −1.88)] (Tables 1, 2).

**Figure 2:**
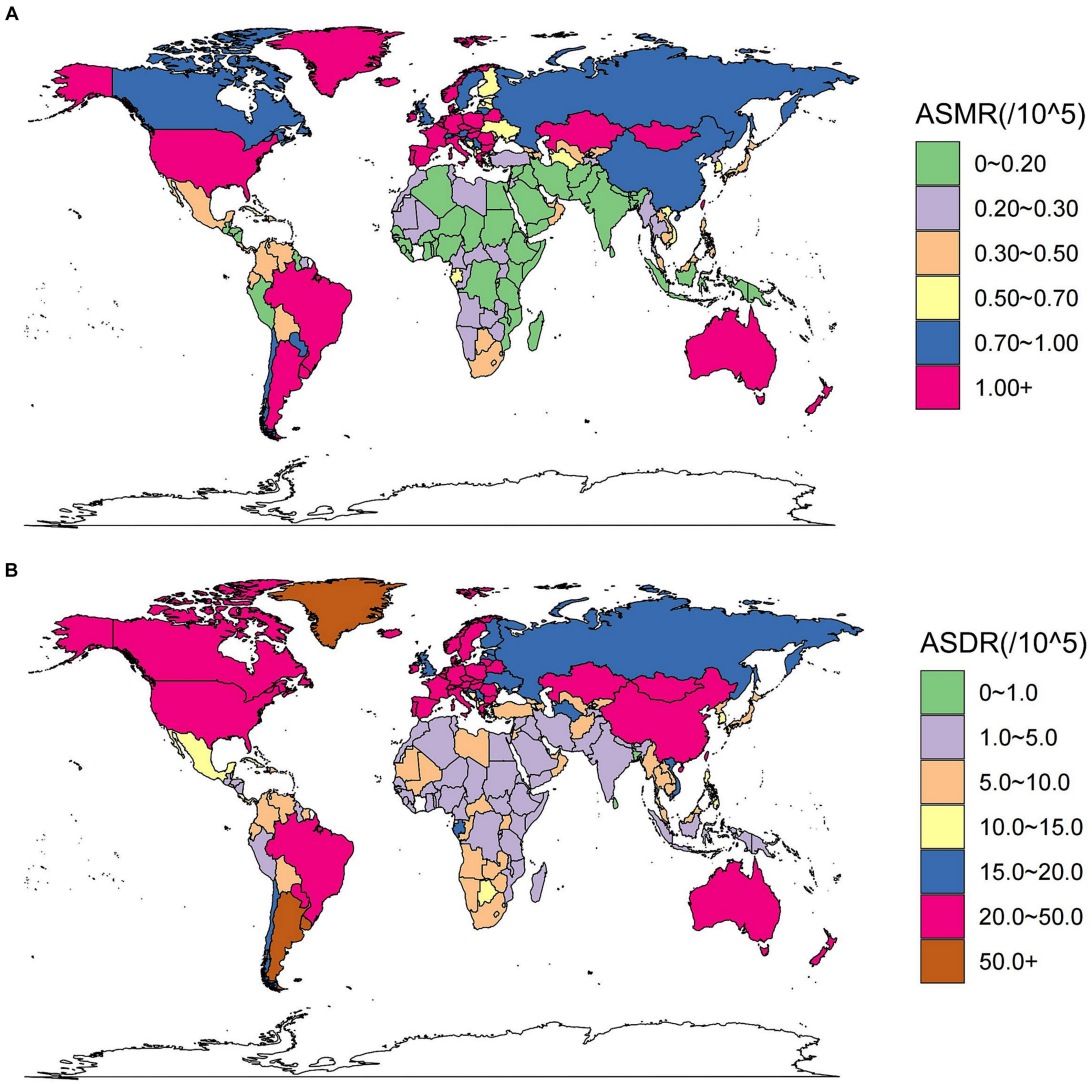
These maps show the ASMR **(A)** and ASDR **(B)** of colorectal cancer due to diet high in red meat per 100,000 people in 2019 in 204 countries and territories, for both sexes. ASMR, age-standardized mortality rate; ASDR, age-standardized DALY rate.

At country level, China had the highest number of CRC deaths and DALYs attributed to a high red meat diet in 2019 (Supplementary Tables S1, S2). Argentina and Greenland had the highest ASMR and ASDR in 2019 (Figures 2A,B; Supplementary Tables S1, S2). However, the most substantial increase in ASMR and ASDR occurred in Equatorial Guinea, with EAPCs of 4.71% (95% CI: 4.27 to 5.14) and 4.31% (95% CI: 3.9 to 4.72), respectively (Supplementary Figure S1; Tables S1, S2).

### 3.2 Global colorectal cancer burden attributable to diet high in red meat by age and sex

**Figure 3:**
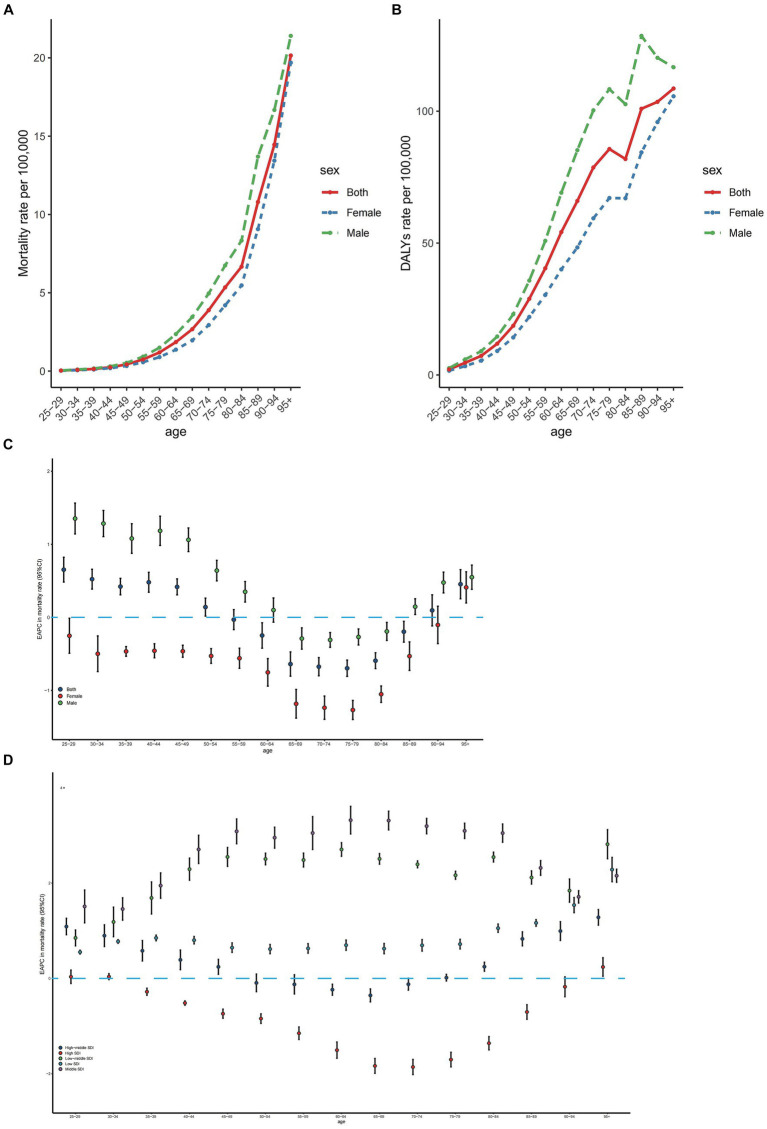
Age-specific rates of mortality **(A)** and DALYs **(B)** of colorectal cancer due to diet high in red meat by sex, 2019 and the age distribution of colorectal cancer due to diet high in red meat trend in mortality rate from 1990 to 2019 by sex **(C)** and location **(D)**. EAPC, estimated annual percentage change; SDI, sociodemographic index; DALYs, disability-adjusted life-years.

In 2019, the number of deaths from CRC attributed to diet high in red meat exhibited a synchronous pattern in males and females, first rising and then declining with age. The peak age of incidence was in the 70–74 years group (Supplementary Figure S2). More deaths were observed in the 65–74 years age range, with a higher number of age-specific deaths in males compared to females (Supplementary Figure S2). Consequently, the age-specific mortality rate in males was greater than that in females, and showed a rapid increase for both sexes (Figure 3A).

The age-specific number of colorectal cancer DALYs followed a similar pattern to that of deaths, but the peak point appeared in the 65–69 years age group, with more DALYs occurring in the 60–69 years age range (Supplementary Figure S2). The age-specific number of CRC DALYs was significantly higher in males than in females (Supplementary Figure S2). Correspondingly, the age-specific DALY rate in males was larger than that in females. The trend of the age-specific DALY rate resembled that of the mortality rate but declined in the 80–84 years age group (Figure 3B).

Globally, the age-specific mortality rate has increased in the 25–49 years and over 95 years age groups for both sexes from 1990 to 2019, with the most rapid increase occurring in the 25–29 years age group (Figure 3C). In males, the age-specific mortality rate rose in the 25–59 years and over 85 years age groups from 1990 to 2019, but fell in the 65–84 years age group, with the most significant increases and decreases occurring in the 65–69 years and 70–74 years age groups, respectively (Figure 3C). Conversely, the age-specific mortality rates in females declined in most age groups (Figure 3C). The age-specific mortality rates increased in low, low-middle, and middle SDI regions from 1990 to 2019. In high SDI regions, the age-specific mortality rates dropped in the 35–89 years age range, followed by an increase in older age groups. In high-middle SDI regions, the age-specific mortality rates decreased in the 60–69 years age group and increased in relatively younger and older age groups (Figure 3D). The EAPCs in age-specific DALY rates exhibited a similar pattern to that in age-specific mortality rates (Supplementary Figure S3).

### 3.3 The association between SDI, global inequalities, and the burden of colorectal cancer burden attributable to diet high in red meat

**Figure 4:**
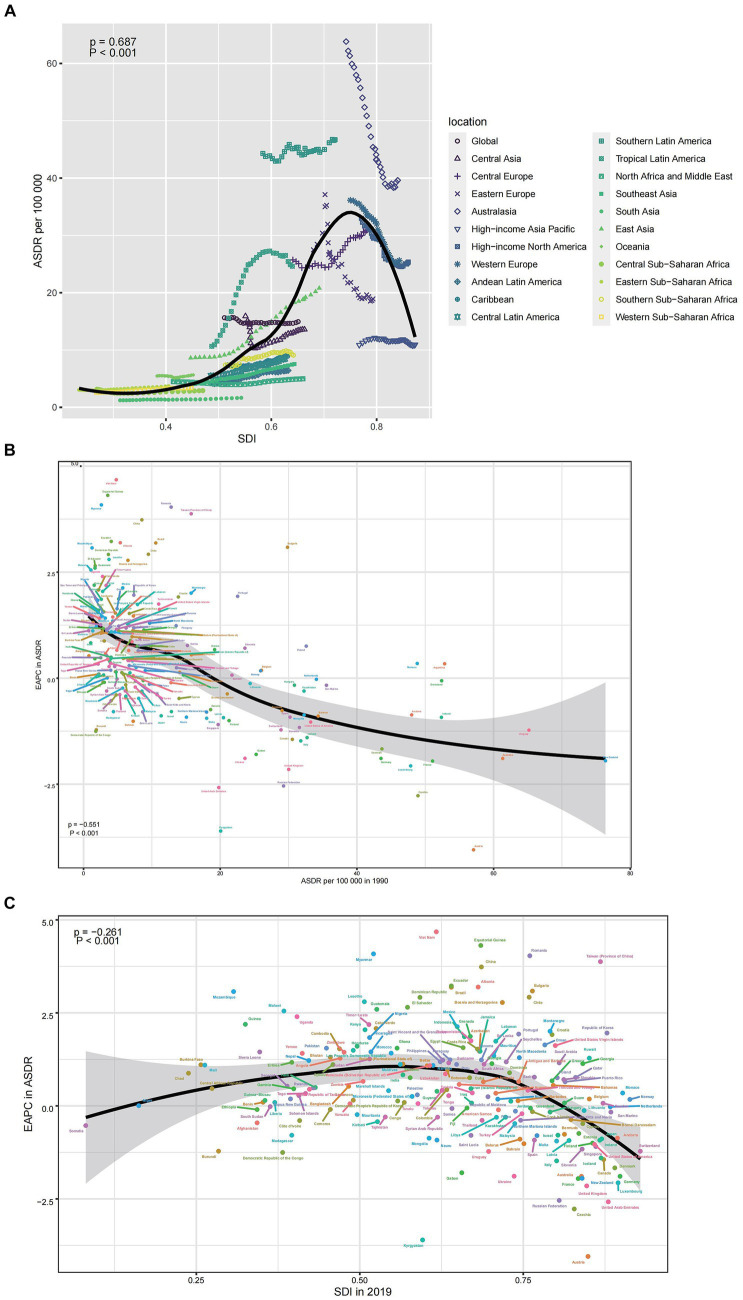
The correlation between colorectal cancer due to diet high in red meat in ASMR and SDI **(A)**, between EAPC in ASMR and ASMR in 1990 **(B)**, and between EAPC in ASMR and SDI in 2019 **(C)**. EAPC, estimated annual percentage change; ASMR, age-standardized mortality rate; SDI, sociodemographic index.

In general, the ASMR displayed an inverted V-shaped correlation with the SDI, reaching its peak around an SDI value of approximately 0.75 (Figure 4A). The EAPC in ASMR exhibited a strong negative correlation with ASMR values in 1990 (ρ = −0.551, *p* < 0.001), particularly when ASMR was low (Figure 4B). Furthermore, the EAPC in ASMR showed a negative association with SDI values in 2019 (ρ = −0.216, *p* < 0.001), particularly for SDI values exceeding 0.75 (Figure 4C). Similar patterns were also evident when considering ASDR and their correlation with SDI, as well as the relationship between EAPC in ASDR and ASMR in 1990 and SDI in 2019 (Supplementary Figure S2).

**Figure 5:**
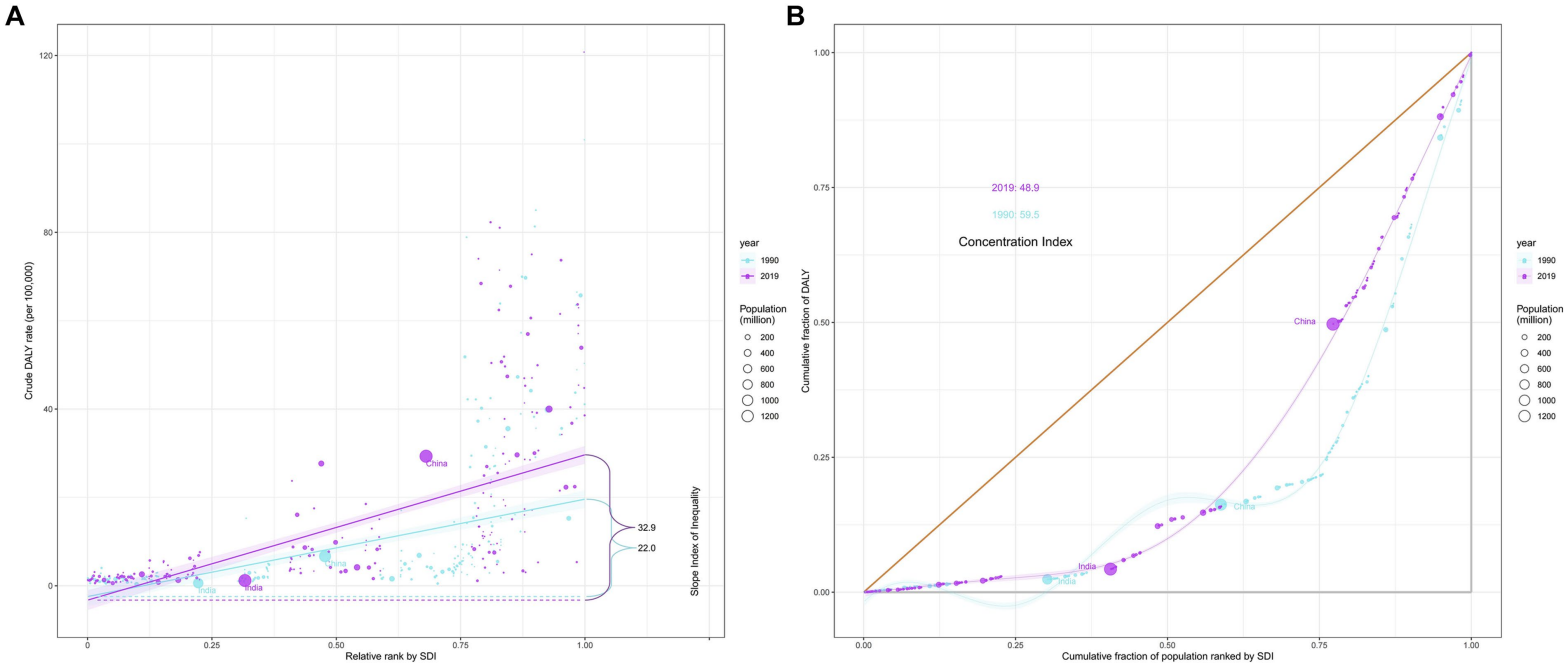
Income-related health inequality regression **(A)** and concentration curves **(B)** for DALYs of colorectal cancer due to diet high in red meat across 204 counties and territories, 1990 and 2019. DALYs, disability-adjusted life-years.

In 1990 and 2019, 204 countries and territories experienced significant income-related inequalities in DALYs caused by CRC, the slope index of inequality in the DALYs rate between the highest and the lowest SDI country increased from 22.0 (95% CI 18.1 to 25.9) in 1990 to 32.9 (95% CI 28.3 to 37.5) in 2019 (Figure 5A; Table 3). The concentration index was 59.5 (95% CI 46.4 to 72.6) in 1990 and 48.9 (95% CI 34.6 to 63.1) in 2019 indicating that the burden was disproportionately concentrated in more-affluent countries (Figure 5B; Table 3).

**Table 3:** Summary measures for cross-country inequalities related to SDI in DALYs of colorectal cancer burden attributable to diet high in red meat.

Diseases	Health inequality metrics	Year	Value	95% CI
colon and rectum cancer	Slope index of inequality	1990	22.0	28.3 to 37.5
		2019	32.9	18.1 to 25.9
	Concentration index	1990	59.5	46.4 to 72.6
		2019	48.9	34.6 to 63.1

Concentration index was executed using the Health Equity Assessment Toolkit from WHO and slope index of inequality was executed using R software (V.4.2.1). WHO, World Health Organization.

### 3.4 Colorectal cancer burden attributable to diet high in red meat death and DALY rate projections till 2030

**Figure 6:**
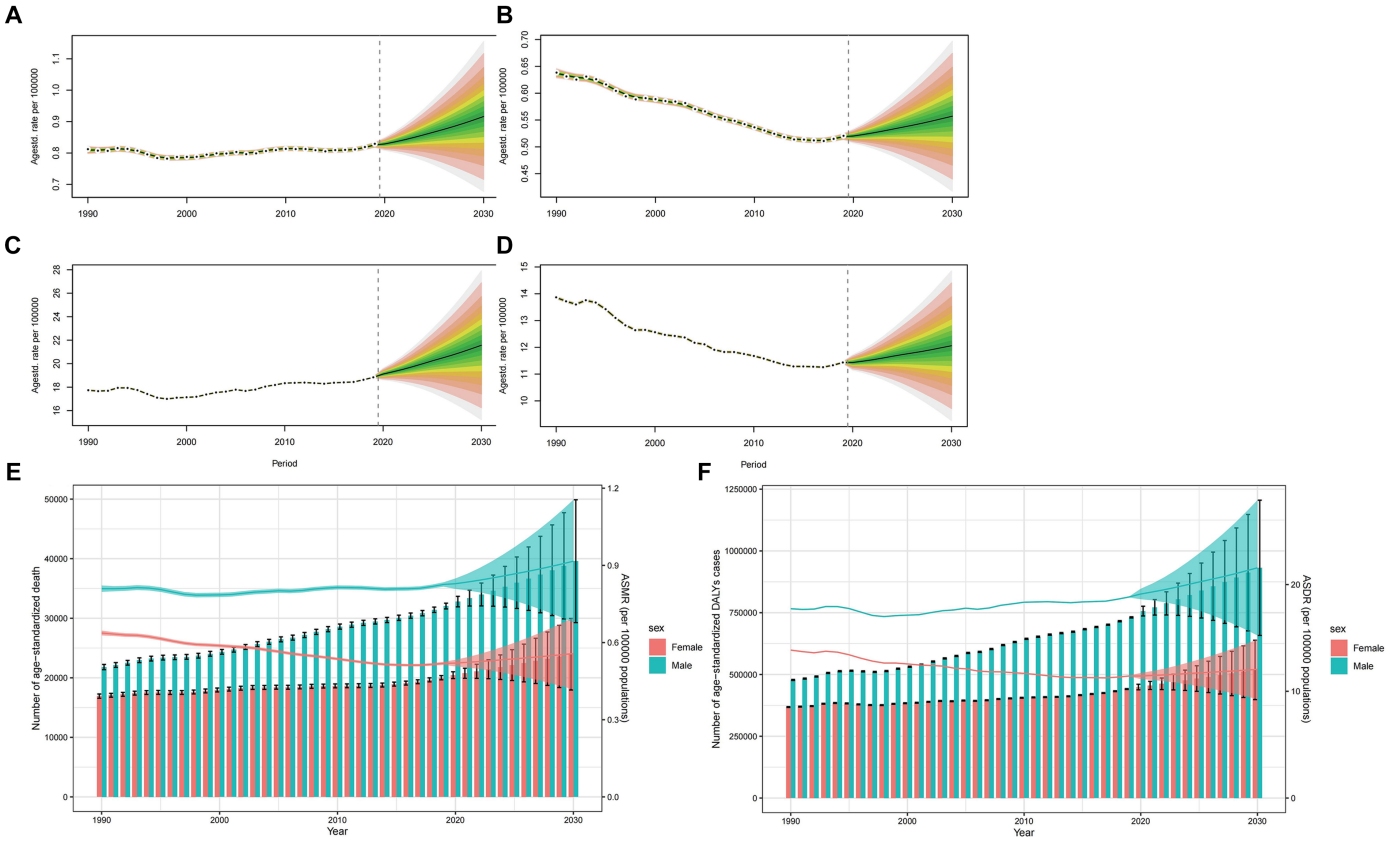
Projections of ASMR **(A,B)** and ASDR **(C,D)** in males and females from 2020 to 2030. The open dot represents the observed value, and the fan represents the predicted distribution between the 2.5 and 97.5% quantiles. The forecast average is shown as a solid line. The vertical dotted line indicates where the prediction begins. The projections of deaths **(E)** and DALYs **(F)** by sexes of colorectal cancer due to diet high in red meat from 2020 to 2030. The error bar denotes the 95% credible interval of the predictive value.

Globally, ASMR is poised to rise for both sexes, yet more markedly for males (Figures 6A,B). While the age specific mortality rate will notably peak in groups aged over 95 years, the mortality rate in this group is projected to experience a downturn from 2020 to 2030 (Supplementary Figures S5, S6). Concurrently, except for people over 95 years, the majority of age groups for both males and females will be observed an increasing trend. From 2020 to 2030, the anticipated mortality cases will grow by years, with male mortality cases significantly surpassing that of females (Figure 6E). As for the trend of ASDR, age specific DALYs rate and DALYs cases, it has similar pattern to that in ASMR, age specific mortality rate and mortality cases of the world (Figures 6C,D,F; Supplementary Figures S7, S8).

## 4 Discussion

Our research analyzed the global burden of CRC caused by high red meat consumption in diets from 1990 to 2019, stratified by factors such as year, age, sex, region, and sociodemographic index. Our findings revealed a consistent global rise in mortality and DALYs rate linked to CRC due to elevated red meat consumption. This indicates that the burden it imposes remains a significant global public health concern.

In the 2019 report, there were over 50,000 reported cases of death, with the elderly being a high-risk group. Although the age-standardized death rate related to high red meat consumption in diets and CRC has declined from 1990 to 2019, the absolute number of fatalities has more than doubled. Population growth and age demographic shifts might account for this rise. Furthermore, the burden is closely related to socioeconomic development and how it is distributed unevenly. As high and high-middle SDI regions shoulder a greater burden, their ASDR and ASMR consistently decrease annually. Specifically, Western Europe has seen significant reductions in ASMR and ASDR. Conversely, there has been a concerning increase in low SDI regions, such as tropical Latin America. No doubt, economic growth has brought more affordable red meat and shifted the disease burden pattern in low and middle-income countries from infectious diseases, maternal and neonatal diseases, and nutritional diseases to non-communicable chronic diseases (25, 40), with CRC importantly of noteworthy. Additionally, the escalating male-to-female ratio in CRC-related deaths indicates that males are being affected to a greater extent. The reason for this may be that males are more likely to consume unprocessed red meat, leading to higher estimated intake levels (41). Another hypothesis is that dietary-related effects may vary by sex due to hormonal differences between males and females, as well as the tendency for females to develop proximal tumors and males to develop distal and rectal tumors (42).

Meat processing, preservation, and high-temperature cooking can yield carcinogens such as HCAs, PAHs, and NOCs, which are implicated in the onset of several cancers (43–46). Furthermore, red meat is a major source of heme iron. Many epidemiological and evidence-based studies have suggested a possible association between the intake of heme iron and the risk of various cancers, including colorectal cancer (47, 48). Compared to low-income countries, high-income countries have a red meat consumption level that is approximately five times higher (49). Despite the health inequality analysis indicating that the burden is disproportionately concentrated in wealthier countries, high-income countries have exhibited reduced red meat consumption over recent decades. This decline is primarily due to the development of relevant dietary guidelines and an increased level of compliance with these guidelines. Additionally, people are becoming more aware of the negative health effects of red meat consumption (50–52). Another study has demonstrated the merit of public health initiatives that limit red meat intake in developed countries (53). Therefore, high-income countries should emphasize the benefits of a healthy diet and use evidence-based intervention measures to educate and empower individuals to understand the risks associated with excessive red meat consumption (24, 54). At the same time, although the burden in developing countries is relatively smaller, deaths related to CRC are rapidly increasing. From a nutritional perspective, animal-source foods (ASF) including red meat remain important in developing countries (49). In these nations, it might not be necessary to completely avoid the consumption of red meat (13, 55, 56). Most African countries are low-income or middle-income nations and their people do not consume red meat, even at potentially beneficial levels and this can contribute to severe nutritional deficiencies associated with a low life expectancy (57, 58). For such countries, vigilance should not be relaxed, as paradoxically, excessive red meat consumption can eventually burden these nations (23). Policymakers must remain cognizant of this risk.

The red meat consumption in different countries also has an interaction on general diet patterns. In East Asian countries such as Japan and Korea, where red meat consumption has historically been low compared to Western countries, diets have traditionally centered on rice, vegetables, tofu, and seafood. The influence of Buddhism in some East Asian countries has promoted vegetarianism or restricted meat consumption, further reducing red meat intake (59). In some Nordic countries such as Sweden, Norway, Denmark, Finland, and Iceland, their traditional diets are rich in fish, whole grains, dairy products, and vegetables, and red meat consumption has traditionally been low. Fish, especially fatty fish such as salmon, herring, and mackerel, is an important part of Nordic cuisine. Recent trends in these countries to promote sustainable and locally sourced food may further encourage the consumption of fish and vegetables rather than red meat (60). Countries with specific cultural or religious rules on red meat consumption, such as India and certain Buddhist-influenced Southeast Asian countries, typically have diets that prioritize vegetables, legumes, grains, and dairy products over red meat. In India, where cow slaughter is banned in many states due to Hindu reverence for cows, beef consumption is minimal (61). Similarly, in Buddhist-majority countries such as Thailand and Vietnam, where many people follow a vegetarian diet on certain religious occasions, red meat consumption tends to be low.

Furthermore, though excessive red meat consumption is unhealthy, our study did not directly link it to CRC causality. We could only delineate the present burden and trends. Our study utilized data from GBD 2019, which primarily consists of national and regional data. The accuracy of our research hinges on the GBD study’s data quality and volume. We lack individual-level data to support our findings. And because there was not data for each subgroup of CRC and for CRC at different sites in the GBD database, we were only able to obtain combined data. The clarity and depth of the analysis could not be further improved. In addition, the highest consumers of red meat (the top 20% of red meat consumers) also have the highest body mass index, are more likely to smoke, engage in less physical activity, generally have lower levels of education and health literacy, consume smaller quantities of fruits and vegetables, and have higher daily calorie intake. Any or a combination of these residual confounding factors could potentially influence the association between red meat intake and colorectal cancer (17).

## 5 Conclusion

Although the ASMR and ASDR of CRC associated with diet high in red meat have decreased globally from 1990 to 2019, the absolute number of cases is still on the rise. The number of deaths has more than doubled since 1990, with a greater impact on males and older individuals. CRC linked to diet high in red meat exhibited significant income-related inequality, disproportionately burdening more affluent countries. However, in developing countries, including some countries in tropical Latin America and Africa, ASMR and ASDR related to cancer were higher. Moreover, in accordance with our projections, global ASMR and ASDR might tend to increase until 2030. We hope that our research can provide insights for policymakers to tailor dietary guidelines, disease control, and prevention strategies based on the specific circumstances of different countries, especially for least developed countries with relatively lower economic levels.

## Data availability statement

The raw data supporting the conclusions of this article will be made available by the authors, without undue reservation.

## Author contributions

XY: Writing – review & editing, Software, Investigation, Data curation. DW: Writing – review & editing, Supervision, Investigation, Data curation. YL: Writing – review & editing, Investigation, Data curation. ZH: Writing – review & editing, Investigation, Data curation. AM: Writing – review & editing, Supervision, Formal analysis. HF: Writing – review & editing, Supervision, Investigation, Data curation, Conceptualization. HX: Writing – review & editing, Writing – original draft, Supervision, Project administration, Funding acquisition, Formal analysis, Data curation, Conceptualization.

## Footnotes

^1^ http://ghdx.healthdata.org/gbd-results-tool
