# Supplementary figures and images for: Global disease burden linked to diet high in red meat and colorectal cancer from 1990 to 2019 and its prediction up to 2030
Source: Front Nutr. 2024 Mar 14;11:1366553. doi: 10.3389/fnut.2024.1366553 (PMC10973012; doi:10.3389/fnut.2024.1366553)

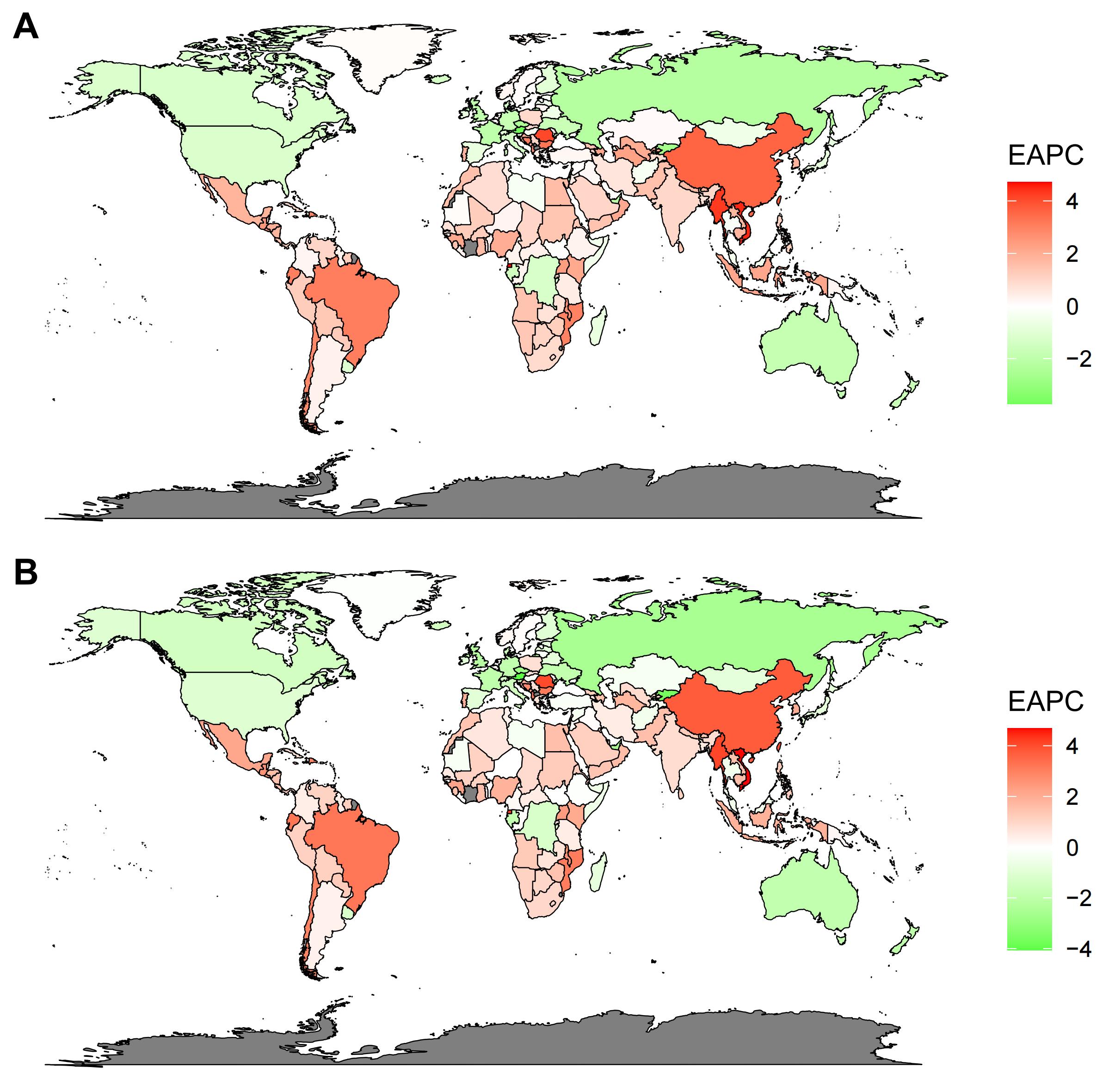

Supplement: Supplementary file 1 [file Image_1.JPEG]

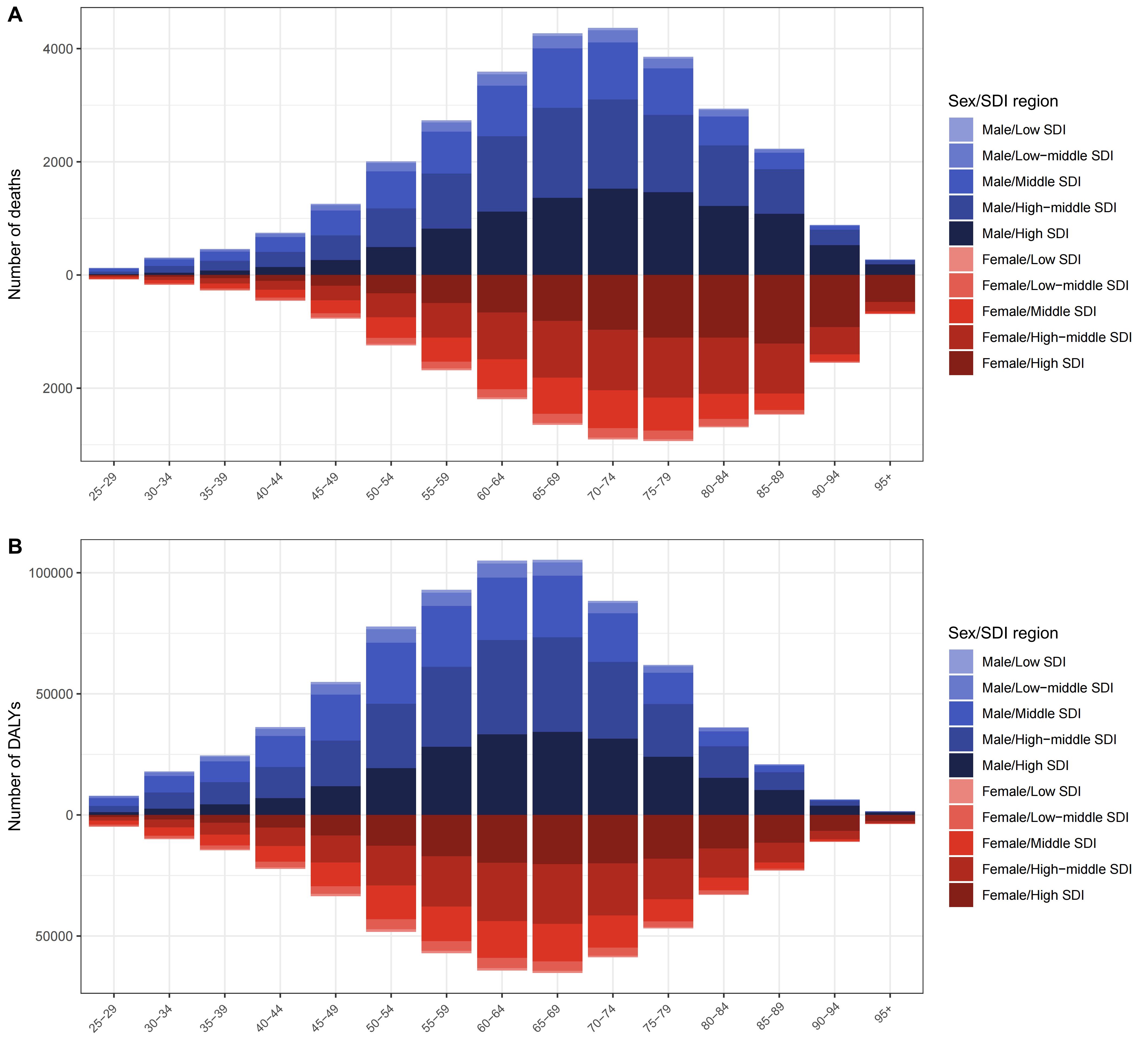

Supplement: Supplementary file 2 [file Image_2.JPEG]

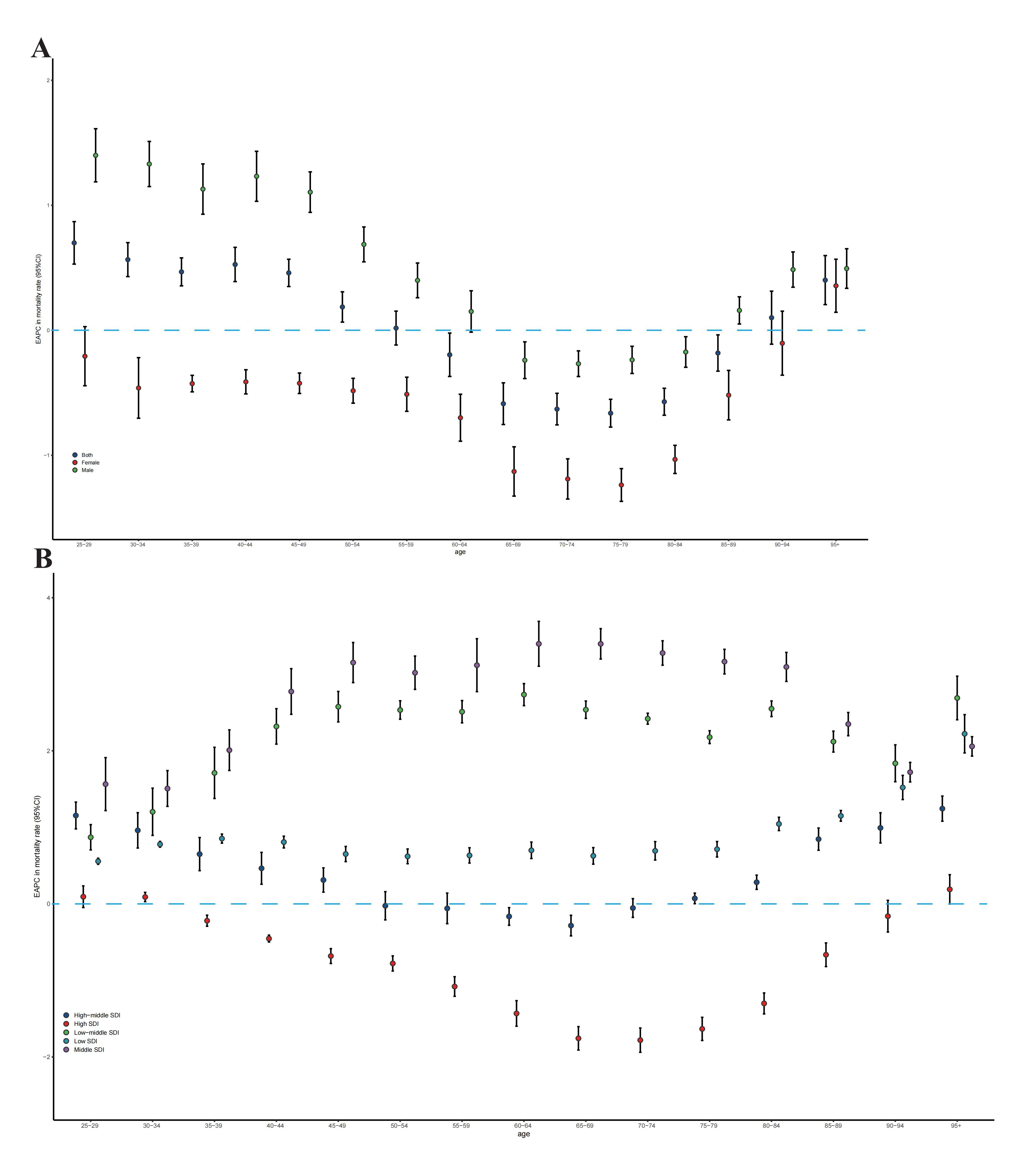

Supplement: Supplementary file 3 [file Image_3.JPEG]

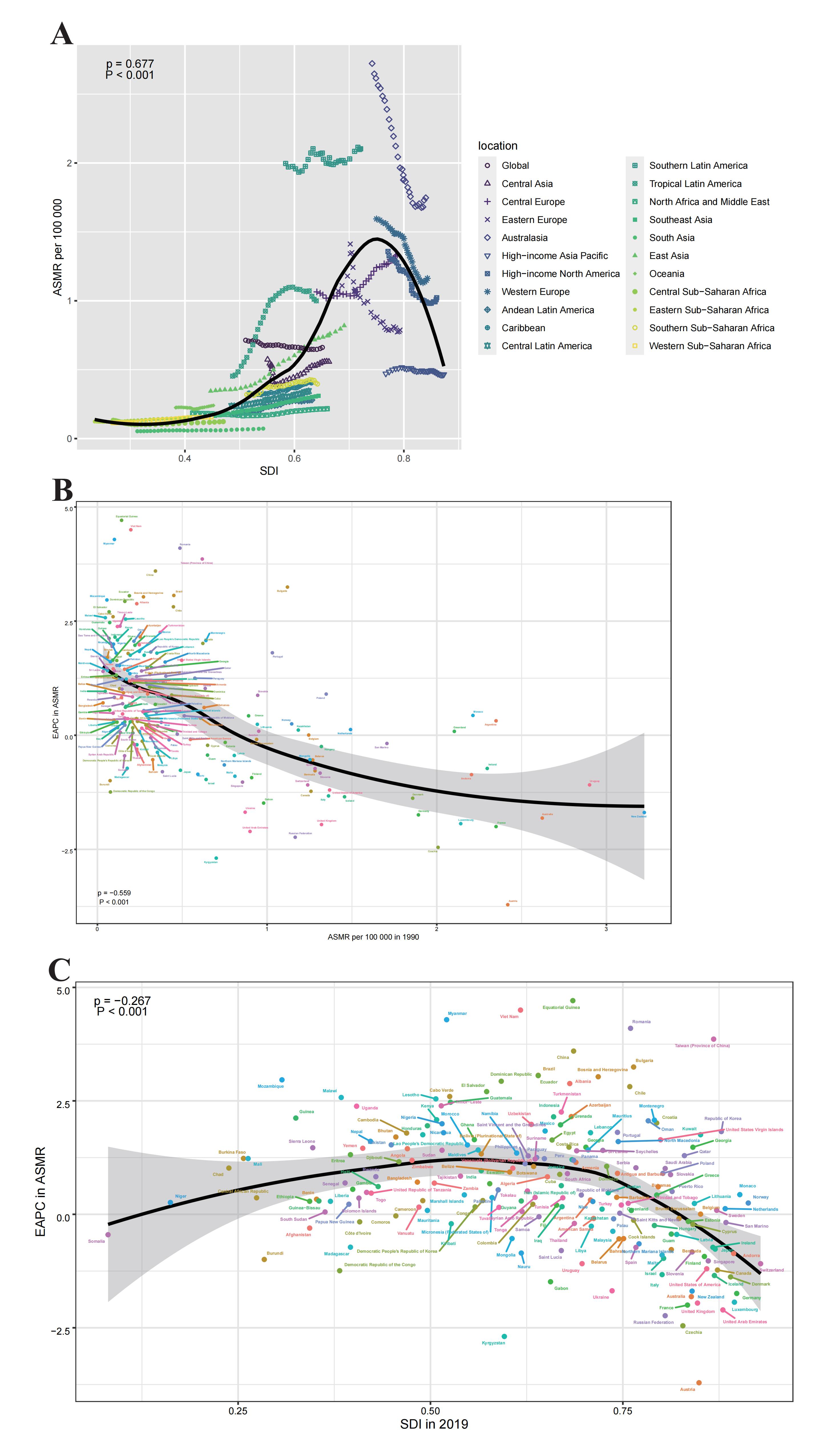

Supplement: Supplementary file 4 [file Image_4.JPEG]

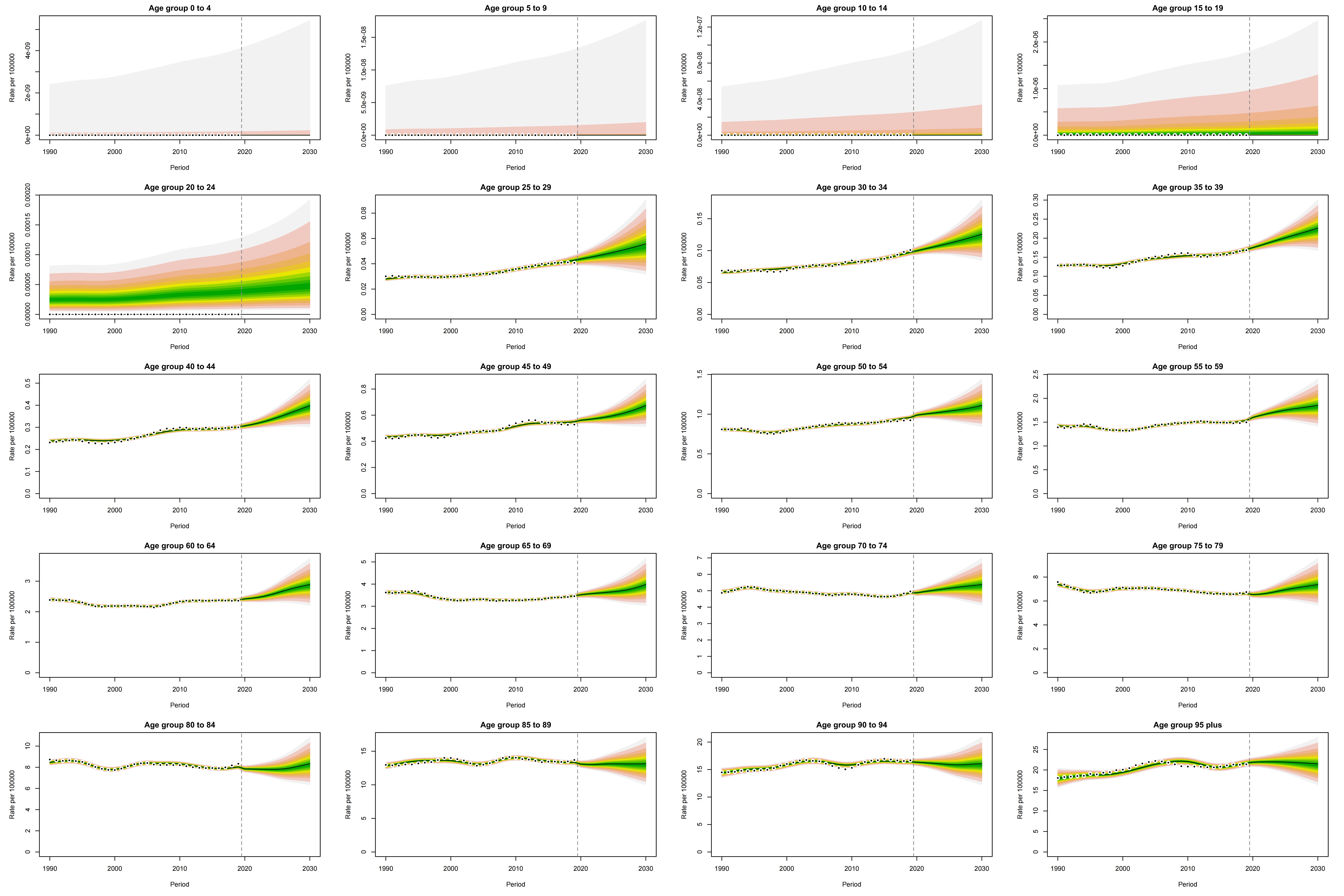

Supplement: Supplementary file 5 [file Image_5.JPEG]

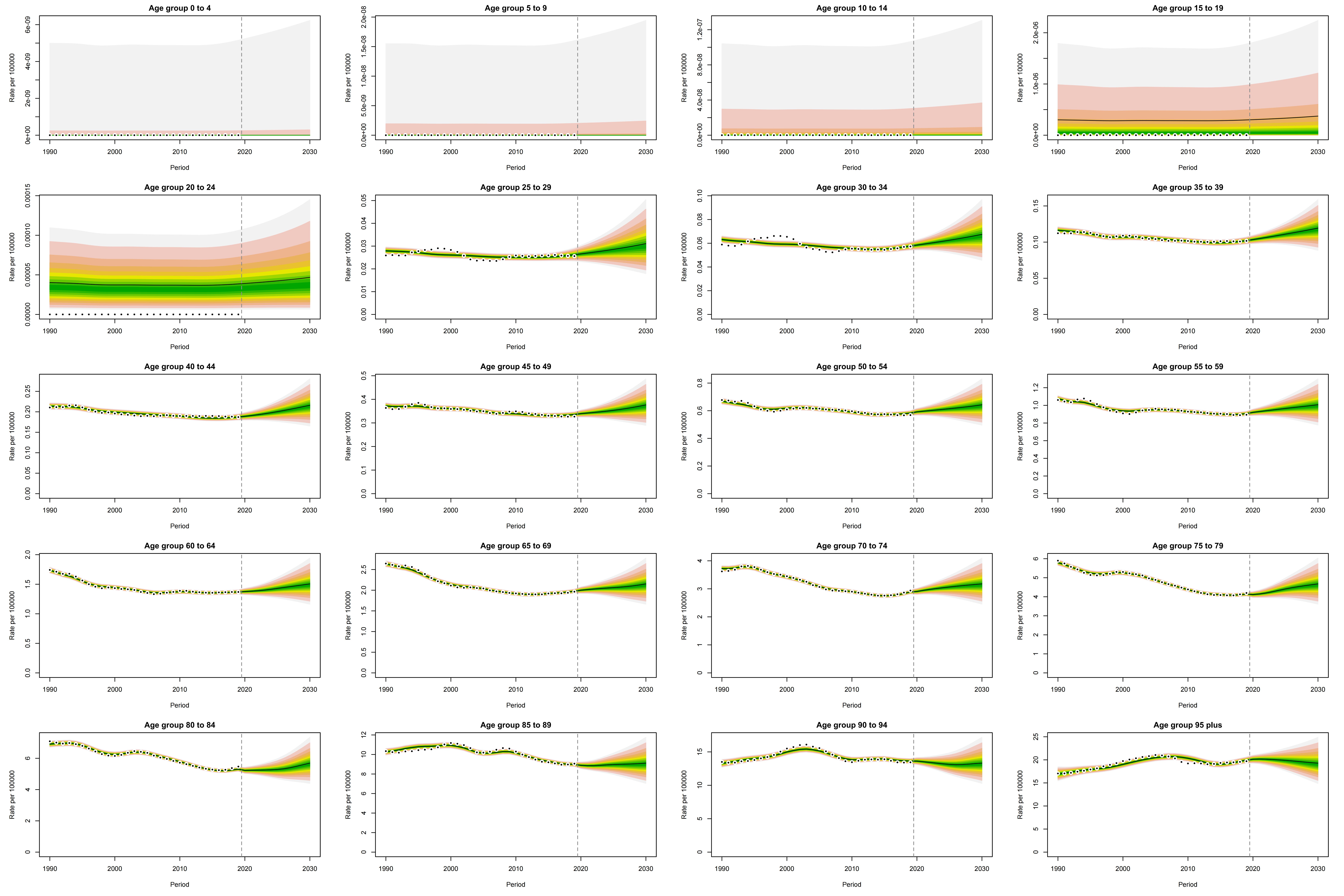

Supplement: Supplementary file 6 [file Image_6.JPEG]

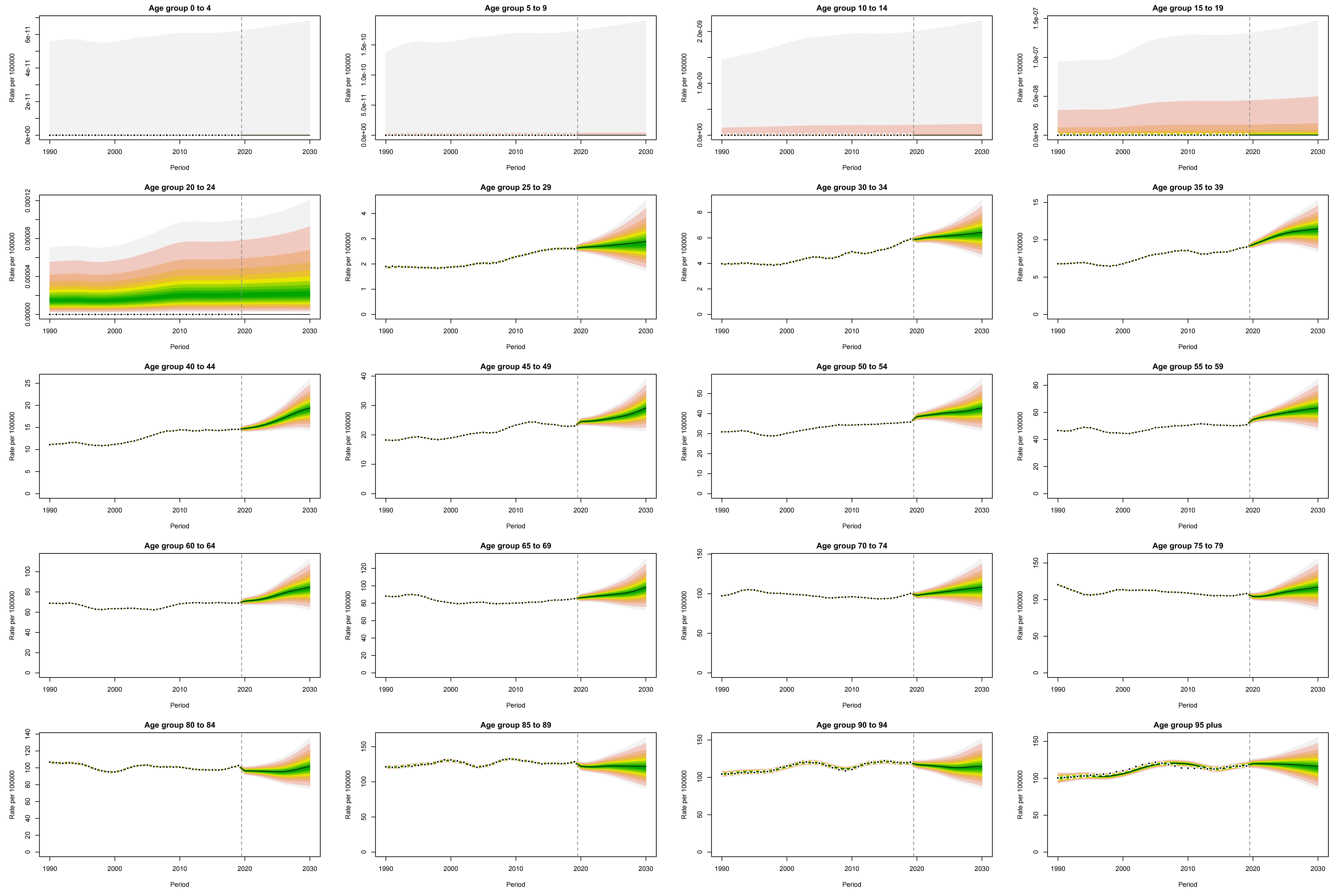

Supplement: Supplementary file 7 [file Image_7.JPEG]

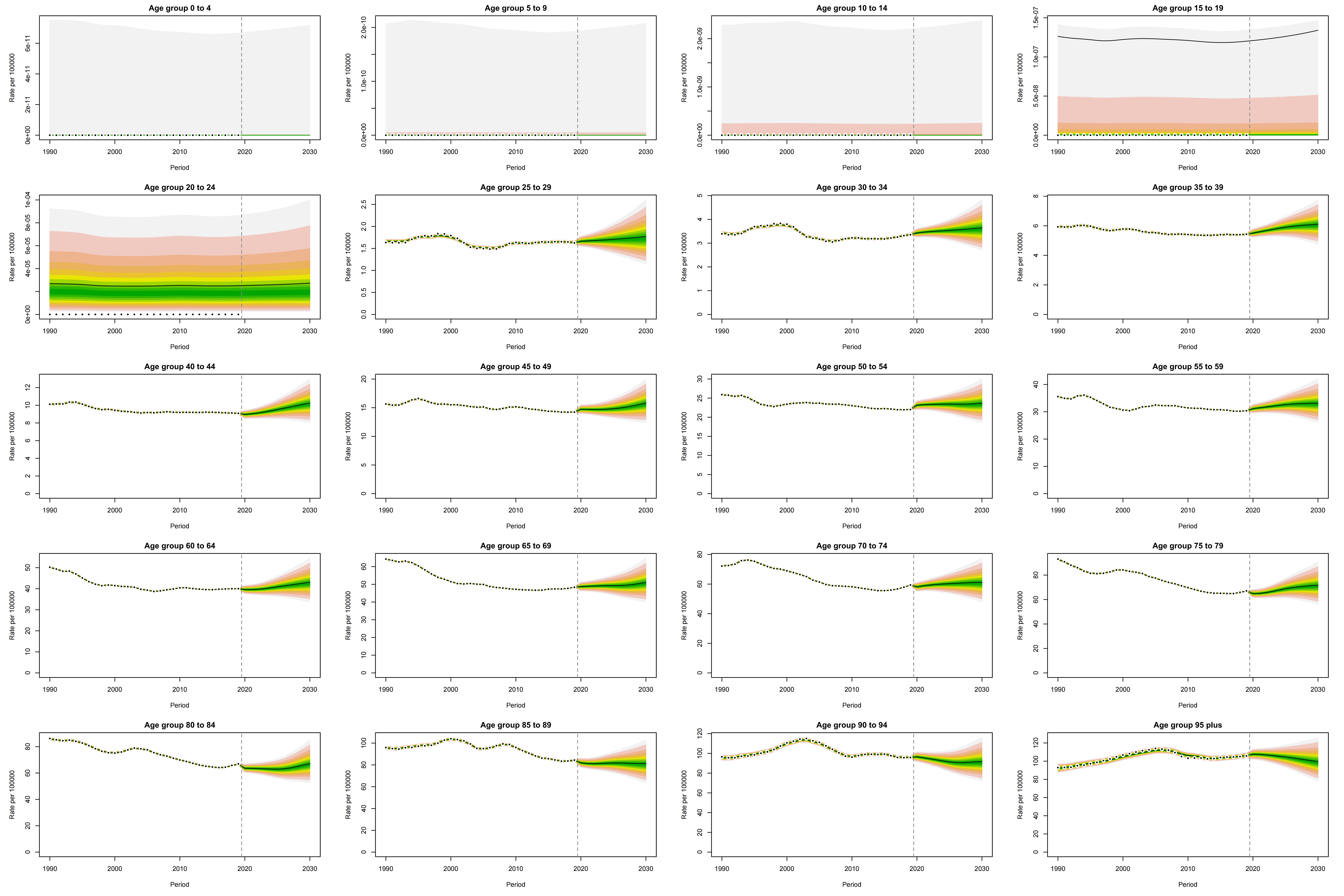

Supplement: Supplementary file 8 [file Image_8.JPEG]
