# Supplementary material for: Global disease burden linked to diet high in red meat and colorectal cancer from 1990 to 2019 and its prediction up to 2030
Source: Front Nutr. 2024 Mar 14;11:1366553. doi: 10.3389/fnut.2024.1366553 (PMC10973012; doi:10.3389/fnut.2024.1366553)
Supplement: Supplementary file 9 [file Table_1.docx]

Table S1. Deaths of colon and rectum cancer attributable to diet high in red meat in 1990 and 2019 for both sexes and 204 countries, with estimated annual percentage change from 1990 to 2019.

| location | SDI  in 1990 | SDI  in 2019 | Deaths cases  in 1990 | ASMR per 100 000 in 1990 | Deaths cases  in 2019 | ASMR per 100 000 in 2019 | EAPC (1990–2019) |
| --- | --- | --- | --- | --- | --- | --- | --- |
| Afghanistan | 0.187 | 20.343 | 16 (1 to 50) | 0.23 (0.02 to 0.72) | 25 (2 to 76) | 0.2 (0.02 to 0.58) | -0.3% (-0.66 to 0.05) |
| Albania | 0.54 | 0.681 | 5 (0 to 12) | 0.23 (0.02 to 0.63) | 18 (3 to 41) | 0.42 (0.07 to 0.98) | 2.88% (2.52 to 3.24) |
| Algeria | 0.436 | 0.652 | 15 (2 to 40) | 0.14 (0.01 to 0.38) | 52 (5 to 145) | 0.17 (0.02 to 0.48) | 0.83% (0.76 to 0.89) |
| American Samoa | 0.606 | 0.712 | 0 (0 to 0) | 0.55 (0.06 to 1.36) | 0 (0 to 1) | 0.52 (0.05 to 1.36) | -0.09% (-0.23 to 0.05) |
| Andorra | 0.834 | 0.894 | 1 (0 to 2) | 2.2 (0.69 to 4.12) | 2 (1 to 5) | 1.72 (0.45 to 3.26) | -0.86% (-0.93 to -0.79) |
| Angola | 0.238 | 0.47 | 6 (1 to 18) | 0.16 (0.02 to 0.47) | 23 (2 to 63) | 0.22 (0.02 to 0.61) | 1.45% (1.22 to 1.67) |
| Antigua and Barbuda | 0.579 | 0.743 | 0 (0 to 0) | 0.3 (0.03 to 0.8) | 0 (0 to 1) | 0.39 (0.04 to 1.08) | 0.9% (0.73 to 1.08) |
| Argentina | 0.581 | 0.708 | 742 (324 to 1129) | 2.35 (1.02 to 3.58) | 1417 (605 to 2160) | 2.61 (1.12 to 3.97) | 0.32% (0.21 to 0.42) |
| Armenia | 0.536 | 0.689 | 10 (1 to 29) | 0.39 (0.03 to 1.06) | 20 (2 to 51) | 0.49 (0.05 to 1.25) | 1.14% (0.86 to 1.43) |
| Australia | 0.738 | 0.839 | 506 (223 to 762) | 2.62 (1.16 to 3.97) | 722 (300 to 1124) | 1.69 (0.72 to 2.61) | -1.81% (-1.99 to -1.64) |
| Austria | 0.753 | 0.849 | 286 (91 to 472) | 2.42 (0.79 to 3.95) | 182 (48 to 329) | 0.97 (0.27 to 1.73) | -3.71% (-3.98 to -3.44) |
| Azerbaijan | 0.576 | 0.683 | 13 (1 to 35) | 0.25 (0.02 to 0.68) | 31 (3 to 86) | 0.35 (0.03 to 0.96) | 2.15% (1.39 to 2.92) |
| Bahamas | 0.692 | 0.796 | 1 (0 to 2) | 0.63 (0.07 to 1.55) | 3 (0 to 6) | 0.67 (0.08 to 1.72) | 0.61% (0.48 to 0.73) |
| Bahrain | 0.553 | 0.751 | 1 (0 to 1) | 0.33 (0.03 to 0.88) | 2 (0 to 6) | 0.29 (0.03 to 0.81) | -0.54% (-0.81 to -0.26) |
| Bangladesh | 0.267 | 0.483 | 15 (4 to 34) | 0.03 (0.01 to 0.08) | 51 (11 to 131) | 0.04 (0.01 to 0.11) | 0.71% (0.57 to 0.84) |
| Barbados | 0.649 | 0.742 | 2 (0 to 5) | 0.61 (0.06 to 1.52) | 3 (0 to 9) | 0.67 (0.06 to 1.87) | 0.37% (0.25 to 0.5) |
| Belarus | 0.591 | 0.745 | 164 (44 to 290) | 1.27 (0.34 to 2.25) | 199 (55 to 382) | 1.25 (0.35 to 2.4) | -0.54% (-1.01 to -0.07) |
| Belgium | 0.746 | 0.851 | 191 (35 to 415) | 1.25 (0.23 to 2.7) | 298 (83 to 540) | 1.25 (0.36 to 2.22) | 0% (-0.23 to 0.24) |
| Belize | 0.428 | 0.603 | 0 (0 to 0) | 0.15 (0.02 to 0.41) | 1 (0 to 2) | 0.21 (0.02 to 0.57) | 0.93% (0.51 to 1.36) |
| Benin | 0.209 | 0.352 | 2 (0 to 5) | 0.1 (0.01 to 0.25) | 5 (1 to 12) | 0.1 (0.02 to 0.26) | 0.33% (0.21 to 0.46) |
| Bermuda | 0.685 | 0.813 | 1 (0 to 2) | 1.28 (0.22 to 2.87) | 1 (0 to 3) | 0.97 (0.18 to 2.07) | -0.78% (-0.91 to -0.65) |
| Bhutan | 0.228 | 0.455 | 0 (0 to 0) | 0.06 (0.01 to 0.16) | 1 (0 to 1) | 0.1 (0.01 to 0.26) | 1.7% (1.66 to 1.75) |
| Bolivia (Plurinational State of) | 0.412 | 0.566 | 9 (1 to 23) | 0.31 (0.03 to 0.79) | 37 (4 to 96) | 0.45 (0.05 to 1.17) | 1.33% (1.24 to 1.43) |
| Bosnia and Herzegovina | 0.533 | 0.718 | 10 (1 to 27) | 0.27 (0.03 to 0.7) | 31 (3 to 89) | 0.53 (0.05 to 1.51) | 3.03% (2.67 to 3.4) |
| Botswana | 0.431 | 0.634 | 2 (0 to 4) | 0.31 (0.02 to 0.84) | 6 (0 to 16) | 0.46 (0.04 to 1.29) | 1.05% (0.82 to 1.29) |
| Brazil | 0.487 | 0.64 | 375 (73 to 814) | 0.45 (0.09 to 0.99) | 2355 (877 to 3841) | 1 (0.37 to 1.64) | 3.06% (2.44 to 3.69) |
| Brunei Darussalam | 0.676 | 0.823 | 1 (0 to 2) | 0.94 (0.08 to 2.51) | 2 (0 to 5) | 0.76 (0.07 to 2.1) | -0.09% (-0.41 to 0.22) |
| Bulgaria | 0.631 | 0.764 | 137 (29 to 275) | 1.12 (0.25 to 2.24) | 241 (62 to 471) | 1.7 (0.47 to 3.32) | 3.25% (2.52 to 3.98) |
| Burkina Faso | 0.125 | 0.257 | 5 (0 to 13) | 0.12 (0.01 to 0.33) | 14 (1 to 39) | 0.17 (0.01 to 0.47) | 1.23% (1.17 to 1.29) |
| Burundi | 0.198 | 0.284 | 2 (0 to 4) | 0.07 (0.02 to 0.17) | 2 (1 to 6) | 0.06 (0.02 to 0.14) | -0.99% (-1.22 to -0.77) |
| Cabo Verde | 0.292 | 0.525 | 0 (0 to 1) | 0.09 (0.01 to 0.24) | 1 (0 to 3) | 0.24 (0.03 to 0.65) | 2.6% (2.21 to 2.99) |
| Cambodia | 0.266 | 0.469 | 8 (1 to 22) | 0.18 (0.02 to 0.5) | 35 (3 to 97) | 0.31 (0.03 to 0.84) | 1.79% (1.76 to 1.82) |
| Cameroon | 0.313 | 0.49 | 8 (1 to 23) | 0.2 (0.02 to 0.57) | 23 (2 to 64) | 0.22 (0.02 to 0.59) | 0.3% (0.25 to 0.36) |
| Canada | 0.79 | 0.873 | 405 (97 to 767) | 1.26 (0.3 to 2.38) | 646 (130 to 1313) | 0.92 (0.19 to 1.84) | -1.23% (-1.35 to -1.1) |
| Central African Republic | 0.186 | 0.274 | 3 (0 to 7) | 0.23 (0.02 to 0.66) | 5 (1 to 13) | 0.24 (0.03 to 0.62) | 0.36% (0.18 to 0.55) |
| Chad | 0.108 | 0.238 | 4 (0 to 10) | 0.13 (0.01 to 0.37) | 9 (1 to 25) | 0.17 (0.01 to 0.48) | 1.02% (0.96 to 1.08) |
| Chile | 0.592 | 0.759 | 41 (4 to 104) | 0.45 (0.05 to 1.12) | 218 (54 to 417) | 0.91 (0.22 to 1.74) | 2.81% (2.67 to 2.96) |
| China | 0.433 | 0.686 | 2722 (392 to 6670) | 0.34 (0.05 to 0.84) | 15719 (3800 to 30286) | 0.81 (0.2 to 1.57) | 3.6% (3.33 to 3.86) |
| Colombia | 0.478 | 0.633 | 48 (4 to 127) | 0.29 (0.02 to 0.76) | 165 (16 to 448) | 0.31 (0.03 to 0.85) | 0.22% (0.12 to 0.32) |
| Comoros | 0.274 | 0.455 | 0 (0 to 1) | 0.12 (0.02 to 0.33) | 1 (0 to 1) | 0.12 (0.02 to 0.31) | -0.03% (-0.17 to 0.1) |
| Congo | 0.364 | 0.568 | 2 (0 to 7) | 0.24 (0.02 to 0.68) | 6 (0 to 18) | 0.25 (0.02 to 0.71) | 0.3% (0.09 to 0.52) |
| Cook Islands | 0.625 | 0.764 | 0 (0 to 0) | 0.26 (0.03 to 0.67) | 0 (0 to 0) | 0.27 (0.03 to 0.68) | -0.13% (-0.35 to 0.09) |
| Costa Rica | 0.532 | 0.68 | 5 (0 to 13) | 0.29 (0.03 to 0.78) | 23 (2 to 63) | 0.44 (0.04 to 1.23) | 1.48% (1.32 to 1.64) |
| Côte d'Ivoire | 0.256 | 0.408 | 7 (1 to 21) | 0.21 (0.02 to 0.59) | 19 (2 to 54) | 0.21 (0.02 to 0.58) | -0.17% (-0.25 to -0.08) |
| Croatia | 0.68 | 0.794 | 38 (3 to 103) | 0.62 (0.05 to 1.68) | 79 (8 to 211) | 0.88 (0.1 to 2.34) | 2.01% (1.65 to 2.37) |
| Cuba | 0.578 | 0.668 | 44 (4 to 121) | 0.44 (0.04 to 1.19) | 102 (9 to 276) | 0.53 (0.04 to 1.43) | 0.88% (0.64 to 1.12) |
| Cyprus | 0.662 | 0.481 | 5 (1 to 10) | 0.65 (0.12 to 1.42) | 11 (2 to 26) | 0.59 (0.09 to 1.39) | -0.22% (-0.5 to 0.06) |
| Czechia | 0.688 | 0.828 | 275 (62 to 544) | 2.01 (0.45 to 3.94) | 230 (41 to 503) | 1.09 (0.21 to 2.34) | -2.46% (-2.62 to -2.29) |
| Democratic People's Republic of Korea | 0.431 | 0.558 | 33 (3 to 89) | 0.21 (0.02 to 0.56) | 65 (7 to 181) | 0.21 (0.02 to 0.57) | 0.16% (0.04 to 0.29) |
| Democratic Republic of the Congo | 0.26 | 0.382 | 11 (2 to 26) | 0.08 (0.01 to 0.19) | 18 (4 to 46) | 0.06 (0.01 to 0.14) | -1.24% (-1.77 to -0.71) |
| Denmark | 0.806 | 0.89 | 150 (42 to 265) | 1.86 (0.53 to 3.22) | 175 (44 to 328) | 1.48 (0.38 to 2.72) | -1.38% (-1.61 to -1.15) |
| Djibouti | 0.275 | 0.459 | 0 (0 to 1) | 0.22 (0.02 to 0.62) | 2 (0 to 5) | 0.29 (0.02 to 0.84) | 1.16% (1.08 to 1.24) |
| Dominica | 0.579 | 0.729 | 0 (0 to 1) | 0.31 (0.03 to 0.84) | 0 (0 to 1) | 0.4 (0.03 to 1.13) | 1.06% (0.97 to 1.14) |
| Dominican Republic | 0.425 | 0.592 | 6 (1 to 15) | 0.16 (0.02 to 0.43) | 28 (2 to 79) | 0.31 (0.03 to 0.86) | 2.93% (2.73 to 3.14) |
| Ecuador | 0.503 | 0.64 | 9 (1 to 26) | 0.19 (0.02 to 0.51) | 54 (6 to 143) | 0.38 (0.04 to 0.99) | 3.06% (2.74 to 3.38) |
| Egypt | 0.403 | 0.658 | 33 (3 to 88) | 0.11 (0.01 to 0.3) | 112 (9 to 330) | 0.18 (0.02 to 0.51) | 1.64% (1.57 to 1.71) |
| El Salvador | 0.39 | 0.573 | 2 (0 to 5) | 0.07 (0.01 to 0.17) | 10 (1 to 26) | 0.16 (0.02 to 0.43) | 2.7% (2.26 to 3.15) |
| Equatorial Guinea | 0.208 | 0.685 | 0 (0 to 1) | 0.14 (0.01 to 0.41) | 2 (0 to 5) | 0.39 (0.03 to 1.13) | 4.71% (4.27 to 5.14) |
| Eritrea | 0.198 | 0.396 | 1 (0 to 3) | 0.11 (0.01 to 0.31) | 5 (0 to 12) | 0.18 (0.02 to 0.48) | 1.31% (0.99 to 1.64) |
| Estonia | 0.665 | 0.835 | 15 (2 to 37) | 0.75 (0.09 to 1.8) | 19 (2 to 49) | 0.7 (0.08 to 1.76) | -0.16% (-0.42 to 0.11) |
| Eswatini | 0.392 | 0.577 | 1 (0 to 2) | 0.34 (0.03 to 0.92) | 2 (0 to 6) | 0.41 (0.03 to 1.14) | 0.67% (0.38 to 0.97) |
| Ethiopia | 0.144 | 0.343 | 23 (3 to 66) | 0.12 (0.01 to 0.33) | 50 (5 to 143) | 0.13 (0.01 to 0.36) | 0.29% (0 to 0.57) |
| Fiji | 0.527 | 0.664 | 1 (0 to 3) | 0.36 (0.04 to 0.9) | 3 (0 to 7) | 0.4 (0.04 to 1.06) | 0.2% (-0.01 to 0.41) |
| Finland | 0.757 | 0.856 | 64 (15 to 125) | 0.91 (0.22 to 1.74) | 87 (18 to 178) | 0.69 (0.15 to 1.41) | -0.93% (-1.22 to -0.63) |
| France | 0.738 | 0.834 | 1982 (719 to 3166) | 2.35 (0.87 to 3.73) | 2092 (651 to 3629) | 1.41 (0.46 to 2.4) | -2% (-2.12 to -1.88) |
| Gabon | 0.388 | 0.656 | 5 (1 to 11) | 0.98 (0.24 to 2.12) | 7 (1 to 15) | 0.69 (0.11 to 1.59) | -1.49% (-1.73 to -1.24) |
| Gambia | 0.218 | 0.399 | 0 (0 to 1) | 0.07 (0.01 to 0.19) | 1 (0 to 2) | 0.09 (0.01 to 0.23) | 0.55% (0.4 to 0.7) |
| Georgia | 0.654 | 0.702 | 20 (2 to 53) | 0.32 (0.03 to 0.87) | 20 (2 to 56) | 0.35 (0.03 to 0.94) | 1.48% (0.81 to 2.16) |
| Germany | 0.819 | 0.898 | 2408 (709 to 4183) | 1.89 (0.57 to 3.23) | 2433 (588 to 4583) | 1.23 (0.33 to 2.26) | -1.74% (-2.12 to -1.37) |
| Ghana | 0.355 | 0.557 | 5 (1 to 13) | 0.09 (0.01 to 0.23) | 21 (2 to 55) | 0.14 (0.02 to 0.37) | 1.65% (1.54 to 1.75) |
| Greece | 0.682 | 0.794 | 132 (30 to 254) | 0.89 (0.21 to 1.7) | 278 (74 to 505) | 1.11 (0.31 to 1.96) | 0.43% (0.07 to 0.79) |
| Greenland | 0.655 | 0.761 | 1 (0 to 1) | 2.1 (0.61 to 3.89) | 2 (0 to 3) | 2.36 (0.69 to 4.36) | 0.08% (-0.16 to 0.33) |
| Grenada | 0.463 | 0.669 | 0 (0 to 0) | 0.22 (0.03 to 0.54) | 0 (0 to 1) | 0.37 (0.04 to 0.97) | 1.97% (1.84 to 2.1) |
| Guam | 0.693 | 0.813 | 0 (0 to 1) | 0.65 (0.08 to 1.58) | 1 (0 to 2) | 0.56 (0.08 to 1.3) | -0.44% (-0.84 to -0.03) |
| Guatemala | 0.315 | 0.526 | 2 (0 to 6) | 0.07 (0.01 to 0.18) | 16 (2 to 43) | 0.15 (0.02 to 0.4) | 2.47% (2.03 to 2.91) |
| Guinea | 0.175 | 0.325 | 2 (0 to 5) | 0.07 (0.01 to 0.17) | 6 (1 to 16) | 0.12 (0.01 to 0.31) | 2.12% (2.01 to 2.24) |
| Guinea-Bissau | 0.2 | 0.355 | 1 (0 to 2) | 0.19 (0.02 to 0.56) | 1 (0 to 4) | 0.21 (0.02 to 0.59) | 0.29% (0.22 to 0.37) |
| Guyana | 0.452 | 0.618 | 1 (0 to 1) | 0.16 (0.03 to 0.38) | 1 (0 to 3) | 0.19 (0.03 to 0.46) | 0.29% (0.1 to 0.49) |
| Haiti | 0.307 | 0.432 | 5 (1 to 14) | 0.16 (0.02 to 0.44) | 12 (1 to 33) | 0.19 (0.02 to 0.51) | 0.61% (0.5 to 0.73) |
| Honduras | 0.33 | 0.496 | 2 (0 to 5) | 0.09 (0.01 to 0.23) | 9 (1 to 25) | 0.15 (0.02 to 0.43) | 1.95% (1.81 to 2.09) |
| Hungary | 0.659 | 0.791 | 190 (30 to 435) | 1.32 (0.21 to 3.02) | 231 (30 to 543) | 1.2 (0.17 to 2.78) | -0.25% (-0.35 to -0.15) |
| Iceland | 0.764 | 0.869 | 4 (2 to 7) | 1.46 (0.53 to 2.29) | 6 (2 to 10) | 1.02 (0.33 to 1.69) | -1.35% (-1.45 to -1.24) |
| India | 0.327 | 0.566 | 182 (58 to 375) | 0.05 (0.01 to 0.1) | 673 (191 to 1464) | 0.06 (0.02 to 0.14) | 0.96% (0.78 to 1.14) |
| Indonesia | 0.452 | 0.66 | 98 (17 to 244) | 0.1 (0.02 to 0.25) | 396 (59 to 1039) | 0.2 (0.03 to 0.52) | 2.15% (2.06 to 2.24) |
| Iran (Islamic Republic of) | 0.404 | 0.67 | 40 (4 to 110) | 0.17 (0.02 to 0.46) | 136 (14 to 356) | 0.2 (0.02 to 0.5) | 0.63% (0.43 to 0.84) |
| Iraq | 0.392 | 0.671 | 9 (1 to 25) | 0.12 (0.01 to 0.33) | 24 (4 to 57) | 0.11 (0.02 to 0.26) | 0.17% (-0.4 to 0.74) |
| Ireland | 0.73 | 0.867 | 92 (31 to 152) | 2.3 (0.77 to 3.77) | 146 (60 to 229) | 1.93 (0.8 to 3) | -0.73% (-0.83 to -0.62) |
| Israel | 0.717 | 0.803 | 30 (2 to 80) | 0.64 (0.05 to 1.72) | 73 (9 to 181) | 0.61 (0.08 to 1.5) | -0.97% (-1.34 to -0.6) |
| Italy | 0.712 | 0.801 | 1199 (340 to 2142) | 1.36 (0.39 to 2.41) | 1522 (404 to 2885) | 1.01 (0.28 to 1.87) | -1.33% (-1.48 to -1.18) |
| Jamaica | 0.542 | 0.684 | 4 (0 to 11) | 0.23 (0.02 to 0.62) | 10 (1 to 28) | 0.34 (0.03 to 0.93) | 1.2% (0.87 to 1.54) |
| Japan | 0.791 | 0.87 | 805 (77 to 2136) | 0.49 (0.05 to 1.3) | 1639 (164 to 4326) | 0.43 (0.04 to 1.11) | -0.77% (-0.86 to -0.67) |
| Jordan | 0.52 | 0.731 | 4 (0 to 10) | 0.28 (0.03 to 0.77) | 17 (2 to 47) | 0.29 (0.03 to 0.8) | 0.48% (0.31 to 0.64) |
| Kazakhstan | 0.602 | 0.723 | 152 (45 to 259) | 1.18 (0.34 to 2.05) | 171 (50 to 299) | 1.02 (0.29 to 1.81) | 0.11% (-0.47 to 0.7) |
| Kenya | 0.333 | 0.508 | 9 (1 to 26) | 0.12 (0.01 to 0.33) | 39 (4 to 107) | 0.19 (0.02 to 0.52) | 2.08% (1.87 to 2.3) |
| Kiribati | 0.425 | 0.527 | 0 (0 to 0) | 0.23 (0.02 to 0.64) | 0 (0 to 0) | 0.23 (0.02 to 0.63) | -0.21% (-0.28 to -0.13) |
| Kuwait | 0.655 | 0.851 | 2 (0 to 4) | 0.28 (0.03 to 0.68) | 9 (1 to 21) | 0.37 (0.04 to 0.94) | 1.75% (1.15 to 2.36) |
| Kyrgyzstan | 0.532 | 0.596 | 21 (5 to 41) | 0.7 (0.16 to 1.35) | 15 (2 to 33) | 0.34 (0.05 to 0.78) | -2.69% (-3.18 to -2.2) |
| Lao People's Democratic Republic | 0.268 | 0.49 | 4 (0 to 12) | 0.19 (0.02 to 0.56) | 14 (1 to 40) | 0.33 (0.03 to 0.94) | 1.75% (1.7 to 1.8) |
| Latvia | 0.675 | 0.82 | 29 (5 to 65) | 0.81 (0.13 to 1.82) | 27 (3 to 68) | 0.67 (0.08 to 1.62) | -0.44% (-0.81 to -0.07) |
| Lebanon | 0.462 | 0.708 | 7 (1 to 20) | 0.35 (0.03 to 0.99) | 26 (2 to 71) | 0.5 (0.04 to 1.37) | 1.8% (1.59 to 2) |
| Lesotho | 0.321 | 0.507 | 1 (0 to 4) | 0.17 (0.01 to 0.48) | 4 (0 to 10) | 0.3 (0.02 to 0.87) | 2.54% (2.38 to 2.71) |
| Liberia | 0.221 | 0.37 | 1 (0 to 3) | 0.1 (0.01 to 0.24) | 2 (0 to 4) | 0.08 (0.01 to 0.23) | 0.29% (-0.23 to 0.8) |
| Libya | 0.405 | 0.709 | 6 (0 to 18) | 0.34 (0.02 to 0.99) | 15 (1 to 41) | 0.29 (0.03 to 0.81) | -0.26% (-0.46 to -0.06) |
| Lithuania | 0.67 | 0.843 | 42 (9 to 85) | 0.94 (0.2 to 1.9) | 53 (10 to 115) | 0.91 (0.18 to 1.92) | 0.24% (-0.06 to 0.54) |
| Luxembourg | 0.815 | 0.895 | 12 (3 to 20) | 2.14 (0.62 to 3.65) | 14 (4 to 24) | 1.36 (0.43 to 2.34) | -1.94% (-2.22 to -1.65) |
| Madagascar | 0.265 | 0.396 | 9 (1 to 24) | 0.18 (0.01 to 0.48) | 15 (1 to 44) | 0.15 (0.01 to 0.41) | -0.72% (-0.86 to -0.59) |
| Malawi | 0.213 | 0.384 | 2 (0 to 4) | 0.05 (0.01 to 0.11) | 6 (1 to 15) | 0.09 (0.01 to 0.22) | 2.57% (2.36 to 2.79) |
| Malaysia | 0.542 | 0.737 | 30 (3 to 82) | 0.35 (0.03 to 0.95) | 97 (10 to 263) | 0.4 (0.04 to 1.09) | -0.37% (-0.65 to -0.1) |
| Maldives | 0.303 | 0.562 | 0 (0 to 0) | 0.06 (0.02 to 0.12) | 0 (0 to 1) | 0.1 (0.02 to 0.22) | 1.42% (1.3 to 1.54) |
| Mali | 0.126 | 0.263 | 7 (1 to 18) | 0.17 (0.01 to 0.47) | 18 (2 to 51) | 0.23 (0.02 to 0.63) | 1.23% (1.14 to 1.31) |
| Malta | 0.666 | 0.801 | 3 (1 to 7) | 0.81 (0.13 to 1.79) | 6 (1 to 14) | 0.66 (0.12 to 1.47) | -0.9% (-1.06 to -0.73) |
| Marshall Islands | 0.398 | 0.544 | 0 (0 to 0) | 0.29 (0.03 to 0.78) | 0 (0 to 0) | 0.33 (0.03 to 0.92) | 0.43% (0.37 to 0.49) |
| Mauritania | 0.308 | 0.496 | 2 (0 to 7) | 0.25 (0.02 to 0.7) | 5 (0 to 13) | 0.24 (0.02 to 0.67) | 0.09% (-0.07 to 0.24) |
| Mauritius | 0.527 | 0.705 | 1 (0 to 3) | 0.18 (0.02 to 0.48) | 5 (0 to 13) | 0.28 (0.03 to 0.79) | 1.42% (1.24 to 1.6) |
| Mexico | 0.507 | 0.649 | 101 (16 to 231) | 0.25 (0.04 to 0.58) | 463 (89 to 1017) | 0.4 (0.07 to 0.89) | 1.84% (1.76 to 1.92) |
| Micronesia (Federated States of) | 0.447 | 0.58 | 0 (0 to 0) | 0.3 (0.03 to 0.85) | 0 (0 to 1) | 0.35 (0.03 to 0.97) | 0.37% (0.33 to 0.4) |
| Monaco | 0.834 | 0.902 | 2 (1 to 3) | 2.21 (0.84 to 3.73) | 3 (1 to 4) | 2.46 (0.99 to 4.11) | 0.43% (0.37 to 0.5) |
| Mongolia | 0.465 | 0.606 | 13 (7 to 19) | 1.25 (0.64 to 1.89) | 27 (13 to 43) | 1.24 (0.6 to 1.96) | -0.53% (-0.75 to -0.32) |
| Montenegro | 0.701 | 0.791 | 4 (1 to 9) | 0.64 (0.1 to 1.48) | 9 (2 to 18) | 0.9 (0.17 to 1.88) | 2.08% (1.75 to 2.4) |
| Morocco | 0.347 | 0.548 | 17 (2 to 47) | 0.13 (0.01 to 0.35) | 60 (5 to 169) | 0.2 (0.02 to 0.56) | 1.53% (1.36 to 1.7) |
| Mozambique | 0.12 | 0.307 | 3 (1 to 7) | 0.06 (0.01 to 0.13) | 12 (2 to 31) | 0.12 (0.02 to 0.3) | 2.96% (2.82 to 3.11) |
| Myanmar | 0.284 | 0.521 | 23 (4 to 57) | 0.1 (0.02 to 0.25) | 124 (11 to 352) | 0.28 (0.03 to 0.78) | 4.29% (4.01 to 4.57) |
| Namibia | 0.454 | 0.612 | 1 (0 to 3) | 0.19 (0.02 to 0.51) | 4 (0 to 10) | 0.27 (0.02 to 0.73) | 1.35% (1.25 to 1.44) |
| Nauru | 0.499 | 0.618 | 0 (0 to 0) | 0.59 (0.06 to 1.62) | 0 (0 to 0) | 0.49 (0.05 to 1.31) | -0.85% (-1.27 to -0.44) |
| Nepal | 0.198 | 0.422 | 7 (1 to 19) | 0.08 (0.01 to 0.21) | 24 (2 to 69) | 0.12 (0.01 to 0.34) | 1.61% (1.35 to 1.87) |
| Netherlands | 0.796 | 0.883 | 297 (69 to 560) | 1.49 (0.36 to 2.79) | 544 (151 to 987) | 1.56 (0.44 to 2.8) | 0.12% (0.01 to 0.24) |
| New Zealand | 0.757 | 0.84 | 125 (52 to 192) | 3.22 (1.34 to 4.93) | 166 (62 to 262) | 2.07 (0.79 to 3.24) | -1.69% (-1.85 to -1.53) |
| Nicaragua | 0.338 | 0.517 | 2 (0 to 4) | 0.11 (0.01 to 0.29) | 7 (1 to 17) | 0.17 (0.02 to 0.43) | 1.8% (1.63 to 1.97) |
| Niger | 0.0728 | 0.162 | 4 (0 to 11) | 0.15 (0.01 to 0.43) | 11 (1 to 30) | 0.16 (0.01 to 0.44) | 0.26% (0.19 to 0.33) |
| Nigeria | 0.305 | 0.515 | 38 (5 to 104) | 0.09 (0.01 to 0.26) | 110 (13 to 295) | 0.14 (0.02 to 0.38) | 2% (1.77 to 2.22) |
| Niue | 0.566 | 0.711 | 0 (0 to 0) | 0.38 (0.04 to 1) | 0 (0 to 0) | 0.41 (0.04 to 1.09) | 0.28% (0.23 to 0.34) |
| North Macedonia | 0.618 | 0.744 | 7 (1 to 20) | 0.4 (0.03 to 1.08) | 18 (2 to 53) | 0.59 (0.05 to 1.69) | 1.6% (1.37 to 1.84) |
| Northern Mariana Islands | 0.692 | 0.771 | 0 (0 to 0) | 0.75 (0.1 to 1.77) | 0 (0 to 1) | 0.63 (0.07 to 1.58) | -0.65% (-0.79 to -0.51) |
| Norway | 0.807 | 0.913 | 79 (16 to 166) | 1.14 (0.24 to 2.38) | 122 (32 to 232) | 1.22 (0.33 to 2.3) | 0.25% (0.04 to 0.45) |
| Oman | 0.441 | 0.783 | 1 (0 to 4) | 0.22 (0.02 to 0.62) | 5 (0 to 12) | 0.32 (0.03 to 0.87) | 2.06% (1.74 to 2.38) |
| Pakistan | 0.247 | 0.449 | 64 (6 to 173) | 0.12 (0.01 to 0.31) | 198 (18 to 551) | 0.19 (0.02 to 0.52) | 1.53% (1.33 to 1.73) |
| Palau | 0.621 | 0.738 | 0 (0 to 0) | 0.42 (0.04 to 1.1) | 0 (0 to 0) | 0.42 (0.04 to 1.1) | -0.1% (-0.17 to -0.03) |
| Palestine | 0.314 | 0.588 | 2 (0 to 6) | 0.27 (0.03 to 0.77) | 7 (1 to 19) | 0.33 (0.04 to 0.85) | 0.54% (0.27 to 0.81) |
| Panama | 0.544 | 0.686 | 4 (0 to 11) | 0.29 (0.03 to 0.74) | 16 (2 to 41) | 0.38 (0.04 to 0.98) | 1.19% (1.04 to 1.34) |
| Papua New Guinea | 0.292 | 0.394 | 3 (0 to 8) | 0.16 (0.01 to 0.47) | 8 (1 to 23) | 0.18 (0.02 to 0.51) | 0.22% (0.13 to 0.32) |
| Paraguay | 0.465 | 0.638 | 12 (4 to 20) | 0.57 (0.19 to 0.95) | 48 (13 to 91) | 0.88 (0.23 to 1.68) | 1.25% (1.07 to 1.44) |
| Peru | 0.501 | 0.648 | 17 (2 to 45) | 0.15 (0.02 to 0.39) | 63 (6 to 175) | 0.19 (0.02 to 0.55) | 1.23% (1.03 to 1.43) |
| Philippines | 0.497 | 0.623 | 85 (8 to 226) | 0.29 (0.03 to 0.77) | 330 (34 to 860) | 0.43 (0.04 to 1.12) | 1.12% (0.91 to 1.33) |
| Poland | 0.632 | 0.802 | 581 (153 to 1087) | 1.35 (0.35 to 2.53) | 1216 (364 to 2265) | 1.72 (0.52 to 3.18) | 0.9% (0.82 to 0.99) |
| Portugal | 0.607 | 0.743 | 136 (21 to 302) | 1.03 (0.17 to 2.27) | 441 (148 to 745) | 1.81 (0.64 to 3) | 1.81% (1.15 to 2.47) |
| Puerto Rico | 0.67 | 0.814 | 12 (1 to 33) | 0.35 (0.03 to 0.92) | 30 (3 to 84) | 0.41 (0.04 to 1.15) | 0.5% (0.26 to 0.75) |
| Qatar | 0.585 | 0.83 | 0 (0 to 1) | 0.4 (0.04 to 1.11) | 3 (0 to 7) | 0.47 (0.04 to 1.28) | 1.29% (0.96 to 1.63) |
| Republic of Korea | 0.686 | 0.878 | 86 (7 to 235) | 0.31 (0.03 to 0.85) | 503 (83 to 1112) | 0.58 (0.1 to 1.27) | 1.82% (1.29 to 2.36) |
| Republic of Moldova | 0.585 | 0.696 | 23 (2 to 62) | 0.51 (0.04 to 1.39) | 26 (2 to 72) | 0.45 (0.04 to 1.23) | 0.43% (-0.28 to 1.14) |
| Romania | 0.625 | 0.76 | 135 (16 to 331) | 0.49 (0.06 to 1.2) | 508 (123 to 968) | 1.4 (0.35 to 2.62) | 4.1% (3.85 to 4.34) |
| Russian Federation | 0.695 | 0.805 | 2102 (615 to 3771) | 1.17 (0.34 to 2.1) | 1906 (341 to 4184) | 0.81 (0.15 to 1.78) | -2.23% (-2.6 to -1.86) |
| Rwanda | 0.257 | 0.429 | 3 (0 to 7) | 0.1 (0.02 to 0.25) | 7 (1 to 19) | 0.13 (0.02 to 0.33) | 0.83% (0.57 to 1.09) |
| Saint Kitts and Nevis | 0.583 | 0.746 | 0 (0 to 0) | 0.47 (0.04 to 1.29) | 0 (0 to 1) | 0.48 (0.04 to 1.32) | 0.02% (-0.14 to 0.19) |
| Saint Lucia | 0.483 | 0.67 | 0 (0 to 1) | 0.38 (0.03 to 1.01) | 1 (0 to 2) | 0.37 (0.03 to 1) | -0.8% (-1.11 to -0.49) |
| Saint Vincent and the Grenadines | 0.462 | 0.627 | 0 (0 to 0) | 0.23 (0.03 to 0.59) | 0 (0 to 1) | 0.33 (0.03 to 0.91) | 1.29% (1.15 to 1.43) |
| Samoa | 0.531 | 0.641 | 0 (0 to 1) | 0.31 (0.03 to 0.86) | 0 (0 to 1) | 0.31 (0.03 to 0.83) | -0.05% (-0.09 to -0.01) |
| San Marino | 0.814 | 0.884 | 1 (0 to 1) | 1.71 (0.49 to 3.12) | 1 (0 to 2) | 1.5 (0.38 to 3.06) | -0.18% (-0.33 to -0.03) |
| Sao Tome and Principe | 0.299 | 0.502 | 0 (0 to 0) | 0.09 (0.03 to 0.18) | 0 (0 to 0) | 0.14 (0.04 to 0.34) | 1.94% (1.81 to 2.08) |
| Saudi Arabia | 0.48 | 0.805 | 8 (1 to 24) | 0.14 (0.01 to 0.43) | 36 (3 to 99) | 0.2 (0.02 to 0.54) | 1.01% (0.87 to 1.15) |
| Senegal | 0.227 | 0.389 | 4 (0 to 12) | 0.15 (0.02 to 0.39) | 12 (1 to 32) | 0.17 (0.02 to 0.46) | 0.69% (0.48 to 0.9) |
| Serbia | 0.626 | 0.767 | 69 (6 to 186) | 0.64 (0.05 to 1.72) | 120 (11 to 329) | 0.77 (0.07 to 2.11) | 1.03% (0.82 to 1.24) |
| Seychelles | 0.567 | 0.724 | 0 (0 to 0) | 0.26 (0.04 to 0.66) | 0 (0 to 1) | 0.44 (0.06 to 1.11) | 1.41% (1.16 to 1.66) |
| Sierra Leone | 0.207 | 0.347 | 1 (0 to 2) | 0.06 (0.01 to 0.13) | 3 (0 to 6) | 0.08 (0.01 to 0.19) | 1.47% (1.21 to 1.73) |
| Singapore | 0.688 | 0.861 | 18 (2 to 44) | 0.86 (0.09 to 2.14) | 52 (9 to 114) | 0.68 (0.12 to 1.52) | -1.03% (-1.12 to -0.93) |
| Slovakia | 0.656 | 0.812 | 56 (7 to 133) | 0.94 (0.12 to 2.23) | 110 (18 to 255) | 1.2 (0.19 to 2.76) | 0.88% (0.64 to 1.12) |
| Slovenia | 0.726 | 0.84 | 32 (7 to 64) | 1.31 (0.3 to 2.64) | 49 (9 to 108) | 1.1 (0.21 to 2.37) | -0.83% (-1.25 to -0.41) |
| Solomon Islands | 0.279 | 0.407 | 0 (0 to 1) | 0.16 (0.02 to 0.48) | 1 (0 to 2) | 0.18 (0.02 to 0.51) | 0.36% (0.16 to 0.55) |
| Somalia | 0.0508 | 0.081 | 4 (0 to 12) | 0.16 (0.01 to 0.47) | 8 (1 to 29) | 0.13 (0.01 to 0.45) | -0.45% (-0.54 to -0.36) |
| South Africa | 0.552 | 0.678 | 70 (9 to 180) | 0.35 (0.04 to 0.92) | 178 (27 to 419) | 0.42 (0.06 to 1) | 0.9% (0.71 to 1.08) |
| South Sudan | 0.248 | 0.363 | 5 (0 to 15) | 0.21 (0.02 to 0.67) | 7 (1 to 22) | 0.21 (0.02 to 0.61) | 0.05% (-0.05 to 0.15) |
| Spain | 0.647 | 0.767 | 687 (173 to 1275) | 1.28 (0.33 to 2.37) | 1214 (275 to 2359) | 1.2 (0.29 to 2.26) | -0.73% (-0.88 to -0.58) |
| Sri Lanka | 0.504 | 0.69 | 3 (1 to 7) | 0.03 (0.01 to 0.07) | 11 (3 to 24) | 0.05 (0.01 to 0.1) | 1.4% (1.29 to 1.51) |
| Sudan | 0.227 | 0.515 | 9 (1 to 25) | 0.1 (0.01 to 0.28) | 26 (3 to 77) | 0.14 (0.01 to 0.42) | 1.41% (1.29 to 1.54) |
| Suriname | 0.498 | 0.636 | 1 (0 to 1) | 0.21 (0.03 to 0.52) | 2 (0 to 4) | 0.28 (0.04 to 0.7) | 1.22% (0.92 to 1.52) |
| Sweden | 0.769 | 0.872 | 143 (29 to 302) | 0.95 (0.2 to 1.96) | 215 (53 to 418) | 1 (0.26 to 1.88) | 0.09% (-0.02 to 0.2) |
| Switzerland | 0.868 | 0.929 | 130 (40 to 217) | 1.24 (0.39 to 2.04) | 171 (51 to 303) | 0.95 (0.29 to 1.64) | -1.09% (-1.19 to -0.99) |
| Syrian Arab Republic | 0.367 | 0.619 | 7 (1 to 20) | 0.13 (0.01 to 0.38) | 16 (1 to 45) | 0.14 (0.01 to 0.4) | 0.06% (-0.15 to 0.28) |
| Taiwan (Province of China) | 0.667 | 0.868 | 95 (13 to 225) | 0.62 (0.08 to 1.49) | 713 (189 to 1384) | 1.81 (0.48 to 3.52) | 3.86% (3.49 to 4.24) |
| Tajikistan | 0.468 | 0.539 | 6 (1 to 17) | 0.21 (0.02 to 0.57) | 9 (1 to 25) | 0.21 (0.02 to 0.56) | 0.84% (0.13 to 1.56) |
| Thailand | 0.508 | 0.687 | 74 (8 to 197) | 0.22 (0.02 to 0.57) | 242 (22 to 697) | 0.24 (0.02 to 0.69) | -0.2% (-0.46 to 0.06) |
| Timor-Leste | 0.274 | 0.514 | 0 (0 to 1) | 0.13 (0.01 to 0.36) | 2 (0 to 5) | 0.24 (0.02 to 0.67) | 2.39% (2.11 to 2.67) |
| Togo | 0.266 | 0.417 | 1 (0 to 3) | 0.09 (0.01 to 0.23) | 3 (0 to 9) | 0.1 (0.02 to 0.27) | 0.48% (0.39 to 0.58) |
| Tokelau | 0.427 | 0.626 | 0 (0 to 0) | 0.27 (0.02 to 0.76) | 0 (0 to 0) | 0.31 (0.03 to 0.84) | 0.51% (0.45 to 0.57) |
| Tonga | 0.51 | 0.636 | 0 (0 to 0) | 0.16 (0.01 to 0.43) | 0 (0 to 0) | 0.19 (0.02 to 0.52) | 0.37% (0.24 to 0.5) |
| Trinidad and Tobago | 0.618 | 0.757 | 2 (0 to 6) | 0.3 (0.03 to 0.79) | 6 (1 to 16) | 0.31 (0.03 to 0.88) | 0.24% (0.06 to 0.42) |
| Tunisia | 0.434 | 0.672 | 8 (1 to 23) | 0.18 (0.02 to 0.49) | 25 (2 to 72) | 0.21 (0.02 to 0.6) | 0.36% (0.28 to 0.45) |
| Turkey | 0.473 | 0.748 | 107 (9 to 297) | 0.31 (0.03 to 0.85) | 256 (23 to 711) | 0.3 (0.03 to 0.82) | 0.21% (-0.38 to 0.79) |
| Turkmenistan | 0.548 | 0.67 | 8 (1 to 16) | 0.37 (0.07 to 0.78) | 22 (7 to 40) | 0.59 (0.18 to 1.06) | 2.25% (1.47 to 3.04) |
| Tuvalu | 0.426 | 0.589 | 0 (0 to 0) | 0.26 (0.02 to 0.71) | 0 (0 to 0) | 0.29 (0.02 to 0.82) | 0.15% (0.09 to 0.22) |
| Uganda | 0.167 | 0.404 | 7 (1 to 19) | 0.12 (0.01 to 0.3) | 31 (3 to 86) | 0.23 (0.02 to 0.62) | 2.38% (2.14 to 2.62) |
| Ukraine | 0.653 | 0.736 | 620 (81 to 1464) | 0.87 (0.12 to 2.06) | 477 (46 to 1302) | 0.63 (0.06 to 1.72) | -1.69% (-2.17 to -1.2) |
| United Arab Emirates | 0.621 | 0.88 | 3 (1 to 7) | 0.9 (0.17 to 2.18) | 14 (1 to 40) | 0.5 (0.04 to 1.49) | -2.11% (-2.32 to -1.89) |
| United Kingdom | 0.745 | 0.847 | 1193 (297 to 2369) | 1.32 (0.33 to 2.6) | 1059 (221 to 2248) | 0.81 (0.18 to 1.7) | -1.96% (-2.22 to -1.69) |
| United Republic of Tanzania | 0.26 | 0.423 | 15 (2 to 40) | 0.15 (0.02 to 0.39) | 39 (4 to 103) | 0.17 (0.02 to 0.44) | 0.47% (0.25 to 0.69) |
| United States of America | 0.768 | 0.859 | 4392 (1231 to 7939) | 1.37 (0.39 to 2.45) | 5738 (1601 to 10372) | 1.03 (0.29 to 1.84) | -1.2% (-1.32 to -1.08) |
| United States Virgin Islands | 0.667 | 0.799 | 0 (0 to 1) | 0.5 (0.05 to 1.36) | 1 (0 to 4) | 0.72 (0.07 to 1.94) | 1.64% (1.37 to 1.91) |
| Uruguay | 0.581 | 0.697 | 113 (45 to 175) | 2.9 (1.16 to 4.49) | 133 (44 to 225) | 2.34 (0.78 to 3.89) | -1.09% (-1.25 to -0.92) |
| Uzbekistan | 0.49 | 0.631 | 29 (3 to 71) | 0.25 (0.02 to 0.61) | 73 (12 to 169) | 0.41 (0.06 to 0.98) | 1.98% (1.71 to 2.25) |
| Vanuatu | 0.361 | 0.485 | 0 (0 to 0) | 0.29 (0.03 to 0.73) | 1 (0 to 1) | 0.32 (0.03 to 0.87) | 0.16% (-0.03 to 0.35) |
| Venezuela (Bolivarian Republic of) | 0.509 | 0.607 | 23 (2 to 63) | 0.25 (0.02 to 0.68) | 99 (9 to 277) | 0.35 (0.03 to 0.96) | 1.02% (0.9 to 1.13) |
| Viet Nam | 0.39 | 0.617 | 77 (7 to 209) | 0.2 (0.02 to 0.54) | 564 (60 to 1418) | 0.61 (0.06 to 1.54) | 4.5% (4.27 to 4.73) |
| Yemen | 0.176 | 0.412 | 4 (0 to 12) | 0.09 (0.01 to 0.26) | 15 (2 to 42) | 0.12 (0.01 to 0.32) | 1.46% (1.24 to 1.67) |
| Zambia | 0.299 | 0.505 | 5 (1 to 15) | 0.2 (0.02 to 0.56) | 16 (1 to 46) | 0.25 (0.02 to 0.7) | 0.69% (0.61 to 0.77) |
| Zimbabwe | 0.394 | 0.476 | 7 (1 to 18) | 0.19 (0.02 to 0.49) | 17 (2 to 47) | 0.26 (0.03 to 0.72) | 1.19% (0.9 to 1.48) |
