# Supplementary material for: Global disease burden linked to diet high in red meat and colorectal cancer from 1990 to 2019 and its prediction up to 2030
Source: Front Nutr. 2024 Mar 14;11:1366553. doi: 10.3389/fnut.2024.1366553 (PMC10973012; doi:10.3389/fnut.2024.1366553)
Supplement: Supplementary file 10 [file Table_2.docx]

Table S2. DALYs of colon and rectum cancer attributable to diet high in red meat in 1990 and 2019 for both sexes and 204 countries, with estimated annual percentage change from 1990 to 2019.

| location | SDI  in 1990 | SDI  in 2019 | Deaths cases  in 1990 | ASMR per 100 000 in 1990 | Deaths cases  in 2019 | ASMR per 100 000 in 2019 | EAPC (1990–2019) |
| --- | --- | --- | --- | --- | --- | --- | --- |
| Afghanistan | 0.187 | 20.343 | 462 (29 to 1446) | 6.17 (0.4 to 19.14) | 815 (60 to 2450) | 5.1 (0.4 to 15.33) | -0.46% (-0.8 to -0.12) |
| Albania | 0.54 | 0.681 | 120 (10 to 329) | 5.38 (0.43 to 14.67) | 410 (76 to 908) | 10.36 (1.96 to 22.7) | 3.19% (2.8 to 3.59) |
| Algeria | 0.436 | 0.652 | 415 (40 to 1134) | 3.21 (0.32 to 8.67) | 1310 (115 to 3657) | 3.71 (0.33 to 10.34) | 0.58% (0.52 to 0.63) |
| American Samoa | 0.606 | 0.712 | 3 (0 to 8) | 12.8 (1.55 to 31.85) | 6 (1 to 15) | 12.08 (1.31 to 31.2) | -0.11% (-0.25 to 0.04) |
| Andorra | 0.834 | 0.894 | 27 (9 to 50) | 48.01 (16.2 to 87.97) | 52 (15 to 96) | 37.66 (10.75 to 69.04) | -0.87% (-0.93 to -0.8) |
| Angola | 0.238 | 0.47 | 177 (16 to 545) | 3.93 (0.36 to 11.86) | 665 (54 to 1870) | 5.17 (0.44 to 14.37) | 1.28% (1.04 to 1.52) |
| Antigua and Barbuda | 0.579 | 0.743 | 3 (0 to 9) | 6.61 (0.56 to 18.17) | 8 (1 to 23) | 8.07 (0.7 to 22.47) | 0.65% (0.48 to 0.82) |
| Argentina | 0.581 | 0.708 | 17162 (7884 to 25597) | 52.83 (24.35 to 78.88) | 30869 (13738 to 46440) | 58.62 (26.32 to 88.11) | 0.34% (0.26 to 0.42) |
| Armenia | 0.536 | 0.689 | 301 (24 to 817) | 10.28 (0.82 to 27.94) | 476 (54 to 1191) | 11.63 (1.32 to 29.09) | 0.65% (0.35 to 0.94) |
| Australia | 0.738 | 0.839 | 11793 (5364 to 17675) | 61.34 (28.08 to 91.86) | 14890 (6634 to 22850) | 38.44 (17.16 to 58.77) | -1.89% (-2.07 to -1.72) |
| Austria | 0.753 | 0.849 | 6316 (2151 to 10174) | 57.07 (19.89 to 91.06) | 3491 (979 to 6153) | 21.2 (6.17 to 36.75) | -4.05% (-4.35 to -3.75) |
| Azerbaijan | 0.576 | 0.683 | 409 (35 to 1122) | 7.3 (0.64 to 19.97) | 927 (77 to 2520) | 8.75 (0.73 to 24.02) | 1.47% (0.69 to 2.24) |
| Bahamas | 0.692 | 0.796 | 27 (3 to 64) | 15.78 (1.93 to 38.64) | 68 (9 to 168) | 16.56 (2.16 to 41.02) | 0.55% (0.43 to 0.67) |
| Bahrain | 0.553 | 0.751 | 16 (1 to 42) | 7.26 (0.63 to 19.75) | 70 (6 to 198) | 5.97 (0.51 to 16.39) | -1.02% (-1.26 to -0.77) |
| Bangladesh | 0.267 | 0.483 | 403 (100 to 946) | 0.79 (0.2 to 1.87) | 1264 (256 to 3274) | 0.94 (0.19 to 2.41) | 0.53% (0.43 to 0.63) |
| Barbados | 0.649 | 0.742 | 38 (4 to 95) | 14.07 (1.63 to 34.57) | 69 (6 to 192) | 14.5 (1.25 to 40.06) | 0.14% (0.04 to 0.23) |
| Belarus | 0.591 | 0.745 | 4419 (1220 to 7575) | 34.33 (9.53 to 58.82) | 4791 (1384 to 8943) | 31.14 (9.24 to 57.71) | -0.89% (-1.36 to -0.43) |
| Belgium | 0.746 | 0.851 | 3816 (744 to 8256) | 26.04 (5.16 to 55.44) | 5700 (1673 to 9897) | 27.45 (8.33 to 46.94) | 0.19% (-0.13 to 0.5) |
| Belize | 0.428 | 0.603 | 3 (0 to 9) | 3.41 (0.32 to 9.21) | 15 (2 to 41) | 5.05 (0.51 to 13.55) | 1.08% (0.66 to 1.5) |
| Benin | 0.209 | 0.352 | 48 (6 to 121) | 2.26 (0.29 to 5.79) | 120 (16 to 312) | 2.28 (0.31 to 5.87) | 0.1% (-0.03 to 0.23) |
| Bermuda | 0.685 | 0.813 | 19 (4 to 39) | 29.05 (5.57 to 62.04) | 26 (6 to 54) | 21.9 (4.7 to 44.13) | -0.76% (-0.92 to -0.61) |
| Bhutan | 0.228 | 0.455 | 4 (0 to 11) | 1.41 (0.18 to 3.92) | 12 (1 to 33) | 2.1 (0.26 to 5.67) | 1.31% (1.24 to 1.38) |
| Bolivia (Plurinational State of) | 0.412 | 0.566 | 251 (28 to 609) | 7.24 (0.75 to 17.89) | 914 (109 to 2239) | 9.99 (1.13 to 24.72) | 1.12% (1.02 to 1.22) |
| Bosnia and Herzegovina | 0.533 | 0.718 | 282 (31 to 729) | 6.5 (0.73 to 16.78) | 693 (58 to 1993) | 12.04 (1.01 to 34.63) | 2.78% (2.44 to 3.12) |
| Botswana | 0.431 | 0.634 | 43 (3 to 121) | 7.1 (0.55 to 19.5) | 163 (13 to 446) | 10.61 (0.86 to 29.29) | 0.98% (0.68 to 1.27) |
| Brazil | 0.487 | 0.64 | 10251 (2074 to 21556) | 10.57 (2.1 to 22.58) | 59953 (23665 to 95035) | 24.7 (9.68 to 39.29) | 3.19% (2.52 to 3.86) |
| Brunei Darussalam | 0.676 | 0.823 | 24 (2 to 62) | 21.03 (1.87 to 55.59) | 53 (5 to 146) | 16.17 (1.39 to 44.25) | -0.37% (-0.74 to 0) |
| Bulgaria | 0.631 | 0.764 | 3662 (824 to 7067) | 29.85 (6.95 to 56.9) | 5620 (1650 to 10809) | 43.56 (13.22 to 82.8) | 3.09% (2.35 to 3.83) |
| Burkina Faso | 0.125 | 0.257 | 125 (11 to 348) | 2.74 (0.25 to 7.51) | 375 (31 to 1077) | 3.78 (0.31 to 10.77) | 1.1% (1.01 to 1.2) |
| Burundi | 0.198 | 0.284 | 48 (10 to 114) | 1.87 (0.42 to 4.46) | 73 (17 to 178) | 1.39 (0.35 to 3.36) | -1.22% (-1.47 to -0.96) |
| Cabo Verde | 0.292 | 0.525 | 5 (0 to 13) | 2.18 (0.22 to 5.91) | 21 (2 to 57) | 4.76 (0.48 to 13.04) | 2.08% (1.77 to 2.4) |
| Cambodia | 0.266 | 0.469 | 238 (22 to 672) | 4.66 (0.44 to 12.98) | 952 (84 to 2730) | 7.36 (0.66 to 20.83) | 1.54% (1.51 to 1.56) |
| Cameroon | 0.313 | 0.49 | 225 (19 to 627) | 4.68 (0.41 to 13.09) | 627 (55 to 1753) | 4.78 (0.44 to 13.29) | 0.15% (0.11 to 0.19) |
| Canada | 0.79 | 0.873 | 9713 (2488 to 17483) | 30.55 (7.84 to 54.67) | 13436 (3035 to 26631) | 21.14 (4.98 to 41.47) | -1.44% (-1.59 to -1.3) |
| Central African Republic | 0.186 | 0.274 | 78 (6 to 216) | 5.82 (0.44 to 16.28) | 165 (21 to 419) | 6.22 (0.74 to 16.07) | 0.47% (0.25 to 0.68) |
| Chad | 0.108 | 0.238 | 89 (7 to 251) | 3.03 (0.24 to 8.59) | 232 (18 to 664) | 3.83 (0.31 to 10.91) | 0.88% (0.82 to 0.94) |
| Chile | 0.592 | 0.759 | 965 (104 to 2390) | 9.49 (1.02 to 23.53) | 4649 (1206 to 8749) | 19.61 (5.04 to 36.71) | 2.92% (2.79 to 3.06) |
| China | 0.433 | 0.686 | 79255 (11969 to 192441) | 8.53 (1.24 to 20.76) | 417044 (109794 to 777994) | 20.57 (5.37 to 38.34) | 3.73% (3.45 to 4.02) |
| Colombia | 0.478 | 0.633 | 1256 (111 to 3272) | 6.58 (0.56 to 17.33) | 3937 (403 to 10509) | 7.49 (0.77 to 19.98) | 0.43% (0.32 to 0.55) |
| Comoros | 0.274 | 0.455 | 7 (1 to 19) | 2.86 (0.32 to 7.99) | 15 (2 to 40) | 2.9 (0.38 to 7.65) | -0.1% (-0.28 to 0.09) |
| Congo | 0.364 | 0.568 | 69 (6 to 205) | 5.86 (0.51 to 17.06) | 178 (14 to 527) | 5.93 (0.48 to 17.21) | 0.09% (-0.13 to 0.32) |
| Cook Islands | 0.625 | 0.764 | 1 (0 to 2) | 6.18 (0.64 to 15.44) | 1 (0 to 4) | 6.14 (0.72 to 15.35) | -0.16% (-0.42 to 0.11) |
| Costa Rica | 0.532 | 0.68 | 124 (11 to 325) | 6.71 (0.58 to 17.63) | 521 (45 to 1457) | 10.03 (0.87 to 28.08) | 1.53% (1.32 to 1.74) |
| Côte d'Ivoire | 0.256 | 0.408 | 220 (16 to 626) | 4.78 (0.38 to 13.68) | 538 (42 to 1550) | 4.54 (0.37 to 12.86) | -0.39% (-0.5 to -0.29) |
| Croatia | 0.68 | 0.794 | 886 (73 to 2411) | 13.87 (1.15 to 37.78) | 1605 (182 to 4178) | 19.6 (2.29 to 50.1) | 1.92% (1.54 to 2.29) |
| Cuba | 0.578 | 0.668 | 1013 (81 to 2736) | 9.84 (0.79 to 26.56) | 2104 (184 to 5618) | 11.41 (1.01 to 30.46) | 0.76% (0.47 to 1.04) |
| Cyprus | 0.662 | 0.481 | 112 (22 to 236) | 13.98 (2.76 to 29.11) | 225 (34 to 521) | 11.95 (1.86 to 27.42) | -0.48% (-0.82 to -0.14) |
| Czechia | 0.688 | 0.828 | 6579 (1590 to 12521) | 48.94 (12.01 to 92.17) | 4818 (981 to 10320) | 24.55 (4.94 to 51.91) | -2.77% (-2.94 to -2.6) |
| Democratic People's Republic of Korea | 0.431 | 0.558 | 1014 (96 to 2801) | 5.61 (0.55 to 15.22) | 1802 (176 to 5051) | 5.47 (0.54 to 15.3) | 0.13% (0 to 0.26) |
| Democratic Republic of the Congo | 0.26 | 0.382 | 313 (55 to 762) | 1.8 (0.32 to 4.33) | 524 (107 to 1307) | 1.31 (0.28 to 3.25) | -1.25% (-1.79 to -0.71) |
| Denmark | 0.806 | 0.89 | 3269 (989 to 5547) | 43.66 (13.54 to 73.12) | 3419 (885 to 6152) | 32.03 (8.6 to 56.55) | -1.67% (-1.9 to -1.44) |
| Djibouti | 0.275 | 0.459 | 9 (1 to 26) | 5.17 (0.4 to 15.04) | 48 (4 to 145) | 6.85 (0.52 to 20.43) | 1.11% (1.03 to 1.19) |
| Dominica | 0.579 | 0.729 | 5 (0 to 13) | 6.87 (0.55 to 19) | 8 (1 to 22) | 8.74 (0.73 to 24.47) | 0.89% (0.81 to 0.97) |
| Dominican Republic | 0.425 | 0.592 | 147 (14 to 397) | 3.64 (0.35 to 9.8) | 693 (56 to 1957) | 7.19 (0.58 to 20.25) | 2.92% (2.75 to 3.08) |
| Ecuador | 0.503 | 0.64 | 229 (19 to 625) | 4.06 (0.34 to 11.06) | 1359 (165 to 3443) | 8.72 (1.03 to 22.27) | 3.22% (2.93 to 3.52) |
| Egypt | 0.403 | 0.658 | 996 (96 to 2678) | 2.96 (0.29 to 7.92) | 3328 (262 to 9828) | 4.56 (0.37 to 13.4) | 1.57% (1.48 to 1.65) |
| El Salvador | 0.39 | 0.573 | 54 (8 to 130) | 1.7 (0.25 to 4.11) | 229 (25 to 620) | 3.89 (0.43 to 10.52) | 2.65% (2.19 to 3.11) |
| Equatorial Guinea | 0.208 | 0.685 | 8 (1 to 23) | 3.54 (0.29 to 10.33) | 48 (4 to 140) | 8.82 (0.71 to 24.93) | 4.31% (3.9 to 4.72) |
| Eritrea | 0.198 | 0.396 | 36 (4 to 102) | 2.9 (0.31 to 8.33) | 143 (15 to 385) | 4.41 (0.48 to 11.88) | 1.17% (0.87 to 1.48) |
| Estonia | 0.665 | 0.835 | 377 (48 to 883) | 18.6 (2.44 to 43.41) | 374 (45 to 932) | 15.37 (1.95 to 37.8) | -0.74% (-1.02 to -0.47) |
| Eswatini | 0.392 | 0.577 | 25 (3 to 66) | 7.87 (0.76 to 20.64) | 61 (5 to 170) | 9.52 (0.76 to 26.3) | 0.76% (0.39 to 1.12) |
| Ethiopia | 0.144 | 0.343 | 717 (79 to 2119) | 3.05 (0.34 to 8.85) | 1430 (146 to 4062) | 3.02 (0.32 to 8.56) | -0.1% (-0.4 to 0.2) |
| Fiji | 0.527 | 0.664 | 38 (5 to 91) | 8.73 (1.1 to 21.49) | 73 (8 to 191) | 9.2 (1.02 to 24.13) | -0.06% (-0.3 to 0.19) |
| Finland | 0.757 | 0.856 | 1458 (375 to 2667) | 21.29 (5.6 to 38.36) | 1694 (371 to 3337) | 15.75 (3.69 to 30.19) | -1% (-1.3 to -0.7) |
| France | 0.738 | 0.834 | 40268 (15975 to 62585) | 51.07 (20.78 to 79.03) | 37724 (12328 to 62947) | 30.73 (10.26 to 50.29) | -1.95% (-2.05 to -1.86) |
| Gabon | 0.388 | 0.656 | 151 (39 to 313) | 25.26 (6.45 to 53.27) | 190 (32 to 424) | 16.33 (2.59 to 36.5) | -1.8% (-2.05 to -1.54) |
| Gambia | 0.218 | 0.399 | 6 (1 to 17) | 1.66 (0.21 to 4.34) | 20 (3 to 54) | 1.97 (0.27 to 5.16) | 0.46% (0.27 to 0.65) |
| Georgia | 0.654 | 0.702 | 552 (44 to 1511) | 8.83 (0.71 to 24.05) | 491 (41 to 1342) | 8.95 (0.74 to 24.4) | 1.14% (0.47 to 1.8) |
| Germany | 0.819 | 0.898 | 52539 (16331 to 88169) | 43.52 (13.87 to 72.27) | 45750 (11790 to 82596) | 26.97 (7.41 to 47.3) | -1.89% (-2.12 to -1.66) |
| Ghana | 0.355 | 0.557 | 149 (19 to 389) | 2.13 (0.28 to 5.47) | 568 (63 to 1542) | 3.18 (0.37 to 8.46) | 1.55% (1.43 to 1.67) |
| Greece | 0.682 | 0.794 | 2820 (667 to 5237) | 19.18 (4.64 to 35.19) | 5133 (1517 to 8928) | 24.62 (7.8 to 41.74) | 0.67% (0.39 to 0.96) |
| Greenland | 0.655 | 0.761 | 21 (7 to 37) | 52.4 (16.19 to 93.77) | 40 (12 to 71) | 55.26 (16.95 to 98.79) | -0.06% (-0.27 to 0.15) |
| Grenada | 0.463 | 0.669 | 4 (1 to 9) | 5.15 (0.72 to 12.81) | 10 (1 to 25) | 8.37 (0.92 to 22) | 1.87% (1.77 to 1.96) |
| Guam | 0.693 | 0.813 | 13 (2 to 29) | 14.61 (2.16 to 34.01) | 27 (4 to 62) | 14.45 (2.2 to 32.48) | 0.08% (-0.29 to 0.45) |
| Guatemala | 0.315 | 0.526 | 68 (10 to 169) | 1.68 (0.25 to 4.18) | 419 (48 to 1143) | 3.5 (0.41 to 9.47) | 2.6% (2.18 to 3.01) |
| Guinea | 0.175 | 0.325 | 54 (9 to 132) | 1.56 (0.26 to 3.85) | 158 (17 to 428) | 2.68 (0.3 to 7.24) | 2.19% (2.07 to 2.31) |
| Guinea-Bissau | 0.2 | 0.355 | 21 (2 to 62) | 4.77 (0.39 to 13.82) | 41 (3 to 116) | 4.91 (0.41 to 13.85) | 0.15% (0.07 to 0.22) |
| Guyana | 0.452 | 0.618 | 16 (2 to 39) | 3.84 (0.62 to 9.23) | 30 (5 to 76) | 4.49 (0.69 to 11.23) | 0.39% (0.19 to 0.6) |
| Haiti | 0.307 | 0.432 | 144 (16 to 401) | 4.03 (0.45 to 11.09) | 336 (34 to 937) | 4.35 (0.44 to 11.94) | 0.49% (0.38 to 0.61) |
| Honduras | 0.33 | 0.496 | 51 (5 to 137) | 2.21 (0.24 to 5.89) | 219 (20 to 623) | 3.45 (0.32 to 9.72) | 1.6% (1.49 to 1.71) |
| Hungary | 0.659 | 0.791 | 4414 (777 to 9775) | 30.86 (5.53 to 68.12) | 5017 (731 to 11526) | 28.26 (4.34 to 64.29) | -0.17% (-0.29 to -0.04) |
| Iceland | 0.764 | 0.869 | 91 (36 to 140) | 32.71 (13.07 to 50.29) | 118 (39 to 194) | 22.51 (7.75 to 36.66) | -1.4% (-1.49 to -1.31) |
| India | 0.327 | 0.566 | 5188 (1734 to 10560) | 1.06 (0.34 to 2.17) | 16620 (4870 to 35948) | 1.42 (0.41 to 3.07) | 0.83% (0.64 to 1.03) |
| Indonesia | 0.452 | 0.66 | 2943 (484 to 7473) | 2.6 (0.44 to 6.49) | 10847 (1565 to 28665) | 4.61 (0.67 to 12.07) | 1.86% (1.78 to 1.95) |
| Iran (Islamic Republic of) | 0.404 | 0.67 | 1165 (100 to 3188) | 4 (0.35 to 10.91) | 3487 (352 to 9173) | 4.44 (0.45 to 11.68) | 0.46% (0.25 to 0.68) |
| Iraq | 0.392 | 0.671 | 250 (27 to 698) | 2.94 (0.33 to 8.16) | 685 (96 to 1702) | 2.59 (0.38 to 6.27) | 0.08% (-0.5 to 0.66) |
| Ireland | 0.73 | 0.867 | 2063 (714 to 3329) | 52.37 (18.13 to 84.07) | 3021 (1339 to 4597) | 41.63 (18.8 to 62.97) | -0.92% (-1.04 to -0.81) |
| Israel | 0.717 | 0.803 | 610 (50 to 1653) | 12.86 (1.05 to 34.76) | 1403 (184 to 3360) | 12.65 (1.72 to 30.04) | -0.79% (-1.15 to -0.43) |
| Italy | 0.712 | 0.801 | 26851 (8065 to 46695) | 31.79 (9.71 to 54.7) | 28581 (8047 to 52269) | 22.68 (6.49 to 40.99) | -1.48% (-1.63 to -1.32) |
| Jamaica | 0.542 | 0.684 | 88 (8 to 238) | 5.04 (0.48 to 13.55) | 236 (23 to 660) | 7.92 (0.76 to 22.22) | 1.47% (1.08 to 1.86) |
| Japan | 0.791 | 0.87 | 19223 (1833 to 51170) | 11.37 (1.09 to 30.19) | 28520 (2882 to 74316) | 9.66 (1.01 to 25.01) | -0.89% (-1 to -0.78) |
| Jordan | 0.52 | 0.731 | 105 (9 to 297) | 6.79 (0.59 to 19.01) | 479 (41 to 1328) | 6.52 (0.58 to 17.8) | 0.13% (0 to 0.25) |
| Kazakhstan | 0.602 | 0.723 | 4398 (1383 to 7352) | 32.26 (9.93 to 54.39) | 4590 (1443 to 7859) | 25.02 (7.71 to 43.12) | -0.3% (-0.87 to 0.26) |
| Kenya | 0.333 | 0.508 | 255 (23 to 722) | 2.8 (0.25 to 7.92) | 1114 (106 to 3046) | 4.44 (0.42 to 12.12) | 2.05% (1.83 to 2.27) |
| Kiribati | 0.425 | 0.527 | 3 (0 to 7) | 6.2 (0.57 to 16.85) | 5 (0 to 13) | 5.76 (0.52 to 16.1) | -0.43% (-0.5 to -0.35) |
| Kuwait | 0.655 | 0.851 | 51 (7 to 118) | 6.42 (0.84 to 15.48) | 235 (32 to 550) | 7.74 (0.95 to 19.15) | 1.28% (0.64 to 1.93) |
| Kyrgyzstan | 0.532 | 0.596 | 645 (165 to 1184) | 20.07 (5.06 to 37.07) | 394 (62 to 865) | 7.91 (1.19 to 17.6) | -3.6% (-4.11 to -3.09) |
| Lao People's Democratic Republic | 0.268 | 0.49 | 119 (11 to 357) | 5.1 (0.49 to 15) | 398 (29 to 1185) | 8.05 (0.6 to 23.42) | 1.49% (1.43 to 1.55) |
| Latvia | 0.675 | 0.82 | 718 (128 to 1569) | 20.48 (3.71 to 44.44) | 548 (63 to 1333) | 15.06 (1.8 to 36.06) | -0.93% (-1.35 to -0.51) |
| Lebanon | 0.462 | 0.708 | 185 (14 to 520) | 7.9 (0.61 to 22.23) | 585 (50 to 1591) | 11.15 (0.94 to 30.41) | 1.74% (1.55 to 1.92) |
| Lesotho | 0.321 | 0.507 | 38 (3 to 112) | 3.73 (0.28 to 10.96) | 97 (7 to 282) | 7.1 (0.53 to 20.8) | 2.8% (2.6 to 3) |
| Liberia | 0.221 | 0.37 | 25 (3 to 64) | 2.18 (0.3 to 5.53) | 42 (5 to 120) | 1.85 (0.25 to 5.2) | 0.09% (-0.44 to 0.63) |
| Libya | 0.405 | 0.709 | 176 (13 to 503) | 8.55 (0.62 to 24.45) | 412 (34 to 1177) | 7.04 (0.59 to 19.94) | -0.31% (-0.51 to -0.11) |
| Lithuania | 0.67 | 0.843 | 1082 (247 to 2088) | 24.38 (5.62 to 46.89) | 1099 (210 to 2314) | 21.27 (4.19 to 43.97) | -0.13% (-0.47 to 0.22) |
| Luxembourg | 0.815 | 0.895 | 253 (79 to 422) | 47.87 (15.11 to 79.1) | 281 (95 to 475) | 29.15 (10.16 to 48.97) | -2.07% (-2.36 to -1.78) |
| Madagascar | 0.265 | 0.396 | 252 (19 to 692) | 4.38 (0.34 to 12.09) | 466 (41 to 1346) | 3.57 (0.33 to 10.25) | -0.79% (-0.94 to -0.64) |
| Malawi | 0.213 | 0.384 | 46 (9 to 107) | 1.09 (0.23 to 2.5) | 160 (20 to 423) | 1.99 (0.26 to 5.17) | 2.55% (2.33 to 2.77) |
| Malaysia | 0.542 | 0.737 | 800 (73 to 2189) | 8.07 (0.75 to 22) | 2375 (236 to 6529) | 8.63 (0.87 to 23.5) | -0.48% (-0.77 to -0.18) |
| Maldives | 0.303 | 0.562 | 1 (0 to 3) | 1.36 (0.45 to 2.88) | 7 (1 to 16) | 2.03 (0.37 to 4.81) | 1.12% (1 to 1.24) |
| Mali | 0.126 | 0.263 | 181 (14 to 509) | 4.07 (0.32 to 11.35) | 496 (41 to 1379) | 5.32 (0.43 to 14.88) | 1.1% (1.01 to 1.18) |
| Malta | 0.666 | 0.801 | 77 (14 to 162) | 18.02 (3.4 to 37.88) | 124 (22 to 276) | 14.61 (2.77 to 31.48) | -0.95% (-1.14 to -0.75) |
| Marshall Islands | 0.398 | 0.544 | 1 (0 to 4) | 7.16 (0.63 to 19.08) | 3 (0 to 9) | 8.11 (0.7 to 22.59) | 0.41% (0.37 to 0.45) |
| Mauritania | 0.308 | 0.496 | 60 (5 to 166) | 5.72 (0.45 to 15.9) | 108 (9 to 297) | 5.04 (0.42 to 13.95) | -0.23% (-0.41 to -0.06) |
| Mauritius | 0.527 | 0.705 | 34 (3 to 91) | 4.27 (0.4 to 11.49) | 117 (10 to 330) | 6.63 (0.6 to 18.74) | 1.35% (1.16 to 1.53) |
| Mexico | 0.507 | 0.649 | 2710 (463 to 6052) | 5.74 (0.92 to 13.02) | 12284 (2607 to 25985) | 10.03 (2.08 to 21.37) | 2.13% (2.03 to 2.23) |
| Micronesia (Federated States of) | 0.447 | 0.58 | 4 (0 to 11) | 7.69 (0.68 to 21.26) | 7 (1 to 19) | 8.49 (0.7 to 24.12) | 0.25% (0.21 to 0.3) |
| Monaco | 0.834 | 0.902 | 31 (12 to 52) | 48.83 (19.57 to 80.95) | 45 (18 to 75) | 53.1 (22.38 to 86.84) | 0.35% (0.3 to 0.4) |
| Mongolia | 0.465 | 0.606 | 368 (191 to 547) | 32.27 (16.69 to 48.15) | 804 (398 to 1284) | 29.73 (14.48 to 47.41) | -0.87% (-1.12 to -0.62) |
| Montenegro | 0.701 | 0.791 | 101 (18 to 219) | 15.74 (2.8 to 34.47) | 209 (41 to 424) | 21.75 (4.35 to 43.44) | 2.01% (1.68 to 2.33) |
| Morocco | 0.347 | 0.548 | 471 (45 to 1280) | 3.19 (0.31 to 8.63) | 1585 (127 to 4561) | 4.75 (0.39 to 13.46) | 1.42% (1.29 to 1.55) |
| Mozambique | 0.12 | 0.307 | 79 (14 to 192) | 1.25 (0.22 to 2.97) | 322 (41 to 868) | 2.68 (0.36 to 7.09) | 3.07% (2.92 to 3.23) |
| Myanmar | 0.284 | 0.521 | 678 (110 to 1740) | 2.61 (0.43 to 6.59) | 3404 (278 to 9858) | 6.83 (0.58 to 19.58) | 4.09% (3.79 to 4.38) |
| Namibia | 0.454 | 0.612 | 35 (3 to 90) | 4.57 (0.44 to 11.78) | 90 (8 to 246) | 6.01 (0.54 to 16.47) | 1.09% (0.96 to 1.21) |
| Nauru | 0.499 | 0.618 | 1 (0 to 2) | 15.06 (1.59 to 40.56) | 1 (0 to 2) | 12.27 (1.15 to 32.98) | -0.92% (-1.32 to -0.52) |
| Nepal | 0.198 | 0.422 | 200 (17 to 572) | 1.89 (0.16 to 5.38) | 605 (50 to 1747) | 2.64 (0.22 to 7.56) | 1.22% (0.92 to 1.52) |
| Netherlands | 0.796 | 0.883 | 6523 (1696 to 11772) | 34.07 (8.99 to 61.03) | 10927 (3176 to 18898) | 34.49 (10.38 to 59.05) | -0.03% (-0.11 to 0.06) |
| New Zealand | 0.757 | 0.84 | 2905 (1270 to 4386) | 76.37 (33.75 to 115.14) | 3372 (1391 to 5173) | 45.82 (19.27 to 70) | -1.95% (-2.13 to -1.76) |
| Nicaragua | 0.338 | 0.517 | 44 (5 to 116) | 2.6 (0.29 to 6.84) | 165 (20 to 430) | 3.6 (0.45 to 9.32) | 1.45% (1.25 to 1.65) |
| Niger | 0.0728 | 0.162 | 107 (8 to 317) | 3.46 (0.25 to 10.21) | 285 (22 to 818) | 3.42 (0.26 to 9.75) | 0.01% (-0.08 to 0.09) |
| Nigeria | 0.305 | 0.515 | 942 (112 to 2629) | 2.07 (0.25 to 5.74) | 2767 (302 to 7537) | 3.04 (0.34 to 8.13) | 1.84% (1.62 to 2.05) |
| Niue | 0.566 | 0.711 | 0 (0 to 1) | 9.04 (0.94 to 23.98) | 0 (0 to 1) | 9.64 (0.94 to 25.91) | 0.18% (0.12 to 0.24) |
| North Macedonia | 0.618 | 0.744 | 189 (15 to 512) | 9.68 (0.8 to 26.24) | 435 (35 to 1258) | 13.47 (1.09 to 38.8) | 1.3% (1.1 to 1.5) |
| Northern Mariana Islands | 0.692 | 0.771 | 4 (1 to 10) | 18 (2.72 to 41.26) | 8 (1 to 20) | 14.68 (1.75 to 36.09) | -0.8% (-0.93 to -0.66) |
| Norway | 0.807 | 0.913 | 1609 (352 to 3305) | 25.91 (5.92 to 52.45) | 2395 (676 to 4330) | 26.76 (7.73 to 47.8) | 0.17% (-0.05 to 0.4) |
| Oman | 0.441 | 0.783 | 38 (3 to 109) | 4.91 (0.36 to 14.18) | 136 (15 to 373) | 6.64 (0.64 to 18.06) | 1.64% (1.21 to 2.07) |
| Pakistan | 0.247 | 0.449 | 1714 (162 to 4691) | 2.81 (0.27 to 7.68) | 5846 (514 to 16510) | 4.61 (0.42 to 12.86) | 1.55% (1.33 to 1.78) |
| Palau | 0.621 | 0.738 | 1 (0 to 3) | 9.64 (0.94 to 24.89) | 2 (0 to 5) | 9.34 (0.86 to 24.59) | -0.14% (-0.23 to -0.06) |
| Palestine | 0.314 | 0.588 | 62 (6 to 174) | 6.69 (0.65 to 18.85) | 198 (23 to 516) | 7.53 (0.91 to 19.49) | 0.3% (0.03 to 0.56) |
| Panama | 0.544 | 0.686 | 105 (10 to 266) | 6.7 (0.66 to 17.15) | 368 (44 to 931) | 8.83 (1.05 to 22.34) | 1.24% (1.03 to 1.44) |
| Papua New Guinea | 0.292 | 0.394 | 85 (7 to 248) | 3.98 (0.32 to 11.55) | 248 (20 to 706) | 4.41 (0.37 to 12.69) | 0.19% (0.09 to 0.28) |
| Paraguay | 0.465 | 0.638 | 321 (118 to 515) | 13.55 (4.92 to 21.82) | 1214 (339 to 2236) | 20.84 (5.81 to 38.65) | 1.24% (1.05 to 1.42) |
| Peru | 0.501 | 0.648 | 432 (46 to 1169) | 3.4 (0.37 to 9.16) | 1416 (130 to 4084) | 4.36 (0.4 to 12.56) | 1.12% (0.92 to 1.31) |
| Philippines | 0.497 | 0.623 | 2585 (249 to 6891) | 7.37 (0.72 to 19.59) | 9702 (1006 to 25273) | 11.06 (1.15 to 28.84) | 1.19% (0.99 to 1.38) |
| Poland | 0.632 | 0.802 | 14222 (4141 to 25737) | 32.58 (9.55 to 58.94) | 26056 (7975 to 46972) | 39.24 (12.37 to 70.35) | 0.76% (0.64 to 0.87) |
| Portugal | 0.607 | 0.743 | 2986 (537 to 6537) | 22.55 (4.15 to 48.82) | 8764 (3163 to 14222) | 41.84 (15.83 to 66.42) | 1.93% (1.22 to 2.65) |
| Puerto Rico | 0.67 | 0.814 | 288 (27 to 766) | 7.99 (0.74 to 21.28) | 612 (55 to 1717) | 9.83 (0.89 to 27.55) | 0.65% (0.42 to 0.88) |
| Qatar | 0.585 | 0.83 | 11 (1 to 29) | 8.1 (0.77 to 21.46) | 88 (9 to 239) | 8.47 (0.78 to 23.08) | 0.77% (0.44 to 1.09) |
| Republic of Korea | 0.686 | 0.878 | 2437 (206 to 6699) | 7.36 (0.63 to 20.1) | 11978 (2314 to 25175) | 13.82 (2.77 to 28.58) | 1.96% (1.35 to 2.57) |
| Republic of Moldova | 0.585 | 0.696 | 631 (50 to 1717) | 13.6 (1.08 to 37.05) | 649 (54 to 1784) | 11.32 (0.94 to 31.13) | 0.25% (-0.43 to 0.93) |
| Romania | 0.625 | 0.76 | 3608 (460 to 8658) | 12.82 (1.67 to 30.55) | 12007 (3208 to 22128) | 36.04 (9.57 to 65) | 4.03% (3.78 to 4.29) |
| Russian Federation | 0.695 | 0.805 | 53724 (16422 to 94290) | 29.27 (8.95 to 51.47) | 43476 (7927 to 93297) | 19.1 (3.47 to 40.79) | -2.54% (-2.94 to -2.14) |
| Rwanda | 0.257 | 0.429 | 82 (13 to 213) | 2.52 (0.4 to 6.48) | 207 (25 to 561) | 3.04 (0.39 to 8.01) | 0.48% (0.2 to 0.77) |
| Saint Kitts and Nevis | 0.583 | 0.746 | 4 (0 to 10) | 10.76 (0.91 to 29.69) | 7 (1 to 20) | 10.44 (0.86 to 29.19) | -0.39% (-0.56 to -0.21) |
| Saint Lucia | 0.483 | 0.67 | 8 (1 to 20) | 8.66 (0.81 to 22.5) | 18 (2 to 48) | 8.3 (0.76 to 22.3) | -0.79% (-1.08 to -0.51) |
| Saint Vincent and the Grenadines | 0.462 | 0.627 | 4 (0 to 10) | 5.16 (0.55 to 13.46) | 10 (1 to 28) | 7.46 (0.68 to 20.72) | 1.23% (1.12 to 1.34) |
| Samoa | 0.531 | 0.641 | 7 (1 to 19) | 7.63 (0.74 to 20.41) | 12 (1 to 31) | 7.48 (0.74 to 19.9) | -0.07% (-0.11 to -0.03) |
| San Marino | 0.814 | 0.884 | 12 (4 to 21) | 35.54 (10.88 to 63.51) | 19 (5 to 38) | 31.71 (8.54 to 64.4) | -0.17% (-0.3 to -0.03) |
| Sao Tome and Principe | 0.299 | 0.502 | 1 (0 to 3) | 1.85 (0.54 to 3.97) | 3 (1 to 8) | 3.05 (0.73 to 7.07) | 1.86% (1.74 to 1.99) |
| Saudi Arabia | 0.48 | 0.805 | 235 (20 to 699) | 3.38 (0.29 to 10.04) | 1206 (104 to 3456) | 4.78 (0.45 to 13.29) | 1.16% (1.05 to 1.26) |
| Senegal | 0.227 | 0.389 | 117 (11 to 314) | 3.4 (0.34 to 9.13) | 292 (27 to 812) | 3.7 (0.35 to 10.19) | 0.53% (0.29 to 0.77) |
| Serbia | 0.626 | 0.767 | 1758 (147 to 4712) | 15.2 (1.27 to 40.92) | 2616 (233 to 7269) | 17.37 (1.57 to 48.08) | 0.77% (0.57 to 0.97) |
| Seychelles | 0.567 | 0.724 | 4 (1 to 9) | 6.23 (0.89 to 16.1) | 11 (1 to 29) | 9.94 (1.28 to 25.57) | 1.18% (0.94 to 1.43) |
| Sierra Leone | 0.207 | 0.347 | 25 (5 to 58) | 1.25 (0.26 to 2.89) | 67 (11 to 172) | 1.7 (0.29 to 4.32) | 1.45% (1.17 to 1.73) |
| Singapore | 0.688 | 0.861 | 470 (53 to 1137) | 19.7 (2.15 to 48.04) | 1220 (230 to 2559) | 15.19 (2.87 to 32.13) | -1.09% (-1.18 to -1.01) |
| Slovakia | 0.656 | 0.812 | 1400 (194 to 3241) | 23.62 (3.31 to 54.35) | 2554 (442 to 5779) | 28.53 (5.08 to 63.87) | 0.71% (0.46 to 0.96) |
| Slovenia | 0.726 | 0.84 | 767 (187 to 1495) | 31.58 (7.71 to 61.34) | 976 (190 to 2051) | 24.52 (5.01 to 50.89) | -1.16% (-1.6 to -0.72) |
| Solomon Islands | 0.279 | 0.407 | 8 (1 to 24) | 4.56 (0.39 to 13.91) | 20 (2 to 57) | 4.97 (0.53 to 14.18) | 0.28% (0.08 to 0.47) |
| Somalia | 0.0508 | 0.081 | 123 (9 to 373) | 4.05 (0.3 to 12.05) | 264 (17 to 874) | 3.32 (0.21 to 11.24) | -0.53% (-0.61 to -0.45) |
| South Africa | 0.552 | 0.678 | 1888 (267 to 4650) | 8.24 (1.13 to 20.63) | 4594 (797 to 10421) | 9.65 (1.6 to 22.24) | 0.95% (0.76 to 1.13) |
| South Sudan | 0.248 | 0.363 | 126 (10 to 412) | 4.9 (0.39 to 15.97) | 203 (17 to 610) | 4.73 (0.42 to 14.26) | -0.02% (-0.14 to 0.1) |
| Spain | 0.647 | 0.767 | 15586 (4228 to 27573) | 30.21 (8.36 to 53.05) | 23330 (5865 to 43065) | 26.98 (6.98 to 48.79) | -0.92% (-1.08 to -0.75) |
| Sri Lanka | 0.504 | 0.69 | 87 (27 to 177) | 0.75 (0.24 to 1.51) | 250 (66 to 564) | 0.98 (0.26 to 2.21) | 1.12% (1 to 1.24) |
| Sudan | 0.227 | 0.515 | 256 (23 to 725) | 2.5 (0.23 to 7.01) | 733 (67 to 2202) | 3.46 (0.32 to 10.1) | 1.26% (1.14 to 1.38) |
| Suriname | 0.498 | 0.636 | 13 (2 to 34) | 4.82 (0.62 to 12.38) | 39 (5 to 102) | 6.43 (0.8 to 16.59) | 1.16% (0.85 to 1.46) |
| Sweden | 0.769 | 0.872 | 2937 (630 to 5981) | 21.72 (4.99 to 43.08) | 4130 (1078 to 7618) | 22.29 (6.23 to 40) | 0.07% (-0.03 to 0.17) |
| Switzerland | 0.868 | 0.929 | 2821 (922 to 4558) | 28.89 (9.73 to 46.34) | 3384 (1079 to 5790) | 21.32 (6.92 to 35.72) | -1.22% (-1.31 to -1.13) |
| Syrian Arab Republic | 0.367 | 0.619 | 204 (16 to 581) | 3.38 (0.26 to 9.6) | 418 (34 to 1201) | 3.24 (0.27 to 9.26) | -0.3% (-0.54 to -0.07) |
| Taiwan (Province of China) | 0.667 | 0.868 | 2755 (406 to 6301) | 15.76 (2.26 to 36.57) | 17405 (5045 to 32721) | 45.67 (13.34 to 85.45) | 3.88% (3.51 to 4.25) |
| Tajikistan | 0.468 | 0.539 | 191 (17 to 524) | 6.11 (0.54 to 16.77) | 275 (25 to 761) | 4.8 (0.46 to 13.1) | -0.3% (-1.01 to 0.41) |
| Thailand | 0.508 | 0.687 | 2122 (207 to 5682) | 5.3 (0.53 to 14.11) | 5826 (489 to 17246) | 5.74 (0.49 to 16.99) | -0.39% (-0.7 to -0.08) |
| Timor-Leste | 0.274 | 0.514 | 12 (1 to 33) | 3.3 (0.35 to 9.15) | 48 (4 to 137) | 5.66 (0.54 to 15.96) | 2.19% (1.9 to 2.48) |
| Togo | 0.266 | 0.417 | 30 (4 to 73) | 2.13 (0.32 to 5.24) | 93 (12 to 250) | 2.31 (0.32 to 6.14) | 0.33% (0.24 to 0.43) |
| Tokelau | 0.427 | 0.626 | 0 (0 to 0) | 6.51 (0.56 to 18.57) | 0 (0 to 0) | 7.27 (0.64 to 19.82) | 0.42% (0.34 to 0.49) |
| Tonga | 0.51 | 0.636 | 2 (0 to 6) | 3.74 (0.34 to 9.79) | 3 (0 to 9) | 4.23 (0.38 to 11.76) | 0.27% (0.16 to 0.39) |
| Trinidad and Tobago | 0.618 | 0.757 | 58 (6 to 154) | 6.71 (0.72 to 17.75) | 133 (12 to 384) | 7.17 (0.63 to 20.54) | 0.42% (0.26 to 0.58) |
| Tunisia | 0.434 | 0.672 | 207 (18 to 581) | 3.96 (0.35 to 11.1) | 591 (52 to 1732) | 4.59 (0.41 to 13.41) | 0.35% (0.26 to 0.43) |
| Turkey | 0.473 | 0.748 | 2966 (249 to 8315) | 7.57 (0.65 to 21.13) | 6121 (521 to 16995) | 6.78 (0.58 to 18.85) | -0.21% (-0.79 to 0.38) |
| Turkmenistan | 0.548 | 0.67 | 249 (50 to 505) | 11 (2.12 to 22.45) | 658 (229 to 1145) | 15.18 (5.1 to 26.55) | 1.75% (0.87 to 2.64) |
| Tuvalu | 0.426 | 0.589 | 0 (0 to 1) | 6.54 (0.56 to 17.79) | 1 (0 to 2) | 7.04 (0.58 to 19.68) | 0.06% (0 to 0.12) |
| Uganda | 0.167 | 0.404 | 195 (21 to 507) | 2.78 (0.31 to 7.21) | 890 (82 to 2509) | 5.5 (0.52 to 15.22) | 2.4% (2.13 to 2.67) |
| Ukraine | 0.653 | 0.736 | 16567 (2424 to 38181) | 23.62 (3.54 to 54.16) | 11888 (1195 to 31985) | 16.56 (1.71 to 44.27) | -1.89% (-2.44 to -1.34) |
| United Arab Emirates | 0.621 | 0.88 | 119 (31 to 252) | 19.79 (4.24 to 45.11) | 496 (39 to 1443) | 9.57 (0.75 to 28.34) | -2.58% (-2.71 to -2.45) |
| United Kingdom | 0.745 | 0.847 | 25378 (6600 to 48809) | 30.02 (8.05 to 57.01) | 20179 (4592 to 41781) | 17.52 (4.08 to 35.68) | -2.15% (-2.44 to -1.86) |
| United Republic of Tanzania | 0.26 | 0.423 | 420 (42 to 1113) | 3.54 (0.37 to 9.32) | 1085 (115 to 2955) | 3.96 (0.44 to 10.47) | 0.45% (0.22 to 0.67) |
| United States of America | 0.768 | 0.859 | 101155 (30173 to 175828) | 33.24 (10.14 to 57.38) | 131190 (39078 to 226382) | 25.84 (7.77 to 44.42) | -1.03% (-1.15 to -0.92) |
| United States Virgin Islands | 0.667 | 0.799 | 10 (1 to 27) | 11.1 (1.03 to 30.37) | 29 (3 to 78) | 16.1 (1.5 to 43.99) | 1.62% (1.35 to 1.89) |
| Uruguay | 0.581 | 0.697 | 2477 (1068 to 3782) | 65.17 (28.14 to 99.33) | 2543 (883 to 4214) | 50.26 (17.93 to 82.33) | -1.23% (-1.38 to -1.07) |
| Uzbekistan | 0.49 | 0.631 | 905 (99 to 2191) | 7.09 (0.76 to 17.44) | 2297 (397 to 5156) | 9.56 (1.51 to 22.21) | 0.86% (0.49 to 1.23) |
| Vanuatu | 0.361 | 0.485 | 6 (1 to 14) | 7.28 (0.93 to 18.25) | 15 (2 to 41) | 8.08 (0.82 to 21.41) | 0.06% (-0.17 to 0.28) |
| Venezuela (Bolivarian Republic of) | 0.509 | 0.607 | 618 (51 to 1653) | 5.84 (0.47 to 15.77) | 2468 (230 to 6826) | 8.26 (0.77 to 22.78) | 1.08% (0.97 to 1.2) |
| Viet Nam | 0.39 | 0.617 | 1995 (181 to 5447) | 4.79 (0.44 to 13.08) | 15490 (1748 to 38141) | 15.32 (1.68 to 38.06) | 4.68% (4.43 to 4.93) |
| Yemen | 0.176 | 0.412 | 128 (12 to 369) | 2.31 (0.23 to 6.6) | 442 (46 to 1210) | 2.86 (0.3 to 7.87) | 1.41% (1.2 to 1.63) |
| Zambia | 0.299 | 0.505 | 158 (14 to 442) | 4.88 (0.46 to 13.67) | 486 (42 to 1416) | 6.1 (0.55 to 17.42) | 0.66% (0.58 to 0.73) |
| Zimbabwe | 0.394 | 0.476 | 186 (19 to 494) | 4.28 (0.45 to 11.27) | 481 (44 to 1344) | 6.14 (0.58 to 17) | 1.44% (1.11 to 1.78) |
