# Supplementary material for: Global disease burden linked to diet high in red meat and colorectal cancer from 1990 to 2019 and its prediction up to 2030
Source: Front Nutr. 2024 Mar 14;11:1366553. doi: 10.3389/fnut.2024.1366553 (PMC10973012; doi:10.3389/fnut.2024.1366553)
Supplement: Supplementary file 11 [file Table_3.DOCX]

Table S3. SDI by year for each country

| location | region | Super_Region | 1990 | 1991 | 1992 | 1993 | 1994 | 1995 | 1996 | 1997 | 1998 | 1999 | 2000 | 2001 | 2002 | 2003 | 2004 | 2005 | 2006 | 2007 | 2008 | 2009 | 2010 | 2011 | 2012 | 2013 | 2014 | 2015 | 2016 | 2017 | 2018 | 2019 |
| --- | --- | --- | --- | --- | --- | --- | --- | --- | --- | --- | --- | --- | --- | --- | --- | --- | --- | --- | --- | --- | --- | --- | --- | --- | --- | --- | --- | --- | --- | --- | --- | --- |
| Afghanistan | North Africa and Middle East | North Africa and Middle East | 0.1870 | 0.1910 | 0.1950 | 0.1960 | 0.1940 | 0.1940 | 0.1930 | 0.1920 | 0.1900 | 0.1890 | 0.1880 | 0.1880 | 0.1940 | 0.2020 | 0.2090 | 0.2160 | 0.2240 | 0.2340 | 0.2430 | 0.2530 | 0.2640 | 0.2740 | 0.2850 | 0.2950 | 0.3040 | 0.3130 | 0.321 | 0.3290 | 0.3370 | 0.343 |
| Albania | Central Europe | Central Europe, eastern Europe, and central Asia | 0.5400 | 0.5370 | 0.5340 | 0.5330 | 0.5350 | 0.5380 | 0.5440 | 0.5490 | 0.5550 | 0.5610 | 0.5690 | 0.5770 | 0.5850 | 0.5930 | 0.6010 | 0.6080 | 0.6150 | 0.6210 | 0.6270 | 0.6310 | 0.6360 | 0.6400 | 0.6450 | 0.6510 | 0.6580 | 0.6640 | 0.669 | 0.6740 | 0.6780 | 0.681 |
| Algeria | North Africa and Middle East | North Africa and Middle East | 0.4360 | 0.4460 | 0.4560 | 0.4650 | 0.4740 | 0.4830 | 0.4920 | 0.5000 | 0.5090 | 0.5180 | 0.5260 | 0.5340 | 0.5420 | 0.5500 | 0.5580 | 0.5660 | 0.5730 | 0.5800 | 0.5870 | 0.5930 | 0.5990 | 0.6050 | 0.6110 | 0.6170 | 0.6230 | 0.6280 | 0.634 | 0.6400 | 0.6460 | 0.652 |
| American Samoa | Oceania | Southeast Asia, east Asia, and Oceania | 0.6060 | 0.6090 | 0.6130 | 0.6160 | 0.6190 | 0.6230 | 0.6270 | 0.6300 | 0.6340 | 0.6370 | 0.6410 | 0.6450 | 0.6490 | 0.6520 | 0.6560 | 0.6600 | 0.6630 | 0.6670 | 0.6710 | 0.6740 | 0.6780 | 0.6820 | 0.6860 | 0.6900 | 0.6940 | 0.6980 | 0.702 | 0.7060 | 0.7090 | 0.712 |
| Andorra | Western Europe | High income | 0.8340 | 0.8380 | 0.8400 | 0.8410 | 0.8410 | 0.8410 | 0.8430 | 0.8450 | 0.8470 | 0.8490 | 0.8510 | 0.8540 | 0.8550 | 0.8590 | 0.8620 | 0.8650 | 0.8670 | 0.8690 | 0.8720 | 0.8740 | 0.8760 | 0.8790 | 0.8810 | 0.8830 | 0.8850 | 0.8870 | 0.889 | 0.8910 | 0.8920 | 0.894 |
| Angola | Central Sub-Saharan Africa | Sub-Saharan Africa | 0.2380 | 0.2430 | 0.2470 | 0.2500 | 0.2530 | 0.2570 | 0.2620 | 0.2670 | 0.2730 | 0.2780 | 0.2830 | 0.2890 | 0.2960 | 0.3030 | 0.3110 | 0.3190 | 0.3300 | 0.3410 | 0.3530 | 0.3640 | 0.3760 | 0.3870 | 0.3980 | 0.4100 | 0.4210 | 0.4320 | 0.443 | 0.4540 | 0.4630 | 0.470 |
| Antigua and Barbuda | Caribbean | Latin America and Caribbean | 0.5790 | 0.5860 | 0.5920 | 0.5980 | 0.6040 | 0.6100 | 0.6160 | 0.6230 | 0.6310 | 0.6380 | 0.6450 | 0.6520 | 0.6590 | 0.6650 | 0.6720 | 0.6790 | 0.6860 | 0.6940 | 0.7000 | 0.7050 | 0.7090 | 0.7130 | 0.7160 | 0.7190 | 0.7230 | 0.7270 | 0.731 | 0.7350 | 0.7390 | 0.743 |
| Argentina | Southern Latin America | Latin America and Caribbean | 0.5810 | 0.5850 | 0.5930 | 0.5990 | 0.6050 | 0.6110 | 0.6170 | 0.6220 | 0.6250 | 0.6280 | 0.6340 | 0.6370 | 0.6400 | 0.6410 | 0.6420 | 0.6490 | 0.6530 | 0.6550 | 0.6570 | 0.6610 | 0.6650 | 0.6700 | 0.6740 | 0.6770 | 0.6790 | 0.6870 | 0.696 | 0.7020 | 0.7060 | 0.708 |
| Armenia | Central Asia | Central Europe, eastern Europe, and central Asia | 0.5360 | 0.5410 | 0.5410 | 0.5420 | 0.5440 | 0.5460 | 0.5500 | 0.5540 | 0.5590 | 0.5640 | 0.5700 | 0.5770 | 0.5860 | 0.5960 | 0.6060 | 0.6160 | 0.6260 | 0.6370 | 0.6470 | 0.6520 | 0.6580 | 0.6620 | 0.6660 | 0.6700 | 0.6730 | 0.6760 | 0.679 | 0.6820 | 0.6860 | 0.689 |
| Australia | Australasia | High income | 0.7380 | 0.7410 | 0.7450 | 0.7490 | 0.7530 | 0.7570 | 0.7610 | 0.7660 | 0.7700 | 0.7740 | 0.7780 | 0.7820 | 0.7870 | 0.7910 | 0.7950 | 0.7970 | 0.7980 | 0.7990 | 0.8020 | 0.8060 | 0.8090 | 0.8120 | 0.8150 | 0.8200 | 0.8240 | 0.8280 | 0.832 | 0.8340 | 0.8370 | 0.839 |
| Austria | Western Europe | High income | 0.7530 | 0.7540 | 0.7570 | 0.7610 | 0.7680 | 0.7730 | 0.7780 | 0.7830 | 0.7870 | 0.7910 | 0.7950 | 0.7990 | 0.8030 | 0.8050 | 0.8080 | 0.8110 | 0.8150 | 0.8180 | 0.8210 | 0.8240 | 0.8260 | 0.8300 | 0.8330 | 0.8350 | 0.8380 | 0.8390 | 0.841 | 0.8440 | 0.8470 | 0.849 |
| Azerbaijan | Central Asia | Central Europe, eastern Europe, and central Asia | 0.5760 | 0.5780 | 0.5790 | 0.5780 | 0.5760 | 0.5730 | 0.5690 | 0.5650 | 0.5610 | 0.5590 | 0.5590 | 0.5610 | 0.5640 | 0.5690 | 0.5750 | 0.5830 | 0.5940 | 0.6070 | 0.6190 | 0.6280 | 0.6370 | 0.6450 | 0.6520 | 0.6580 | 0.6640 | 0.6690 | 0.673 | 0.6770 | 0.6800 | 0.683 |
| Bahamas | Caribbean | Latin America and Caribbean | 0.6920 | 0.6840 | 0.6760 | 0.6790 | 0.6860 | 0.6920 | 0.6970 | 0.7020 | 0.7100 | 0.7210 | 0.7310 | 0.7350 | 0.7370 | 0.7390 | 0.7420 | 0.7490 | 0.7550 | 0.7590 | 0.7620 | 0.7670 | 0.7740 | 0.7790 | 0.7800 | 0.7820 | 0.7840 | 0.7860 | 0.789 | 0.7910 | 0.7940 | 0.796 |
| Bahrain | North Africa and Middle East | North Africa and Middle East | 0.5530 | 0.5600 | 0.5660 | 0.5730 | 0.5800 | 0.5870 | 0.5950 | 0.6020 | 0.6100 | 0.6180 | 0.6260 | 0.6340 | 0.6430 | 0.6530 | 0.6620 | 0.6720 | 0.6800 | 0.6880 | 0.6970 | 0.7050 | 0.7110 | 0.7150 | 0.7200 | 0.7260 | 0.7310 | 0.7350 | 0.739 | 0.7430 | 0.7470 | 0.751 |
| Bangladesh | South Asia | South Asia | 0.2670 | 0.2750 | 0.2830 | 0.2900 | 0.2970 | 0.3040 | 0.3110 | 0.3170 | 0.3240 | 0.3300 | 0.3360 | 0.3430 | 0.3490 | 0.3550 | 0.3620 | 0.3690 | 0.3760 | 0.3840 | 0.3920 | 0.4000 | 0.4080 | 0.4160 | 0.4250 | 0.4330 | 0.4410 | 0.4490 | 0.457 | 0.4660 | 0.4750 | 0.483 |
| Barbados | Caribbean | Latin America and Caribbean | 0.6490 | 0.6530 | 0.6580 | 0.6630 | 0.6670 | 0.6700 | 0.6720 | 0.6740 | 0.6760 | 0.6770 | 0.6800 | 0.6830 | 0.6870 | 0.6910 | 0.6950 | 0.6990 | 0.7030 | 0.7070 | 0.7110 | 0.7140 | 0.7180 | 0.7210 | 0.7250 | 0.7280 | 0.7300 | 0.7330 | 0.735 | 0.7370 | 0.7400 | 0.742 |
| Belarus | Eastern Europe | Central Europe, eastern Europe, and central Asia | 0.5910 | 0.5950 | 0.6000 | 0.6060 | 0.6110 | 0.6140 | 0.6180 | 0.6200 | 0.6210 | 0.6240 | 0.6290 | 0.6350 | 0.6420 | 0.6500 | 0.6580 | 0.6650 | 0.6710 | 0.6780 | 0.6870 | 0.6950 | 0.7030 | 0.7090 | 0.7130 | 0.7190 | 0.7250 | 0.7300 | 0.734 | 0.7380 | 0.7420 | 0.745 |
| Belgium | Western Europe | High income | 0.7460 | 0.7500 | 0.7560 | 0.7620 | 0.7670 | 0.7710 | 0.7750 | 0.7790 | 0.7820 | 0.7840 | 0.7870 | 0.7920 | 0.7960 | 0.7990 | 0.8020 | 0.8050 | 0.8080 | 0.8100 | 0.8130 | 0.8160 | 0.8200 | 0.8240 | 0.8290 | 0.8340 | 0.8370 | 0.8410 | 0.843 | 0.8460 | 0.8490 | 0.851 |
| Belize | Caribbean | Latin America and Caribbean | 0.4280 | 0.4370 | 0.4470 | 0.4570 | 0.4660 | 0.4750 | 0.4820 | 0.4880 | 0.4940 | 0.4990 | 0.5050 | 0.5100 | 0.5150 | 0.5210 | 0.5280 | 0.5340 | 0.5410 | 0.5470 | 0.5530 | 0.5590 | 0.5640 | 0.5690 | 0.5740 | 0.5790 | 0.5830 | 0.5880 | 0.592 | 0.5960 | 0.6000 | 0.603 |
| Benin | Western Sub-Saharan Africa | Sub-Saharan Africa | 0.2090 | 0.2130 | 0.2180 | 0.2220 | 0.2270 | 0.2310 | 0.2360 | 0.2410 | 0.2450 | 0.2500 | 0.2540 | 0.2590 | 0.2630 | 0.2670 | 0.2710 | 0.2750 | 0.2790 | 0.2830 | 0.2880 | 0.2920 | 0.2970 | 0.3010 | 0.3060 | 0.3120 | 0.3180 | 0.3240 | 0.331 | 0.3380 | 0.3460 | 0.352 |
| Bermuda | Caribbean | Latin America and Caribbean | 0.6850 | 0.6890 | 0.6930 | 0.6970 | 0.7010 | 0.7040 | 0.7070 | 0.7100 | 0.7140 | 0.7190 | 0.7240 | 0.7310 | 0.7370 | 0.7430 | 0.7490 | 0.7550 | 0.7600 | 0.7660 | 0.7730 | 0.7790 | 0.7850 | 0.7900 | 0.7950 | 0.7990 | 0.8020 | 0.8050 | 0.807 | 0.8090 | 0.8110 | 0.813 |
| Bhutan | South Asia | South Asia | 0.2280 | 0.2320 | 0.2370 | 0.2430 | 0.2510 | 0.2580 | 0.2660 | 0.2750 | 0.2820 | 0.2900 | 0.2980 | 0.3060 | 0.3140 | 0.3220 | 0.3300 | 0.3380 | 0.3470 | 0.3560 | 0.3650 | 0.3750 | 0.3840 | 0.3940 | 0.4030 | 0.4110 | 0.4190 | 0.4260 | 0.434 | 0.4420 | 0.4490 | 0.455 |
| Bolivia (Plurinational State of) | Andean Latin America | Latin America and Caribbean | 0.4120 | 0.4170 | 0.4220 | 0.4280 | 0.4340 | 0.4410 | 0.4480 | 0.4550 | 0.4620 | 0.4690 | 0.4740 | 0.4800 | 0.4850 | 0.4910 | 0.4960 | 0.5010 | 0.5060 | 0.5100 | 0.5140 | 0.5180 | 0.5210 | 0.5250 | 0.5280 | 0.5330 | 0.5380 | 0.5440 | 0.550 | 0.5560 | 0.5620 | 0.566 |
| Bosnia and Herzegovina | Central Europe | Central Europe, eastern Europe, and central Asia | 0.5330 | 0.5340 | 0.5320 | 0.5290 | 0.5270 | 0.5280 | 0.5400 | 0.5580 | 0.5760 | 0.5910 | 0.6040 | 0.6160 | 0.6260 | 0.6360 | 0.6440 | 0.6510 | 0.6580 | 0.6650 | 0.6710 | 0.6770 | 0.6820 | 0.6860 | 0.6910 | 0.6950 | 0.6980 | 0.7020 | 0.706 | 0.7100 | 0.7140 | 0.718 |
| Botswana | Southern Sub-Saharan Africa | Sub-Saharan Africa | 0.4310 | 0.4410 | 0.4510 | 0.4590 | 0.4670 | 0.4750 | 0.4830 | 0.4910 | 0.4980 | 0.5060 | 0.5140 | 0.5210 | 0.5280 | 0.5350 | 0.5410 | 0.5480 | 0.5550 | 0.5620 | 0.5690 | 0.5750 | 0.5810 | 0.5870 | 0.5930 | 0.6000 | 0.6060 | 0.6120 | 0.618 | 0.6240 | 0.6300 | 0.634 |
| Brazil | Tropical Latin America | Latin America and Caribbean | 0.4870 | 0.4920 | 0.4980 | 0.5030 | 0.5080 | 0.5130 | 0.5190 | 0.5240 | 0.5290 | 0.5330 | 0.5380 | 0.5430 | 0.5470 | 0.5510 | 0.5560 | 0.5610 | 0.5660 | 0.5720 | 0.5770 | 0.5830 | 0.5900 | 0.5970 | 0.6030 | 0.6100 | 0.6160 | 0.6220 | 0.627 | 0.6320 | 0.6360 | 0.640 |
| Brunei Darussalam | High-income Asia Pacific | High income | 0.6760 | 0.6820 | 0.6880 | 0.6940 | 0.7000 | 0.7060 | 0.7120 | 0.7170 | 0.7230 | 0.7290 | 0.7350 | 0.7410 | 0.7470 | 0.7530 | 0.7580 | 0.7640 | 0.7690 | 0.7740 | 0.7790 | 0.7840 | 0.7890 | 0.7930 | 0.7970 | 0.8010 | 0.8060 | 0.8090 | 0.813 | 0.8170 | 0.8200 | 0.823 |
| Bulgaria | Central Europe | Central Europe, eastern Europe, and central Asia | 0.6310 | 0.6410 | 0.6480 | 0.6560 | 0.6660 | 0.6710 | 0.6760 | 0.6810 | 0.6770 | 0.6750 | 0.6800 | 0.6880 | 0.6930 | 0.6970 | 0.7010 | 0.7060 | 0.7100 | 0.7150 | 0.7180 | 0.7240 | 0.7330 | 0.7370 | 0.7400 | 0.7430 | 0.7460 | 0.7500 | 0.752 | 0.7550 | 0.7600 | 0.764 |
| Burkina Faso | Western Sub-Saharan Africa | Sub-Saharan Africa | 0.1250 | 0.1280 | 0.1310 | 0.1340 | 0.1370 | 0.1400 | 0.1440 | 0.1470 | 0.1520 | 0.1560 | 0.1610 | 0.1660 | 0.1700 | 0.1750 | 0.1800 | 0.1860 | 0.1910 | 0.1960 | 0.2000 | 0.2050 | 0.2100 | 0.2150 | 0.2200 | 0.2260 | 0.2310 | 0.2360 | 0.241 | 0.2470 | 0.2520 | 0.257 |
| Burundi | Eastern Sub-Saharan Africa | Sub-Saharan Africa | 0.1980 | 0.2010 | 0.2040 | 0.2070 | 0.2090 | 0.2100 | 0.2100 | 0.2110 | 0.2120 | 0.2130 | 0.2130 | 0.2140 | 0.2160 | 0.2180 | 0.2200 | 0.2230 | 0.2260 | 0.2300 | 0.2340 | 0.2380 | 0.2430 | 0.2480 | 0.2540 | 0.2600 | 0.2660 | 0.2700 | 0.274 | 0.2780 | 0.2820 | 0.284 |
| Cabo Verde | Western Sub-Saharan Africa | Sub-Saharan Africa | 0.2920 | 0.2980 | 0.3050 | 0.3110 | 0.3180 | 0.3260 | 0.3330 | 0.3420 | 0.3500 | 0.3600 | 0.3700 | 0.3790 | 0.3880 | 0.3970 | 0.4070 | 0.4150 | 0.4240 | 0.4340 | 0.4430 | 0.4520 | 0.4610 | 0.4690 | 0.4770 | 0.4840 | 0.4910 | 0.4980 | 0.505 | 0.5120 | 0.5190 | 0.525 |
| Cambodia | Southeast Asia | Southeast Asia, east Asia, and Oceania | 0.2660 | 0.2720 | 0.2770 | 0.2820 | 0.2860 | 0.2910 | 0.2960 | 0.3010 | 0.3070 | 0.3130 | 0.3210 | 0.3280 | 0.3370 | 0.3450 | 0.3540 | 0.3630 | 0.3730 | 0.3820 | 0.3910 | 0.3990 | 0.4060 | 0.4130 | 0.4210 | 0.4280 | 0.4350 | 0.4420 | 0.449 | 0.4560 | 0.4630 | 0.469 |
| Cameroon | Western Sub-Saharan Africa | Sub-Saharan Africa | 0.3130 | 0.3200 | 0.3250 | 0.3300 | 0.3340 | 0.3380 | 0.3420 | 0.3460 | 0.3490 | 0.3530 | 0.3570 | 0.3600 | 0.3640 | 0.3680 | 0.3730 | 0.3780 | 0.3840 | 0.3900 | 0.3970 | 0.4040 | 0.4120 | 0.4200 | 0.4280 | 0.4360 | 0.4450 | 0.4550 | 0.464 | 0.4740 | 0.4830 | 0.490 |
| Canada | High-income North America | High income | 0.7900 | 0.7920 | 0.7950 | 0.7970 | 0.8000 | 0.8040 | 0.8090 | 0.8120 | 0.8150 | 0.8190 | 0.8240 | 0.8280 | 0.8320 | 0.8350 | 0.8380 | 0.8400 | 0.8420 | 0.8430 | 0.8450 | 0.8480 | 0.8510 | 0.8530 | 0.8560 | 0.8590 | 0.8610 | 0.8640 | 0.867 | 0.8690 | 0.8710 | 0.873 |
| Central African Republic | Central Sub-Saharan Africa | Sub-Saharan Africa | 0.1860 | 0.1900 | 0.1930 | 0.1960 | 0.1990 | 0.2020 | 0.2050 | 0.2080 | 0.2110 | 0.2150 | 0.2180 | 0.2210 | 0.2250 | 0.2270 | 0.2300 | 0.2330 | 0.2360 | 0.2400 | 0.2440 | 0.2480 | 0.2530 | 0.2580 | 0.2630 | 0.2630 | 0.2630 | 0.2640 | 0.266 | 0.2680 | 0.2710 | 0.274 |
| Chad | Western Sub-Saharan Africa | Sub-Saharan Africa | 0.1080 | 0.1120 | 0.1150 | 0.1180 | 0.1200 | 0.1230 | 0.1250 | 0.1280 | 0.1300 | 0.1320 | 0.1350 | 0.1370 | 0.1400 | 0.1440 | 0.1490 | 0.1560 | 0.1620 | 0.1670 | 0.1730 | 0.1790 | 0.1850 | 0.1910 | 0.1970 | 0.2030 | 0.2100 | 0.2160 | 0.223 | 0.2280 | 0.2340 | 0.238 |
| Chile | Southern Latin America | Latin America and Caribbean | 0.5920 | 0.6000 | 0.6060 | 0.6110 | 0.6170 | 0.6240 | 0.6300 | 0.6370 | 0.6440 | 0.6510 | 0.6570 | 0.6630 | 0.6710 | 0.6780 | 0.6830 | 0.6860 | 0.6890 | 0.6920 | 0.6950 | 0.7000 | 0.7060 | 0.7120 | 0.7190 | 0.7240 | 0.7280 | 0.7380 | 0.747 | 0.7530 | 0.7560 | 0.759 |
| China | East Asia | Southeast Asia, east Asia, and Oceania | 0.4330 | 0.4410 | 0.4500 | 0.4590 | 0.4690 | 0.4790 | 0.4890 | 0.4990 | 0.5080 | 0.5160 | 0.5250 | 0.5340 | 0.5430 | 0.5520 | 0.5610 | 0.5710 | 0.5810 | 0.5910 | 0.6010 | 0.6110 | 0.6210 | 0.6310 | 0.6380 | 0.6460 | 0.6540 | 0.6570 | 0.659 | 0.6690 | 0.6790 | 0.686 |
| Colombia | Central Latin America | Latin America and Caribbean | 0.4780 | 0.4800 | 0.4820 | 0.4860 | 0.4920 | 0.4980 | 0.5030 | 0.5090 | 0.5140 | 0.5180 | 0.5220 | 0.5260 | 0.5290 | 0.5330 | 0.5360 | 0.5400 | 0.5460 | 0.5520 | 0.5590 | 0.5670 | 0.5740 | 0.5820 | 0.5890 | 0.5960 | 0.6030 | 0.6090 | 0.616 | 0.6220 | 0.6280 | 0.633 |
| Comoros | Eastern Sub-Saharan Africa | Sub-Saharan Africa | 0.2740 | 0.2810 | 0.2880 | 0.2960 | 0.3020 | 0.3090 | 0.3150 | 0.3220 | 0.3280 | 0.3340 | 0.3400 | 0.3460 | 0.3530 | 0.3590 | 0.3650 | 0.3710 | 0.3780 | 0.3840 | 0.3900 | 0.3960 | 0.4010 | 0.4070 | 0.4130 | 0.4190 | 0.4260 | 0.4320 | 0.438 | 0.4440 | 0.4500 | 0.455 |
| Congo | Central Sub-Saharan Africa | Sub-Saharan Africa | 0.3640 | 0.3730 | 0.3810 | 0.3890 | 0.3960 | 0.4030 | 0.4100 | 0.4160 | 0.4210 | 0.4260 | 0.4310 | 0.4370 | 0.4420 | 0.4470 | 0.4520 | 0.4580 | 0.4640 | 0.4690 | 0.4750 | 0.4820 | 0.4910 | 0.5000 | 0.5090 | 0.5190 | 0.5280 | 0.5380 | 0.547 | 0.5560 | 0.5630 | 0.568 |
| Cook Islands | Oceania | Southeast Asia, east Asia, and Oceania | 0.6250 | 0.6310 | 0.6370 | 0.6430 | 0.6490 | 0.6540 | 0.6590 | 0.6630 | 0.6670 | 0.6710 | 0.6760 | 0.6820 | 0.6870 | 0.6920 | 0.6970 | 0.7010 | 0.7060 | 0.7110 | 0.7150 | 0.7190 | 0.7220 | 0.7260 | 0.7300 | 0.7340 | 0.7390 | 0.7440 | 0.750 | 0.7550 | 0.7600 | 0.764 |
| Costa Rica | Central Latin America | Latin America and Caribbean | 0.5320 | 0.5380 | 0.5440 | 0.5500 | 0.5550 | 0.5600 | 0.5650 | 0.5710 | 0.5770 | 0.5840 | 0.5920 | 0.5980 | 0.6030 | 0.6070 | 0.6110 | 0.6150 | 0.6190 | 0.6230 | 0.6270 | 0.6320 | 0.6370 | 0.6420 | 0.6470 | 0.6520 | 0.6570 | 0.6620 | 0.667 | 0.6720 | 0.6760 | 0.680 |
| Croatia | Central Europe | Central Europe, eastern Europe, and central Asia | 0.6800 | 0.6880 | 0.6920 | 0.6920 | 0.6920 | 0.6910 | 0.6910 | 0.6970 | 0.7030 | 0.7070 | 0.7130 | 0.7190 | 0.7250 | 0.7300 | 0.7340 | 0.7390 | 0.7450 | 0.7480 | 0.7530 | 0.7580 | 0.7630 | 0.7670 | 0.7700 | 0.7740 | 0.7770 | 0.7810 | 0.784 | 0.7880 | 0.7910 | 0.794 |
| Cuba | Caribbean | Latin America and Caribbean | 0.5780 | 0.5860 | 0.5890 | 0.5880 | 0.5860 | 0.5830 | 0.5830 | 0.5820 | 0.5790 | 0.5810 | 0.5860 | 0.5880 | 0.5950 | 0.6020 | 0.6050 | 0.6110 | 0.6180 | 0.6180 | 0.6160 | 0.6180 | 0.6200 | 0.6240 | 0.6310 | 0.6360 | 0.6400 | 0.6450 | 0.653 | 0.6600 | 0.6650 | 0.668 |
| Cyprus | Western Europe | High income | 0.6620 | 0.6700 | 0.6800 | 0.6910 | 0.7020 | 0.7130 | 0.7230 | 0.7320 | 0.7410 | 0.7500 | 0.7580 | 0.7670 | 0.7740 | 0.7800 | 0.7860 | 0.7910 | 0.7970 | 0.8040 | 0.8100 | 0.8160 | 0.8200 | 0.8240 | 0.8270 | 0.8290 | 0.8310 | 0.8320 | 0.834 | 0.8360 | 0.8380 | 0.841 |
| Czechia | Central Europe | Central Europe, eastern Europe, and central Asia | 0.6880 | 0.6960 | 0.7050 | 0.7180 | 0.7360 | 0.7480 | 0.7550 | 0.7600 | 0.7650 | 0.7710 | 0.7760 | 0.7820 | 0.7860 | 0.7900 | 0.7940 | 0.7980 | 0.8010 | 0.8040 | 0.8070 | 0.8100 | 0.8130 | 0.8160 | 0.8180 | 0.8190 | 0.8200 | 0.8200 | 0.820 | 0.8220 | 0.8250 | 0.828 |
| C?te d'Ivoire | Western Sub-Saharan Africa | Sub-Saharan Africa | 0.2560 | 0.2600 | 0.2650 | 0.2700 | 0.2750 | 0.2790 | 0.2850 | 0.2900 | 0.2950 | 0.3010 | 0.3050 | 0.3100 | 0.3140 | 0.3180 | 0.3220 | 0.3250 | 0.3290 | 0.3330 | 0.3370 | 0.3410 | 0.3460 | 0.3500 | 0.3550 | 0.3620 | 0.3690 | 0.3760 | 0.384 | 0.3930 | 0.4010 | 0.408 |
| Democratic People's Republic of Korea | East Asia | Southeast Asia, east Asia, and Oceania | 0.4310 | 0.4360 | 0.4390 | 0.4420 | 0.4450 | 0.4470 | 0.4500 | 0.4510 | 0.4520 | 0.4550 | 0.4580 | 0.4630 | 0.4670 | 0.4730 | 0.4780 | 0.4850 | 0.4900 | 0.4960 | 0.5020 | 0.5070 | 0.5130 | 0.5180 | 0.5230 | 0.5280 | 0.5340 | 0.5380 | 0.543 | 0.5480 | 0.5530 | 0.558 |
| Democratic Republic of the Congo | Central Sub-Saharan Africa | Sub-Saharan Africa | 0.2600 | 0.2620 | 0.2630 | 0.2630 | 0.2620 | 0.2610 | 0.2590 | 0.2560 | 0.2530 | 0.2500 | 0.2460 | 0.2420 | 0.2390 | 0.2380 | 0.2390 | 0.2420 | 0.2440 | 0.2470 | 0.2510 | 0.2570 | 0.2660 | 0.2770 | 0.2890 | 0.3050 | 0.3210 | 0.3360 | 0.350 | 0.3620 | 0.3740 | 0.382 |
| Denmark | Western Europe | High income | 0.8060 | 0.8090 | 0.8130 | 0.8160 | 0.8200 | 0.8240 | 0.8280 | 0.8330 | 0.8360 | 0.8400 | 0.8440 | 0.8480 | 0.8520 | 0.8550 | 0.8580 | 0.8600 | 0.8620 | 0.8640 | 0.8650 | 0.8670 | 0.8700 | 0.8730 | 0.8750 | 0.8780 | 0.8800 | 0.8820 | 0.884 | 0.8860 | 0.8880 | 0.890 |
| Djibouti | Eastern Sub-Saharan Africa | Sub-Saharan Africa | 0.2750 | 0.2770 | 0.2800 | 0.2850 | 0.2890 | 0.2930 | 0.2970 | 0.3020 | 0.3060 | 0.3110 | 0.3170 | 0.3220 | 0.3270 | 0.3330 | 0.3390 | 0.3450 | 0.3530 | 0.3600 | 0.3680 | 0.3760 | 0.3840 | 0.3920 | 0.4000 | 0.4080 | 0.4160 | 0.4250 | 0.434 | 0.4430 | 0.4520 | 0.459 |
| Dominica | Caribbean | Latin America and Caribbean | 0.5790 | 0.5850 | 0.5910 | 0.5970 | 0.6040 | 0.6100 | 0.6170 | 0.6230 | 0.6300 | 0.6370 | 0.6430 | 0.6490 | 0.6550 | 0.6600 | 0.6660 | 0.6720 | 0.6770 | 0.6830 | 0.6890 | 0.6950 | 0.7000 | 0.7050 | 0.7090 | 0.7130 | 0.7170 | 0.7210 | 0.724 | 0.7270 | 0.7280 | 0.729 |
| Dominican Republic | Caribbean | Latin America and Caribbean | 0.4250 | 0.4270 | 0.4300 | 0.4340 | 0.4370 | 0.4420 | 0.4460 | 0.4520 | 0.4580 | 0.4650 | 0.4710 | 0.4780 | 0.4850 | 0.4920 | 0.4990 | 0.5080 | 0.5160 | 0.5240 | 0.5320 | 0.5380 | 0.5440 | 0.5490 | 0.5540 | 0.5590 | 0.5630 | 0.5690 | 0.574 | 0.5800 | 0.5870 | 0.592 |
| Ecuador | Andean Latin America | Latin America and Caribbean | 0.5030 | 0.5060 | 0.5100 | 0.5140 | 0.5170 | 0.5210 | 0.5240 | 0.5280 | 0.5320 | 0.5350 | 0.5390 | 0.5440 | 0.5490 | 0.5540 | 0.5590 | 0.5650 | 0.5700 | 0.5750 | 0.5810 | 0.5860 | 0.5910 | 0.5970 | 0.6030 | 0.6090 | 0.6150 | 0.6210 | 0.626 | 0.6320 | 0.6360 | 0.640 |
| Egypt | North Africa and Middle East | North Africa and Middle East | 0.4030 | 0.4150 | 0.4260 | 0.4370 | 0.4480 | 0.4590 | 0.4690 | 0.4780 | 0.4870 | 0.4960 | 0.5040 | 0.5120 | 0.5200 | 0.5270 | 0.5350 | 0.5420 | 0.5490 | 0.5570 | 0.5650 | 0.5730 | 0.5820 | 0.5910 | 0.6000 | 0.6090 | 0.6170 | 0.6260 | 0.635 | 0.6430 | 0.6510 | 0.658 |
| El Salvador | Central Latin America | Latin America and Caribbean | 0.3900 | 0.3930 | 0.3970 | 0.4020 | 0.4080 | 0.4150 | 0.4230 | 0.4310 | 0.4410 | 0.4500 | 0.4600 | 0.4690 | 0.4780 | 0.4860 | 0.4930 | 0.4990 | 0.5050 | 0.5110 | 0.5170 | 0.5210 | 0.5260 | 0.5310 | 0.5360 | 0.5420 | 0.5470 | 0.5520 | 0.558 | 0.5630 | 0.5680 | 0.573 |
| Equatorial Guinea | Central Sub-Saharan Africa | Sub-Saharan Africa | 0.2080 | 0.2140 | 0.2220 | 0.2300 | 0.2400 | 0.2520 | 0.2680 | 0.2950 | 0.3180 | 0.3410 | 0.3640 | 0.3910 | 0.4150 | 0.4380 | 0.4600 | 0.4820 | 0.5020 | 0.5220 | 0.5430 | 0.5610 | 0.5780 | 0.5940 | 0.6110 | 0.6260 | 0.6400 | 0.6520 | 0.663 | 0.6730 | 0.6810 | 0.685 |
| Eritrea | Eastern Sub-Saharan Africa | Sub-Saharan Africa | 0.1980 | 0.2030 | 0.2100 | 0.2180 | 0.2280 | 0.2380 | 0.2480 | 0.2580 | 0.2670 | 0.2750 | 0.2830 | 0.2890 | 0.2960 | 0.3020 | 0.3070 | 0.3120 | 0.3170 | 0.3220 | 0.3260 | 0.3300 | 0.3340 | 0.3400 | 0.3460 | 0.3530 | 0.3600 | 0.3670 | 0.375 | 0.3820 | 0.3900 | 0.396 |
| Estonia | Eastern Europe | Central Europe, eastern Europe, and central Asia | 0.6650 | 0.6760 | 0.6870 | 0.6960 | 0.7000 | 0.7050 | 0.7110 | 0.7170 | 0.7210 | 0.7260 | 0.7330 | 0.7410 | 0.7480 | 0.7530 | 0.7590 | 0.7650 | 0.7710 | 0.7770 | 0.7850 | 0.7920 | 0.7980 | 0.8040 | 0.8090 | 0.8130 | 0.8170 | 0.8210 | 0.825 | 0.8290 | 0.8330 | 0.835 |
| Eswatini | Southern Sub-Saharan Africa | Sub-Saharan Africa | 0.3920 | 0.4010 | 0.4100 | 0.4190 | 0.4270 | 0.4350 | 0.4430 | 0.4500 | 0.4560 | 0.4620 | 0.4680 | 0.4740 | 0.4790 | 0.4840 | 0.4900 | 0.4950 | 0.5010 | 0.5070 | 0.5130 | 0.5190 | 0.5260 | 0.5320 | 0.5380 | 0.5450 | 0.5510 | 0.5570 | 0.563 | 0.5690 | 0.5740 | 0.577 |
| Ethiopia | Eastern Sub-Saharan Africa | Sub-Saharan Africa | 0.1440 | 0.1460 | 0.1470 | 0.1490 | 0.1510 | 0.1530 | 0.1570 | 0.1610 | 0.1640 | 0.1670 | 0.1710 | 0.1760 | 0.1800 | 0.1830 | 0.1890 | 0.1950 | 0.2030 | 0.2130 | 0.2230 | 0.2330 | 0.2440 | 0.2560 | 0.2680 | 0.2790 | 0.2910 | 0.3020 | 0.313 | 0.3240 | 0.3340 | 0.343 |
| Fiji | Oceania | Southeast Asia, east Asia, and Oceania | 0.5270 | 0.5320 | 0.5380 | 0.5440 | 0.5500 | 0.5550 | 0.5610 | 0.5670 | 0.5720 | 0.5770 | 0.5820 | 0.5870 | 0.5920 | 0.5960 | 0.6010 | 0.6050 | 0.6090 | 0.6120 | 0.6160 | 0.6190 | 0.6220 | 0.6260 | 0.6300 | 0.6350 | 0.6390 | 0.6440 | 0.649 | 0.6540 | 0.6590 | 0.664 |
| Finland | Western Europe | High income | 0.7570 | 0.7590 | 0.7620 | 0.7650 | 0.7690 | 0.7730 | 0.7770 | 0.7820 | 0.7850 | 0.7880 | 0.7920 | 0.7970 | 0.8020 | 0.8050 | 0.8080 | 0.8120 | 0.8150 | 0.8180 | 0.8210 | 0.8240 | 0.8280 | 0.8310 | 0.8340 | 0.8370 | 0.8400 | 0.8440 | 0.848 | 0.8510 | 0.8530 | 0.856 |
| France | Western Europe | High income | 0.7380 | 0.7430 | 0.7500 | 0.7550 | 0.7590 | 0.7630 | 0.7670 | 0.7700 | 0.7730 | 0.7750 | 0.7770 | 0.7810 | 0.7850 | 0.7870 | 0.7900 | 0.7930 | 0.7960 | 0.7990 | 0.8010 | 0.8030 | 0.8060 | 0.8090 | 0.8120 | 0.8150 | 0.8190 | 0.8220 | 0.826 | 0.8290 | 0.8320 | 0.834 |
| Gabon | Central Sub-Saharan Africa | Sub-Saharan Africa | 0.3880 | 0.3990 | 0.4090 | 0.4200 | 0.4310 | 0.4420 | 0.4530 | 0.4630 | 0.4740 | 0.4830 | 0.4930 | 0.5020 | 0.5100 | 0.5190 | 0.5280 | 0.5370 | 0.5460 | 0.5540 | 0.5630 | 0.5700 | 0.5790 | 0.5870 | 0.5960 | 0.6050 | 0.6140 | 0.6230 | 0.632 | 0.6410 | 0.6490 | 0.656 |
| Gambia | Western Sub-Saharan Africa | Sub-Saharan Africa | 0.2180 | 0.2230 | 0.2270 | 0.2320 | 0.2370 | 0.2410 | 0.2460 | 0.2510 | 0.2560 | 0.2620 | 0.2680 | 0.2740 | 0.2800 | 0.2860 | 0.2930 | 0.3000 | 0.3060 | 0.3130 | 0.3190 | 0.3270 | 0.3340 | 0.3410 | 0.3480 | 0.3560 | 0.3630 | 0.3700 | 0.378 | 0.3850 | 0.3930 | 0.399 |
| Georgia | Central Asia | Central Europe, eastern Europe, and central Asia | 0.6540 | 0.6580 | 0.6570 | 0.6540 | 0.6500 | 0.6440 | 0.6380 | 0.6330 | 0.6280 | 0.6250 | 0.6240 | 0.6260 | 0.6300 | 0.6360 | 0.6410 | 0.6460 | 0.6510 | 0.6560 | 0.6600 | 0.6630 | 0.6650 | 0.6680 | 0.6720 | 0.6760 | 0.6800 | 0.6840 | 0.688 | 0.6930 | 0.6970 | 0.702 |
| Germany | Western Europe | High income | 0.8190 | 0.8300 | 0.8340 | 0.8380 | 0.8410 | 0.8430 | 0.8440 | 0.8440 | 0.8440 | 0.8440 | 0.8470 | 0.8530 | 0.8560 | 0.8580 | 0.8610 | 0.8630 | 0.8660 | 0.8690 | 0.8730 | 0.8750 | 0.8780 | 0.8810 | 0.8830 | 0.8860 | 0.8880 | 0.8900 | 0.892 | 0.8940 | 0.8960 | 0.898 |
| Ghana | Western Sub-Saharan Africa | Sub-Saharan Africa | 0.3550 | 0.3620 | 0.3680 | 0.3740 | 0.3800 | 0.3850 | 0.3910 | 0.3960 | 0.4010 | 0.4060 | 0.4110 | 0.4150 | 0.4200 | 0.4260 | 0.4310 | 0.4370 | 0.4440 | 0.4500 | 0.4580 | 0.4660 | 0.4740 | 0.4840 | 0.4940 | 0.5040 | 0.5140 | 0.5230 | 0.531 | 0.5410 | 0.5490 | 0.557 |
| Greece | Western Europe | High income | 0.6820 | 0.6880 | 0.6950 | 0.7020 | 0.7070 | 0.7130 | 0.7170 | 0.7230 | 0.7290 | 0.7350 | 0.7400 | 0.7430 | 0.7480 | 0.7530 | 0.7580 | 0.7610 | 0.7650 | 0.7680 | 0.7710 | 0.7750 | 0.7790 | 0.7820 | 0.7850 | 0.7860 | 0.7860 | 0.7870 | 0.788 | 0.7900 | 0.7920 | 0.794 |
| Greenland | High-income North America | High income | 0.6550 | 0.6520 | 0.6510 | 0.6510 | 0.6530 | 0.6530 | 0.6530 | 0.6550 | 0.6610 | 0.6640 | 0.6670 | 0.6710 | 0.6780 | 0.6840 | 0.6890 | 0.6960 | 0.7040 | 0.7100 | 0.7150 | 0.7210 | 0.7280 | 0.7340 | 0.7370 | 0.7400 | 0.7430 | 0.7470 | 0.751 | 0.7560 | 0.7590 | 0.761 |
| Grenada | Caribbean | Latin America and Caribbean | 0.4630 | 0.4740 | 0.4850 | 0.4950 | 0.5050 | 0.5150 | 0.5240 | 0.5330 | 0.5430 | 0.5530 | 0.5620 | 0.5700 | 0.5770 | 0.5850 | 0.5920 | 0.6000 | 0.6060 | 0.6130 | 0.6190 | 0.6240 | 0.6280 | 0.6330 | 0.6370 | 0.6410 | 0.6450 | 0.6500 | 0.654 | 0.6590 | 0.6640 | 0.669 |
| Guam | Oceania | Southeast Asia, east Asia, and Oceania | 0.6930 | 0.6880 | 0.6840 | 0.6840 | 0.6880 | 0.6950 | 0.7040 | 0.7150 | 0.7280 | 0.7410 | 0.7530 | 0.7620 | 0.7670 | 0.7680 | 0.7700 | 0.7710 | 0.7730 | 0.7750 | 0.7780 | 0.7820 | 0.7850 | 0.7890 | 0.7920 | 0.7950 | 0.7970 | 0.7990 | 0.803 | 0.8070 | 0.8100 | 0.813 |
| Guatemala | Central Latin America | Latin America and Caribbean | 0.3150 | 0.3200 | 0.3280 | 0.3360 | 0.3450 | 0.3520 | 0.3600 | 0.3690 | 0.3780 | 0.3870 | 0.3950 | 0.4030 | 0.4120 | 0.4210 | 0.4300 | 0.4390 | 0.4460 | 0.4530 | 0.4590 | 0.4650 | 0.4720 | 0.4780 | 0.4850 | 0.4910 | 0.4980 | 0.5040 | 0.510 | 0.5160 | 0.5220 | 0.526 |
| Guinea | Western Sub-Saharan Africa | Sub-Saharan Africa | 0.1750 | 0.1780 | 0.1820 | 0.1860 | 0.1900 | 0.1940 | 0.1990 | 0.2040 | 0.2090 | 0.2140 | 0.2190 | 0.2240 | 0.2290 | 0.2350 | 0.2390 | 0.2440 | 0.2490 | 0.2530 | 0.2580 | 0.2630 | 0.2670 | 0.2720 | 0.2780 | 0.2840 | 0.2900 | 0.2960 | 0.303 | 0.3100 | 0.3180 | 0.325 |
| Guinea-Bissau | Western Sub-Saharan Africa | Sub-Saharan Africa | 0.2000 | 0.2050 | 0.2090 | 0.2140 | 0.2190 | 0.2240 | 0.2300 | 0.2360 | 0.2390 | 0.2440 | 0.2490 | 0.2530 | 0.2570 | 0.2610 | 0.2660 | 0.2710 | 0.2750 | 0.2800 | 0.2860 | 0.2910 | 0.2970 | 0.3040 | 0.3100 | 0.3160 | 0.3220 | 0.3280 | 0.335 | 0.3420 | 0.3490 | 0.355 |
| Guyana | Caribbean | Latin America and Caribbean | 0.4520 | 0.4560 | 0.4610 | 0.4670 | 0.4740 | 0.4810 | 0.4880 | 0.4960 | 0.5030 | 0.5100 | 0.5160 | 0.5220 | 0.5270 | 0.5320 | 0.5360 | 0.5400 | 0.5440 | 0.5490 | 0.5540 | 0.5590 | 0.5650 | 0.5710 | 0.5770 | 0.5830 | 0.5900 | 0.5960 | 0.602 | 0.6080 | 0.6140 | 0.618 |
| Haiti | Caribbean | Latin America and Caribbean | 0.3070 | 0.3110 | 0.3140 | 0.3170 | 0.3200 | 0.3230 | 0.3280 | 0.3330 | 0.3380 | 0.3440 | 0.3500 | 0.3560 | 0.3610 | 0.3660 | 0.3710 | 0.3750 | 0.3790 | 0.3830 | 0.3870 | 0.3920 | 0.3950 | 0.3990 | 0.4030 | 0.4070 | 0.4120 | 0.4160 | 0.420 | 0.4240 | 0.4280 | 0.432 |
| Honduras | Central Latin America | Latin America and Caribbean | 0.3300 | 0.3350 | 0.3400 | 0.3450 | 0.3510 | 0.3570 | 0.3630 | 0.3700 | 0.3770 | 0.3840 | 0.3910 | 0.3970 | 0.4040 | 0.4110 | 0.4170 | 0.4240 | 0.4300 | 0.4370 | 0.4430 | 0.4480 | 0.4540 | 0.4590 | 0.4640 | 0.4690 | 0.4730 | 0.4780 | 0.482 | 0.4870 | 0.4920 | 0.496 |
| Hungary | Central Europe | Central Europe, eastern Europe, and central Asia | 0.6590 | 0.6630 | 0.6710 | 0.6780 | 0.6850 | 0.6930 | 0.7000 | 0.7070 | 0.7130 | 0.7180 | 0.7240 | 0.7300 | 0.7350 | 0.7410 | 0.7460 | 0.7510 | 0.7560 | 0.7600 | 0.7630 | 0.7680 | 0.7720 | 0.7730 | 0.7740 | 0.7740 | 0.7750 | 0.7780 | 0.781 | 0.7840 | 0.7880 | 0.791 |
| Iceland | Western Europe | High income | 0.7640 | 0.7700 | 0.7740 | 0.7780 | 0.7820 | 0.7850 | 0.7880 | 0.7900 | 0.7940 | 0.7990 | 0.8060 | 0.8130 | 0.8180 | 0.8220 | 0.8240 | 0.8270 | 0.8300 | 0.8340 | 0.8380 | 0.8420 | 0.8460 | 0.8470 | 0.8470 | 0.8480 | 0.8500 | 0.8540 | 0.858 | 0.8630 | 0.8660 | 0.869 |
| India | South Asia | South Asia | 0.3270 | 0.3330 | 0.3390 | 0.3450 | 0.3510 | 0.3580 | 0.3640 | 0.3710 | 0.3780 | 0.3860 | 0.3930 | 0.4000 | 0.4070 | 0.4140 | 0.4210 | 0.4290 | 0.4370 | 0.4460 | 0.4550 | 0.4630 | 0.4730 | 0.4830 | 0.4930 | 0.5040 | 0.5150 | 0.5260 | 0.537 | 0.5470 | 0.5580 | 0.566 |
| Indonesia | Southeast Asia | Southeast Asia, east Asia, and Oceania | 0.4520 | 0.4620 | 0.4720 | 0.4810 | 0.4900 | 0.4990 | 0.5080 | 0.5160 | 0.5220 | 0.5270 | 0.5330 | 0.5370 | 0.5420 | 0.5470 | 0.5520 | 0.5580 | 0.5640 | 0.5710 | 0.5780 | 0.5850 | 0.5930 | 0.6010 | 0.6090 | 0.6170 | 0.6250 | 0.6330 | 0.640 | 0.6470 | 0.6540 | 0.660 |
| Iran (Islamic Republic of) | North Africa and Middle East | North Africa and Middle East | 0.4040 | 0.4190 | 0.4330 | 0.4460 | 0.4570 | 0.4680 | 0.4770 | 0.4860 | 0.4960 | 0.5060 | 0.5170 | 0.5290 | 0.5410 | 0.5520 | 0.5620 | 0.5710 | 0.5810 | 0.5930 | 0.6030 | 0.6130 | 0.6220 | 0.6300 | 0.6350 | 0.6400 | 0.6450 | 0.6490 | 0.654 | 0.6590 | 0.6650 | 0.670 |
| Iraq | North Africa and Middle East | North Africa and Middle East | 0.3920 | 0.3970 | 0.4020 | 0.4070 | 0.4120 | 0.4170 | 0.4220 | 0.4290 | 0.4390 | 0.4510 | 0.4620 | 0.4750 | 0.4850 | 0.4930 | 0.5040 | 0.5140 | 0.5250 | 0.5350 | 0.5470 | 0.5580 | 0.5700 | 0.5830 | 0.5970 | 0.6100 | 0.6220 | 0.6320 | 0.644 | 0.6540 | 0.6630 | 0.671 |
| Ireland | Western Europe | High income | 0.7300 | 0.7350 | 0.7410 | 0.7470 | 0.7530 | 0.7580 | 0.7630 | 0.7680 | 0.7740 | 0.7800 | 0.7860 | 0.7930 | 0.7990 | 0.8060 | 0.8120 | 0.8160 | 0.8190 | 0.8210 | 0.8240 | 0.8270 | 0.8310 | 0.8350 | 0.8390 | 0.8420 | 0.8450 | 0.8500 | 0.854 | 0.8590 | 0.8640 | 0.867 |
| Israel | Western Europe | High income | 0.7170 | 0.7210 | 0.7260 | 0.7300 | 0.7340 | 0.7380 | 0.7420 | 0.7450 | 0.7490 | 0.7520 | 0.7560 | 0.7600 | 0.7620 | 0.7650 | 0.7690 | 0.7730 | 0.7760 | 0.7780 | 0.7780 | 0.7790 | 0.7810 | 0.7840 | 0.7870 | 0.7900 | 0.7920 | 0.7940 | 0.796 | 0.7980 | 0.8000 | 0.803 |
| Italy | Western Europe | High income | 0.7120 | 0.7170 | 0.7220 | 0.7270 | 0.7320 | 0.7370 | 0.7400 | 0.7440 | 0.7470 | 0.7500 | 0.7530 | 0.7570 | 0.7610 | 0.7630 | 0.7660 | 0.7680 | 0.7710 | 0.7730 | 0.7750 | 0.7770 | 0.7800 | 0.7820 | 0.7840 | 0.7870 | 0.7890 | 0.7910 | 0.794 | 0.7960 | 0.7980 | 0.801 |
| Jamaica | Caribbean | Latin America and Caribbean | 0.5420 | 0.5470 | 0.5530 | 0.5600 | 0.5660 | 0.5730 | 0.5800 | 0.5860 | 0.5920 | 0.5980 | 0.6030 | 0.6090 | 0.6140 | 0.6200 | 0.6250 | 0.6300 | 0.6350 | 0.6400 | 0.6450 | 0.6490 | 0.6530 | 0.6570 | 0.6610 | 0.6640 | 0.6680 | 0.6710 | 0.675 | 0.6780 | 0.6810 | 0.684 |
| Japan | High-income Asia Pacific | High income | 0.7910 | 0.7960 | 0.8010 | 0.8050 | 0.8090 | 0.8130 | 0.8170 | 0.8200 | 0.8220 | 0.8240 | 0.8260 | 0.8280 | 0.8300 | 0.8330 | 0.8360 | 0.8380 | 0.8400 | 0.8420 | 0.8440 | 0.8460 | 0.8480 | 0.8500 | 0.8530 | 0.8550 | 0.8570 | 0.8600 | 0.862 | 0.8650 | 0.8670 | 0.870 |
| Jordan | North Africa and Middle East | North Africa and Middle East | 0.5200 | 0.5290 | 0.5370 | 0.5460 | 0.5540 | 0.5620 | 0.5700 | 0.5770 | 0.5850 | 0.5920 | 0.6000 | 0.6070 | 0.6140 | 0.6210 | 0.6300 | 0.6390 | 0.6480 | 0.6560 | 0.6640 | 0.6730 | 0.6810 | 0.6880 | 0.6950 | 0.7020 | 0.7070 | 0.7130 | 0.718 | 0.7230 | 0.7270 | 0.731 |
| Kazakhstan | Central Asia | Central Europe, eastern Europe, and central Asia | 0.6020 | 0.6060 | 0.6110 | 0.6150 | 0.6190 | 0.6220 | 0.6250 | 0.6280 | 0.6300 | 0.6320 | 0.6350 | 0.6390 | 0.6440 | 0.6490 | 0.6550 | 0.6610 | 0.6670 | 0.6740 | 0.6790 | 0.6830 | 0.6880 | 0.6920 | 0.6960 | 0.7000 | 0.7040 | 0.7080 | 0.712 | 0.7160 | 0.7200 | 0.723 |
| Kenya | Eastern Sub-Saharan Africa | Sub-Saharan Africa | 0.3330 | 0.3410 | 0.3480 | 0.3540 | 0.3600 | 0.3660 | 0.3720 | 0.3780 | 0.3830 | 0.3880 | 0.3920 | 0.3970 | 0.4010 | 0.4040 | 0.4090 | 0.4130 | 0.4180 | 0.4240 | 0.4290 | 0.4350 | 0.4410 | 0.4480 | 0.4550 | 0.4630 | 0.4700 | 0.4780 | 0.486 | 0.4940 | 0.5020 | 0.508 |
| Kiribati | Oceania | Southeast Asia, east Asia, and Oceania | 0.4250 | 0.4270 | 0.4290 | 0.4320 | 0.4350 | 0.4380 | 0.4410 | 0.4440 | 0.4470 | 0.4510 | 0.4550 | 0.4590 | 0.4630 | 0.4680 | 0.4720 | 0.4760 | 0.4800 | 0.4840 | 0.4870 | 0.4900 | 0.4920 | 0.4950 | 0.4970 | 0.5010 | 0.5040 | 0.5090 | 0.514 | 0.5180 | 0.5230 | 0.527 |
| Kuwait | North Africa and Middle East | North Africa and Middle East | 0.6550 | 0.6590 | 0.6620 | 0.6670 | 0.6730 | 0.6800 | 0.6890 | 0.6990 | 0.7090 | 0.7170 | 0.7240 | 0.7290 | 0.7350 | 0.7420 | 0.7500 | 0.7600 | 0.7690 | 0.7770 | 0.7850 | 0.7930 | 0.8010 | 0.8080 | 0.8150 | 0.8220 | 0.8280 | 0.8340 | 0.839 | 0.8440 | 0.8480 | 0.851 |
| Kyrgyzstan | Central Asia | Central Europe, eastern Europe, and central Asia | 0.5320 | 0.5370 | 0.5410 | 0.5430 | 0.5420 | 0.5410 | 0.5390 | 0.5370 | 0.5360 | 0.5340 | 0.5340 | 0.5350 | 0.5370 | 0.5400 | 0.5440 | 0.5460 | 0.5490 | 0.5520 | 0.5550 | 0.5580 | 0.5600 | 0.5630 | 0.5650 | 0.5690 | 0.5740 | 0.5780 | 0.583 | 0.5880 | 0.5920 | 0.596 |
| Lao People's Democratic Republic | Southeast Asia | Southeast Asia, east Asia, and Oceania | 0.2680 | 0.2740 | 0.2790 | 0.2850 | 0.2900 | 0.2960 | 0.3020 | 0.3090 | 0.3150 | 0.3220 | 0.3290 | 0.3360 | 0.3440 | 0.3510 | 0.3590 | 0.3670 | 0.3760 | 0.3850 | 0.3940 | 0.4030 | 0.4130 | 0.4220 | 0.4310 | 0.4410 | 0.4500 | 0.4580 | 0.467 | 0.4750 | 0.4830 | 0.490 |
| Latvia | Eastern Europe | Central Europe, eastern Europe, and central Asia | 0.6750 | 0.6820 | 0.6910 | 0.7000 | 0.7080 | 0.7130 | 0.7160 | 0.7190 | 0.7210 | 0.7230 | 0.7270 | 0.7330 | 0.7390 | 0.7450 | 0.7530 | 0.7600 | 0.7660 | 0.7740 | 0.7840 | 0.7930 | 0.7970 | 0.7980 | 0.8010 | 0.8030 | 0.8040 | 0.8050 | 0.809 | 0.8130 | 0.8170 | 0.820 |
| Lebanon | North Africa and Middle East | North Africa and Middle East | 0.4620 | 0.4700 | 0.4770 | 0.4850 | 0.4930 | 0.5020 | 0.5110 | 0.5200 | 0.5300 | 0.5400 | 0.5480 | 0.5570 | 0.5650 | 0.5740 | 0.5820 | 0.5910 | 0.6000 | 0.6090 | 0.6180 | 0.6280 | 0.6390 | 0.6490 | 0.6600 | 0.6700 | 0.6770 | 0.6850 | 0.691 | 0.6980 | 0.7040 | 0.708 |
| Lesotho | Southern Sub-Saharan Africa | Sub-Saharan Africa | 0.3210 | 0.3270 | 0.3330 | 0.3400 | 0.3460 | 0.3530 | 0.3600 | 0.3670 | 0.3730 | 0.3800 | 0.3870 | 0.3930 | 0.3990 | 0.4050 | 0.4110 | 0.4170 | 0.4230 | 0.4290 | 0.4350 | 0.4410 | 0.4480 | 0.4550 | 0.4620 | 0.4690 | 0.4760 | 0.4830 | 0.489 | 0.4960 | 0.5020 | 0.507 |
| Liberia | Western Sub-Saharan Africa | Sub-Saharan Africa | 0.2210 | 0.2220 | 0.2190 | 0.2140 | 0.2060 | 0.1960 | 0.1830 | 0.1760 | 0.1750 | 0.1840 | 0.2030 | 0.2200 | 0.2380 | 0.2450 | 0.2520 | 0.2580 | 0.2650 | 0.2720 | 0.2790 | 0.2870 | 0.2960 | 0.3050 | 0.3140 | 0.3250 | 0.3350 | 0.3440 | 0.351 | 0.3580 | 0.3650 | 0.370 |
| Libya | North Africa and Middle East | North Africa and Middle East | 0.4050 | 0.4220 | 0.4380 | 0.4550 | 0.4720 | 0.4890 | 0.5060 | 0.5220 | 0.5380 | 0.5520 | 0.5660 | 0.5800 | 0.5930 | 0.6060 | 0.6190 | 0.6320 | 0.6450 | 0.6580 | 0.6700 | 0.6810 | 0.6910 | 0.6950 | 0.7030 | 0.7070 | 0.7070 | 0.7070 | 0.705 | 0.7050 | 0.7070 | 0.709 |
| Lithuania | Eastern Europe | Central Europe, eastern Europe, and central Asia | 0.6700 | 0.6720 | 0.6820 | 0.6910 | 0.6940 | 0.6960 | 0.7000 | 0.7050 | 0.7090 | 0.7140 | 0.7230 | 0.7300 | 0.7360 | 0.7430 | 0.7520 | 0.7600 | 0.7650 | 0.7710 | 0.7820 | 0.7920 | 0.7970 | 0.8010 | 0.8080 | 0.8130 | 0.8170 | 0.8220 | 0.829 | 0.8350 | 0.8390 | 0.843 |
| Luxembourg | Western Europe | High income | 0.8150 | 0.8180 | 0.8200 | 0.8230 | 0.8280 | 0.8330 | 0.8360 | 0.8390 | 0.8420 | 0.8440 | 0.8470 | 0.8500 | 0.8530 | 0.8550 | 0.8570 | 0.8580 | 0.8620 | 0.8660 | 0.8690 | 0.8710 | 0.8720 | 0.8740 | 0.8770 | 0.8800 | 0.8830 | 0.8860 | 0.889 | 0.8920 | 0.8940 | 0.895 |
| Madagascar | Eastern Sub-Saharan Africa | Sub-Saharan Africa | 0.2650 | 0.2680 | 0.2700 | 0.2730 | 0.2750 | 0.2770 | 0.2800 | 0.2830 | 0.2860 | 0.2900 | 0.2940 | 0.2990 | 0.3020 | 0.3070 | 0.3110 | 0.3160 | 0.3200 | 0.3240 | 0.3280 | 0.3320 | 0.3360 | 0.3420 | 0.3480 | 0.3550 | 0.3610 | 0.3690 | 0.376 | 0.3830 | 0.3910 | 0.396 |
| Malawi | Eastern Sub-Saharan Africa | Sub-Saharan Africa | 0.2130 | 0.2150 | 0.2150 | 0.2170 | 0.2180 | 0.2200 | 0.2250 | 0.2300 | 0.2350 | 0.2400 | 0.2450 | 0.2490 | 0.2540 | 0.2600 | 0.2670 | 0.2740 | 0.2810 | 0.2900 | 0.2990 | 0.3080 | 0.3170 | 0.3260 | 0.3350 | 0.3420 | 0.3500 | 0.3580 | 0.365 | 0.3720 | 0.3790 | 0.384 |
| Malaysia | Southeast Asia | Southeast Asia, east Asia, and Oceania | 0.5420 | 0.5480 | 0.5540 | 0.5620 | 0.5720 | 0.5810 | 0.5900 | 0.6000 | 0.6110 | 0.6220 | 0.6300 | 0.6380 | 0.6460 | 0.6520 | 0.6590 | 0.6650 | 0.6710 | 0.6770 | 0.6810 | 0.6870 | 0.6930 | 0.6980 | 0.7040 | 0.7100 | 0.7160 | 0.7220 | 0.726 | 0.7280 | 0.7320 | 0.737 |
| Maldives | Southeast Asia | Southeast Asia, east Asia, and Oceania | 0.3030 | 0.3140 | 0.3240 | 0.3360 | 0.3470 | 0.3590 | 0.3700 | 0.3820 | 0.3940 | 0.4060 | 0.4170 | 0.4270 | 0.4370 | 0.4470 | 0.4560 | 0.4640 | 0.4730 | 0.4810 | 0.4900 | 0.4970 | 0.5040 | 0.5110 | 0.5180 | 0.5250 | 0.5320 | 0.5380 | 0.544 | 0.5510 | 0.5570 | 0.562 |
| Mali | Western Sub-Saharan Africa | Sub-Saharan Africa | 0.1260 | 0.1290 | 0.1320 | 0.1360 | 0.1390 | 0.1430 | 0.1470 | 0.1510 | 0.1550 | 0.1590 | 0.1630 | 0.1680 | 0.1730 | 0.1780 | 0.1830 | 0.1880 | 0.1930 | 0.1980 | 0.2030 | 0.2090 | 0.2140 | 0.2200 | 0.2250 | 0.2300 | 0.2350 | 0.2410 | 0.247 | 0.2530 | 0.2590 | 0.263 |
| Malta | Western Europe | High income | 0.6660 | 0.6700 | 0.6750 | 0.6820 | 0.6900 | 0.6950 | 0.6960 | 0.7000 | 0.7080 | 0.7150 | 0.7220 | 0.7290 | 0.7330 | 0.7370 | 0.7410 | 0.7450 | 0.7490 | 0.7530 | 0.7570 | 0.7610 | 0.7640 | 0.7680 | 0.7720 | 0.7750 | 0.7790 | 0.7840 | 0.788 | 0.7930 | 0.7970 | 0.801 |
| Marshall Islands | Oceania | Southeast Asia, east Asia, and Oceania | 0.3980 | 0.4040 | 0.4100 | 0.4160 | 0.4230 | 0.4300 | 0.4350 | 0.4390 | 0.4420 | 0.4460 | 0.4500 | 0.4540 | 0.4580 | 0.4630 | 0.4670 | 0.4720 | 0.4770 | 0.4820 | 0.4870 | 0.4930 | 0.4980 | 0.5040 | 0.5090 | 0.5150 | 0.5200 | 0.5250 | 0.531 | 0.5360 | 0.5410 | 0.544 |
| Mauritania | Western Sub-Saharan Africa | Sub-Saharan Africa | 0.3080 | 0.3140 | 0.3190 | 0.3260 | 0.3320 | 0.3380 | 0.3440 | 0.3490 | 0.3550 | 0.3600 | 0.3650 | 0.3690 | 0.3740 | 0.3790 | 0.3840 | 0.3900 | 0.3980 | 0.4060 | 0.4130 | 0.4200 | 0.4270 | 0.4350 | 0.4430 | 0.4500 | 0.4590 | 0.4670 | 0.474 | 0.4820 | 0.4900 | 0.496 |
| Mauritius | Southeast Asia | Southeast Asia, east Asia, and Oceania | 0.5270 | 0.5320 | 0.5350 | 0.5430 | 0.5560 | 0.5650 | 0.5700 | 0.5760 | 0.5830 | 0.5880 | 0.5930 | 0.5980 | 0.6030 | 0.6080 | 0.6140 | 0.6210 | 0.6270 | 0.6330 | 0.6400 | 0.6460 | 0.6520 | 0.6580 | 0.6650 | 0.6730 | 0.6800 | 0.6860 | 0.690 | 0.6950 | 0.7000 | 0.705 |
| Mexico | Central Latin America | Latin America and Caribbean | 0.5070 | 0.5140 | 0.5200 | 0.5260 | 0.5320 | 0.5370 | 0.5420 | 0.5470 | 0.5530 | 0.5580 | 0.5630 | 0.5690 | 0.5740 | 0.5780 | 0.5830 | 0.5880 | 0.5920 | 0.5970 | 0.6010 | 0.6050 | 0.6080 | 0.6130 | 0.6170 | 0.6210 | 0.6260 | 0.6310 | 0.636 | 0.6400 | 0.6450 | 0.649 |
| Micronesia (Federated States of) | Oceania | Southeast Asia, east Asia, and Oceania | 0.4470 | 0.4530 | 0.4590 | 0.4650 | 0.4710 | 0.4780 | 0.4830 | 0.4880 | 0.4920 | 0.4970 | 0.5020 | 0.5070 | 0.5110 | 0.5160 | 0.5210 | 0.5250 | 0.5300 | 0.5340 | 0.5380 | 0.5420 | 0.5460 | 0.5500 | 0.5540 | 0.5580 | 0.5610 | 0.5650 | 0.569 | 0.5730 | 0.5770 | 0.580 |
| Monaco | Western Europe | High income | 0.8340 | 0.8370 | 0.8400 | 0.8430 | 0.8460 | 0.8490 | 0.8520 | 0.8550 | 0.8570 | 0.8600 | 0.8620 | 0.8650 | 0.8670 | 0.8700 | 0.8720 | 0.8750 | 0.8770 | 0.8790 | 0.8810 | 0.8830 | 0.8860 | 0.8880 | 0.8900 | 0.8920 | 0.8930 | 0.8950 | 0.897 | 0.8990 | 0.9010 | 0.902 |
| Mongolia | Central Asia | Central Europe, eastern Europe, and central Asia | 0.4650 | 0.4700 | 0.4750 | 0.4800 | 0.4840 | 0.4900 | 0.4950 | 0.5010 | 0.5060 | 0.5120 | 0.5170 | 0.5230 | 0.5280 | 0.5340 | 0.5390 | 0.5450 | 0.5500 | 0.5550 | 0.5600 | 0.5630 | 0.5660 | 0.5700 | 0.5750 | 0.5790 | 0.5840 | 0.5880 | 0.592 | 0.5970 | 0.6010 | 0.606 |
| Montenegro | Central Europe | Central Europe, eastern Europe, and central Asia | 0.7010 | 0.7010 | 0.6990 | 0.6950 | 0.6900 | 0.6870 | 0.6860 | 0.6870 | 0.6900 | 0.6920 | 0.6960 | 0.7010 | 0.7060 | 0.7120 | 0.7170 | 0.7230 | 0.7290 | 0.7360 | 0.7430 | 0.7490 | 0.7540 | 0.7590 | 0.7640 | 0.7680 | 0.7730 | 0.7770 | 0.780 | 0.7840 | 0.7880 | 0.791 |
| Morocco | North Africa and Middle East | North Africa and Middle East | 0.3470 | 0.3540 | 0.3610 | 0.3670 | 0.3740 | 0.3800 | 0.3860 | 0.3920 | 0.3980 | 0.4030 | 0.4090 | 0.4140 | 0.4200 | 0.4260 | 0.4320 | 0.4390 | 0.4450 | 0.4520 | 0.4600 | 0.4670 | 0.4750 | 0.4830 | 0.4910 | 0.4990 | 0.5080 | 0.5160 | 0.524 | 0.5330 | 0.5410 | 0.548 |
| Mozambique | Eastern Sub-Saharan Africa | Sub-Saharan Africa | 0.1200 | 0.1220 | 0.1230 | 0.1260 | 0.1290 | 0.1310 | 0.1370 | 0.1440 | 0.1520 | 0.1590 | 0.1650 | 0.1720 | 0.1790 | 0.1860 | 0.1940 | 0.2010 | 0.2080 | 0.2150 | 0.2230 | 0.2300 | 0.2370 | 0.2440 | 0.2520 | 0.2600 | 0.2680 | 0.2770 | 0.285 | 0.2940 | 0.3010 | 0.307 |
| Myanmar | Southeast Asia | Southeast Asia, east Asia, and Oceania | 0.2840 | 0.2870 | 0.2900 | 0.2950 | 0.3000 | 0.3060 | 0.3130 | 0.3200 | 0.3270 | 0.3350 | 0.3440 | 0.3530 | 0.3630 | 0.3730 | 0.3840 | 0.3950 | 0.4060 | 0.4170 | 0.4270 | 0.4370 | 0.4460 | 0.4550 | 0.4640 | 0.4730 | 0.4820 | 0.4900 | 0.498 | 0.5060 | 0.5140 | 0.521 |
| Namibia | Southern Sub-Saharan Africa | Sub-Saharan Africa | 0.4540 | 0.4590 | 0.4650 | 0.4700 | 0.4750 | 0.4800 | 0.4860 | 0.4910 | 0.4950 | 0.5000 | 0.5050 | 0.5090 | 0.5140 | 0.5180 | 0.5230 | 0.5290 | 0.5340 | 0.5400 | 0.5460 | 0.5520 | 0.5580 | 0.5640 | 0.5710 | 0.5770 | 0.5840 | 0.5910 | 0.597 | 0.6030 | 0.6080 | 0.612 |
| Nauru | Oceania | Southeast Asia, east Asia, and Oceania | 0.4990 | 0.5010 | 0.5030 | 0.5040 | 0.5040 | 0.5050 | 0.5050 | 0.5040 | 0.5040 | 0.5030 | 0.5030 | 0.5030 | 0.5030 | 0.5030 | 0.5040 | 0.5060 | 0.5090 | 0.5100 | 0.5150 | 0.5210 | 0.5290 | 0.5380 | 0.5470 | 0.5590 | 0.5730 | 0.5850 | 0.595 | 0.6050 | 0.6130 | 0.618 |
| Nepal | South Asia | South Asia | 0.1980 | 0.2030 | 0.2080 | 0.2150 | 0.2210 | 0.2280 | 0.2360 | 0.2440 | 0.2510 | 0.2590 | 0.2670 | 0.2760 | 0.2840 | 0.2910 | 0.2990 | 0.3070 | 0.3150 | 0.3220 | 0.3300 | 0.3390 | 0.3470 | 0.3560 | 0.3650 | 0.3730 | 0.3820 | 0.3910 | 0.399 | 0.4080 | 0.4160 | 0.422 |
| Netherlands | Western Europe | High income | 0.7960 | 0.8010 | 0.8060 | 0.8100 | 0.8140 | 0.8180 | 0.8210 | 0.8240 | 0.8270 | 0.8300 | 0.8320 | 0.8360 | 0.8390 | 0.8420 | 0.8450 | 0.8480 | 0.8510 | 0.8530 | 0.8560 | 0.8580 | 0.8610 | 0.8640 | 0.8660 | 0.8690 | 0.8710 | 0.8740 | 0.876 | 0.8780 | 0.8810 | 0.883 |
| New Zealand | Australasia | High income | 0.7570 | 0.7620 | 0.7650 | 0.7690 | 0.7720 | 0.7740 | 0.7780 | 0.7820 | 0.7850 | 0.7870 | 0.7900 | 0.7940 | 0.7960 | 0.7980 | 0.8020 | 0.8030 | 0.8000 | 0.8000 | 0.8030 | 0.8070 | 0.8090 | 0.8120 | 0.8160 | 0.8210 | 0.8250 | 0.8280 | 0.832 | 0.8350 | 0.8380 | 0.840 |
| Nicaragua | Central Latin America | Latin America and Caribbean | 0.3380 | 0.3450 | 0.3530 | 0.3600 | 0.3680 | 0.3760 | 0.3850 | 0.3930 | 0.4020 | 0.4110 | 0.4190 | 0.4260 | 0.4330 | 0.4380 | 0.4440 | 0.4490 | 0.4530 | 0.4580 | 0.4620 | 0.4660 | 0.4700 | 0.4740 | 0.4790 | 0.4840 | 0.4890 | 0.4950 | 0.500 | 0.5060 | 0.5120 | 0.517 |
| Niger | Western Sub-Saharan Africa | Sub-Saharan Africa | 0.0728 | 0.0746 | 0.0761 | 0.0777 | 0.0793 | 0.0808 | 0.0822 | 0.0836 | 0.0853 | 0.0871 | 0.0887 | 0.0907 | 0.0930 | 0.0956 | 0.0982 | 0.1010 | 0.1040 | 0.1080 | 0.1110 | 0.1150 | 0.1190 | 0.1230 | 0.1280 | 0.1330 | 0.1380 | 0.1430 | 0.148 | 0.1530 | 0.1580 | 0.162 |
| Nigeria | Western Sub-Saharan Africa | Sub-Saharan Africa | 0.3050 | 0.3080 | 0.3120 | 0.3150 | 0.3190 | 0.3240 | 0.3290 | 0.3340 | 0.3390 | 0.3440 | 0.3500 | 0.3560 | 0.3630 | 0.3710 | 0.3810 | 0.3920 | 0.4020 | 0.4120 | 0.4220 | 0.4320 | 0.4420 | 0.4510 | 0.4600 | 0.4690 | 0.4780 | 0.4870 | 0.495 | 0.5030 | 0.5100 | 0.515 |
| Niue | Oceania | Southeast Asia, east Asia, and Oceania | 0.5660 | 0.5710 | 0.5760 | 0.5810 | 0.5860 | 0.5910 | 0.5950 | 0.6000 | 0.6050 | 0.6090 | 0.6140 | 0.6180 | 0.6220 | 0.6260 | 0.6310 | 0.6380 | 0.6450 | 0.6510 | 0.6570 | 0.6630 | 0.6690 | 0.6750 | 0.6810 | 0.6850 | 0.6900 | 0.6950 | 0.699 | 0.7030 | 0.7070 | 0.711 |
| North Macedonia | Central Europe | Central Europe, eastern Europe, and central Asia | 0.6180 | 0.6200 | 0.6230 | 0.6250 | 0.6270 | 0.6310 | 0.6350 | 0.6400 | 0.6460 | 0.6510 | 0.6560 | 0.6620 | 0.6680 | 0.6740 | 0.6790 | 0.6840 | 0.6890 | 0.6940 | 0.7000 | 0.7040 | 0.7090 | 0.7130 | 0.7170 | 0.7220 | 0.7260 | 0.7300 | 0.734 | 0.7380 | 0.7410 | 0.744 |
| Northern Mariana Islands | Oceania | Southeast Asia, east Asia, and Oceania | 0.6920 | 0.6980 | 0.7040 | 0.7090 | 0.7140 | 0.7180 | 0.7220 | 0.7250 | 0.7270 | 0.7310 | 0.7380 | 0.7440 | 0.7480 | 0.7500 | 0.7510 | 0.7520 | 0.7520 | 0.7530 | 0.7530 | 0.7520 | 0.7510 | 0.7500 | 0.7500 | 0.7500 | 0.7500 | 0.7510 | 0.756 | 0.7610 | 0.7670 | 0.771 |
| Norway | Western Europe | High income | 0.8070 | 0.8120 | 0.8180 | 0.8230 | 0.8280 | 0.8320 | 0.8370 | 0.8430 | 0.8470 | 0.8510 | 0.8560 | 0.8610 | 0.8660 | 0.8690 | 0.8720 | 0.8740 | 0.8760 | 0.8770 | 0.8790 | 0.8810 | 0.8850 | 0.8890 | 0.8930 | 0.8960 | 0.9000 | 0.9030 | 0.907 | 0.9100 | 0.9120 | 0.913 |
| Oman | North Africa and Middle East | North Africa and Middle East | 0.4410 | 0.4550 | 0.4690 | 0.4850 | 0.5000 | 0.5140 | 0.5280 | 0.5430 | 0.5580 | 0.5730 | 0.5880 | 0.6010 | 0.6150 | 0.6280 | 0.6410 | 0.6530 | 0.6640 | 0.6780 | 0.6920 | 0.7040 | 0.7150 | 0.7260 | 0.7370 | 0.7470 | 0.7540 | 0.7600 | 0.767 | 0.7730 | 0.7780 | 0.783 |
| Pakistan | South Asia | South Asia | 0.2470 | 0.2530 | 0.2590 | 0.2650 | 0.2710 | 0.2770 | 0.2830 | 0.2890 | 0.2950 | 0.3010 | 0.3070 | 0.3130 | 0.3200 | 0.3260 | 0.3330 | 0.3400 | 0.3470 | 0.3550 | 0.3630 | 0.3710 | 0.3790 | 0.3870 | 0.3940 | 0.4020 | 0.4100 | 0.4180 | 0.426 | 0.4340 | 0.4420 | 0.449 |
| Palau | Oceania | Southeast Asia, east Asia, and Oceania | 0.6210 | 0.6290 | 0.6360 | 0.6420 | 0.6470 | 0.6520 | 0.6580 | 0.6630 | 0.6680 | 0.6720 | 0.6760 | 0.6800 | 0.6830 | 0.6860 | 0.6900 | 0.6930 | 0.6970 | 0.7000 | 0.7030 | 0.7050 | 0.7070 | 0.7100 | 0.7140 | 0.7170 | 0.7200 | 0.7250 | 0.729 | 0.7320 | 0.7350 | 0.738 |
| Palestine | North Africa and Middle East | North Africa and Middle East | 0.3140 | 0.3200 | 0.3290 | 0.3370 | 0.3470 | 0.3560 | 0.3650 | 0.3750 | 0.3860 | 0.3970 | 0.4070 | 0.4150 | 0.4220 | 0.4300 | 0.4390 | 0.4490 | 0.4580 | 0.4670 | 0.4760 | 0.4860 | 0.4970 | 0.5090 | 0.5210 | 0.5330 | 0.5430 | 0.5530 | 0.564 | 0.5730 | 0.5820 | 0.588 |
| Panama | Central Latin America | Latin America and Caribbean | 0.5440 | 0.5490 | 0.5550 | 0.5590 | 0.5620 | 0.5650 | 0.5680 | 0.5730 | 0.5790 | 0.5860 | 0.5920 | 0.5970 | 0.6020 | 0.6050 | 0.6070 | 0.6100 | 0.6130 | 0.6160 | 0.6200 | 0.6230 | 0.6270 | 0.6300 | 0.6350 | 0.6420 | 0.6500 | 0.6580 | 0.666 | 0.6740 | 0.6800 | 0.686 |
| Papua New Guinea | Oceania | Southeast Asia, east Asia, and Oceania | 0.2920 | 0.2950 | 0.2990 | 0.3040 | 0.3090 | 0.3130 | 0.3170 | 0.3200 | 0.3230 | 0.3260 | 0.3290 | 0.3310 | 0.3330 | 0.3350 | 0.3370 | 0.3390 | 0.3420 | 0.3450 | 0.3480 | 0.3520 | 0.3560 | 0.3600 | 0.3630 | 0.3670 | 0.3720 | 0.3770 | 0.382 | 0.3860 | 0.3910 | 0.394 |
| Paraguay | Tropical Latin America | Latin America and Caribbean | 0.4650 | 0.4710 | 0.4770 | 0.4840 | 0.4910 | 0.4980 | 0.5050 | 0.5120 | 0.5190 | 0.5240 | 0.5290 | 0.5340 | 0.5390 | 0.5440 | 0.5490 | 0.5540 | 0.5590 | 0.5640 | 0.5700 | 0.5750 | 0.5820 | 0.5880 | 0.5940 | 0.6010 | 0.6080 | 0.6150 | 0.621 | 0.6270 | 0.6330 | 0.638 |
| Peru | Andean Latin America | Latin America and Caribbean | 0.5010 | 0.5050 | 0.5080 | 0.5110 | 0.5160 | 0.5210 | 0.5260 | 0.5320 | 0.5370 | 0.5430 | 0.5480 | 0.5520 | 0.5570 | 0.5620 | 0.5660 | 0.5710 | 0.5770 | 0.5830 | 0.5890 | 0.5950 | 0.6010 | 0.6080 | 0.6140 | 0.6200 | 0.6250 | 0.6300 | 0.635 | 0.6400 | 0.6450 | 0.648 |
| Philippines | Southeast Asia | Southeast Asia, east Asia, and Oceania | 0.4970 | 0.5010 | 0.5050 | 0.5090 | 0.5130 | 0.5180 | 0.5220 | 0.5260 | 0.5290 | 0.5320 | 0.5340 | 0.5370 | 0.5400 | 0.5420 | 0.5450 | 0.5470 | 0.5500 | 0.5540 | 0.5580 | 0.5620 | 0.5670 | 0.5720 | 0.5770 | 0.5830 | 0.5890 | 0.5960 | 0.603 | 0.6100 | 0.6170 | 0.623 |
| Poland | Central Europe | Central Europe, eastern Europe, and central Asia | 0.6320 | 0.6370 | 0.6440 | 0.6530 | 0.6610 | 0.6700 | 0.6770 | 0.6850 | 0.6930 | 0.7010 | 0.7090 | 0.7170 | 0.7240 | 0.7300 | 0.7350 | 0.7400 | 0.7430 | 0.7470 | 0.7520 | 0.7570 | 0.7630 | 0.7700 | 0.7750 | 0.7800 | 0.7840 | 0.7880 | 0.791 | 0.7950 | 0.7980 | 0.802 |
| Portugal | Western Europe | High income | 0.6070 | 0.6150 | 0.6220 | 0.6290 | 0.6360 | 0.6410 | 0.6470 | 0.6510 | 0.6560 | 0.6610 | 0.6660 | 0.6710 | 0.6760 | 0.6810 | 0.6860 | 0.6900 | 0.6940 | 0.6980 | 0.7010 | 0.7050 | 0.7090 | 0.7140 | 0.7180 | 0.7220 | 0.7260 | 0.7290 | 0.732 | 0.7360 | 0.7390 | 0.743 |
| Puerto Rico | Caribbean | Latin America and Caribbean | 0.6700 | 0.6760 | 0.6790 | 0.6840 | 0.6890 | 0.6930 | 0.6990 | 0.7050 | 0.7110 | 0.7140 | 0.7200 | 0.7310 | 0.7380 | 0.7420 | 0.7440 | 0.7480 | 0.7520 | 0.7560 | 0.7600 | 0.7640 | 0.7690 | 0.7740 | 0.7790 | 0.7850 | 0.7930 | 0.8020 | 0.808 | 0.8110 | 0.8130 | 0.814 |
| Qatar | North Africa and Middle East | North Africa and Middle East | 0.5850 | 0.5980 | 0.6100 | 0.6210 | 0.6320 | 0.6430 | 0.6540 | 0.6660 | 0.6760 | 0.6850 | 0.6940 | 0.7030 | 0.7110 | 0.7190 | 0.7270 | 0.7350 | 0.7430 | 0.7500 | 0.7570 | 0.7640 | 0.7720 | 0.7790 | 0.7860 | 0.7930 | 0.7990 | 0.8060 | 0.812 | 0.8180 | 0.8250 | 0.830 |
| Republic of Korea | High-income Asia Pacific | High income | 0.6860 | 0.6970 | 0.7080 | 0.7190 | 0.7300 | 0.7400 | 0.7500 | 0.7600 | 0.7670 | 0.7760 | 0.7840 | 0.7910 | 0.7990 | 0.8050 | 0.8110 | 0.8170 | 0.8230 | 0.8280 | 0.8330 | 0.8370 | 0.8420 | 0.8460 | 0.8510 | 0.8550 | 0.8590 | 0.8630 | 0.867 | 0.8710 | 0.8750 | 0.878 |
| Republic of Moldova | Eastern Europe | Central Europe, eastern Europe, and central Asia | 0.5850 | 0.5890 | 0.5910 | 0.5940 | 0.5940 | 0.5950 | 0.5940 | 0.5930 | 0.5910 | 0.5870 | 0.5850 | 0.5850 | 0.5880 | 0.5930 | 0.6000 | 0.6070 | 0.6150 | 0.6220 | 0.6300 | 0.6370 | 0.6440 | 0.6510 | 0.6580 | 0.6650 | 0.6720 | 0.6770 | 0.683 | 0.6880 | 0.6930 | 0.696 |
| Romania | Central Europe | Central Europe, eastern Europe, and central Asia | 0.6250 | 0.6320 | 0.6350 | 0.6380 | 0.6430 | 0.6490 | 0.6530 | 0.6550 | 0.6590 | 0.6640 | 0.6690 | 0.6770 | 0.6820 | 0.6860 | 0.6930 | 0.6980 | 0.7020 | 0.7070 | 0.7110 | 0.7180 | 0.7260 | 0.7290 | 0.7340 | 0.7400 | 0.7410 | 0.7440 | 0.747 | 0.7520 | 0.7560 | 0.760 |
| Russian Federation | Eastern Europe | Central Europe, eastern Europe, and central Asia | 0.6950 | 0.7030 | 0.7160 | 0.7200 | 0.7190 | 0.7220 | 0.7240 | 0.7250 | 0.7260 | 0.7280 | 0.7280 | 0.7280 | 0.7300 | 0.7340 | 0.7410 | 0.7490 | 0.7540 | 0.7590 | 0.7640 | 0.7700 | 0.7750 | 0.7770 | 0.7790 | 0.7840 | 0.7880 | 0.7930 | 0.797 | 0.8010 | 0.8030 | 0.805 |
| Rwanda | Eastern Sub-Saharan Africa | Sub-Saharan Africa | 0.2570 | 0.2590 | 0.2620 | 0.2650 | 0.2610 | 0.2610 | 0.2620 | 0.2650 | 0.2690 | 0.2720 | 0.2770 | 0.2830 | 0.2910 | 0.2980 | 0.3070 | 0.3150 | 0.3250 | 0.3330 | 0.3420 | 0.3510 | 0.3590 | 0.3680 | 0.3760 | 0.3840 | 0.3910 | 0.3990 | 0.407 | 0.4150 | 0.4220 | 0.429 |
| Saint Kitts and Nevis | Caribbean | Latin America and Caribbean | 0.5830 | 0.5920 | 0.5990 | 0.6070 | 0.6140 | 0.6200 | 0.6260 | 0.6330 | 0.6380 | 0.6440 | 0.6500 | 0.6570 | 0.6630 | 0.6680 | 0.6730 | 0.6790 | 0.6850 | 0.6900 | 0.6960 | 0.7010 | 0.7060 | 0.7100 | 0.7140 | 0.7190 | 0.7240 | 0.7280 | 0.733 | 0.7380 | 0.7420 | 0.746 |
| Saint Lucia | Caribbean | Latin America and Caribbean | 0.4830 | 0.4930 | 0.5040 | 0.5140 | 0.5240 | 0.5340 | 0.5430 | 0.5510 | 0.5600 | 0.5680 | 0.5750 | 0.5810 | 0.5870 | 0.5930 | 0.6000 | 0.6060 | 0.6120 | 0.6170 | 0.6230 | 0.6290 | 0.6340 | 0.6390 | 0.6430 | 0.6480 | 0.6520 | 0.6560 | 0.659 | 0.6630 | 0.6670 | 0.670 |
| Saint Vincent and the Grenadines | Caribbean | Latin America and Caribbean | 0.4620 | 0.4710 | 0.4790 | 0.4870 | 0.4950 | 0.5020 | 0.5090 | 0.5150 | 0.5220 | 0.5280 | 0.5340 | 0.5390 | 0.5450 | 0.5510 | 0.5570 | 0.5620 | 0.5680 | 0.5740 | 0.5800 | 0.5850 | 0.5890 | 0.5930 | 0.5980 | 0.6020 | 0.6060 | 0.6100 | 0.615 | 0.6190 | 0.6230 | 0.627 |
| Samoa | Oceania | Southeast Asia, east Asia, and Oceania | 0.5310 | 0.5350 | 0.5390 | 0.5430 | 0.5460 | 0.5500 | 0.5550 | 0.5590 | 0.5630 | 0.5670 | 0.5710 | 0.5760 | 0.5810 | 0.5860 | 0.5900 | 0.5950 | 0.5990 | 0.6030 | 0.6070 | 0.6100 | 0.6120 | 0.6150 | 0.6180 | 0.6200 | 0.6230 | 0.6260 | 0.629 | 0.6330 | 0.6370 | 0.641 |
| San Marino | Western Europe | High income | 0.8140 | 0.8170 | 0.8200 | 0.8240 | 0.8280 | 0.8320 | 0.8370 | 0.8410 | 0.8460 | 0.8500 | 0.8520 | 0.8540 | 0.8560 | 0.8590 | 0.8610 | 0.8620 | 0.8640 | 0.8660 | 0.8680 | 0.8700 | 0.8720 | 0.8740 | 0.8760 | 0.8770 | 0.8790 | 0.8810 | 0.882 | 0.8820 | 0.8830 | 0.884 |
| Sao Tome and Principe | Western Sub-Saharan Africa | Sub-Saharan Africa | 0.2990 | 0.3020 | 0.3060 | 0.3090 | 0.3130 | 0.3170 | 0.3220 | 0.3270 | 0.3320 | 0.3380 | 0.3440 | 0.3510 | 0.3580 | 0.3650 | 0.3730 | 0.3810 | 0.3900 | 0.3980 | 0.4070 | 0.4160 | 0.4240 | 0.4330 | 0.4430 | 0.4520 | 0.4610 | 0.4700 | 0.478 | 0.4870 | 0.4950 | 0.502 |
| Saudi Arabia | North Africa and Middle East | North Africa and Middle East | 0.4800 | 0.4910 | 0.5040 | 0.5160 | 0.5290 | 0.5410 | 0.5540 | 0.5660 | 0.5780 | 0.5900 | 0.6020 | 0.6140 | 0.6250 | 0.6370 | 0.6500 | 0.6640 | 0.6770 | 0.6900 | 0.7030 | 0.7150 | 0.7260 | 0.7380 | 0.7500 | 0.7600 | 0.7690 | 0.7780 | 0.786 | 0.7930 | 0.8000 | 0.805 |
| Senegal | Western Sub-Saharan Africa | Sub-Saharan Africa | 0.2270 | 0.2330 | 0.2390 | 0.2450 | 0.2510 | 0.2570 | 0.2620 | 0.2670 | 0.2720 | 0.2770 | 0.2820 | 0.2860 | 0.2900 | 0.2950 | 0.2990 | 0.3040 | 0.3080 | 0.3130 | 0.3180 | 0.3240 | 0.3300 | 0.3360 | 0.3420 | 0.3480 | 0.3540 | 0.3610 | 0.368 | 0.3750 | 0.3820 | 0.389 |
| Serbia | Central Europe | Central Europe, eastern Europe, and central Asia | 0.6260 | 0.6350 | 0.6390 | 0.6390 | 0.6400 | 0.6400 | 0.6440 | 0.6470 | 0.6510 | 0.6570 | 0.6610 | 0.6650 | 0.6700 | 0.6760 | 0.6850 | 0.6940 | 0.7020 | 0.7090 | 0.7160 | 0.7230 | 0.7290 | 0.7350 | 0.7390 | 0.7440 | 0.7480 | 0.7530 | 0.756 | 0.7600 | 0.7630 | 0.767 |
| Seychelles | Southeast Asia | Southeast Asia, east Asia, and Oceania | 0.5670 | 0.5760 | 0.5840 | 0.5920 | 0.6000 | 0.6070 | 0.6140 | 0.6210 | 0.6290 | 0.6360 | 0.6420 | 0.6470 | 0.6520 | 0.6560 | 0.6590 | 0.6620 | 0.6660 | 0.6700 | 0.6730 | 0.6760 | 0.6790 | 0.6830 | 0.6870 | 0.6910 | 0.6960 | 0.7020 | 0.707 | 0.7130 | 0.7190 | 0.724 |
| Sierra Leone | Western Sub-Saharan Africa | Sub-Saharan Africa | 0.2070 | 0.2090 | 0.2100 | 0.2120 | 0.2150 | 0.2180 | 0.2180 | 0.2180 | 0.2190 | 0.2180 | 0.2190 | 0.2210 | 0.2240 | 0.2290 | 0.2340 | 0.2390 | 0.2450 | 0.2520 | 0.2600 | 0.2670 | 0.2750 | 0.2830 | 0.2920 | 0.3040 | 0.3140 | 0.3210 | 0.328 | 0.3350 | 0.3420 | 0.347 |
| Singapore | High-income Asia Pacific | High income | 0.6880 | 0.6970 | 0.7050 | 0.7140 | 0.7230 | 0.7310 | 0.7400 | 0.7490 | 0.7560 | 0.7620 | 0.7690 | 0.7760 | 0.7830 | 0.7890 | 0.7940 | 0.8010 | 0.8080 | 0.8140 | 0.8220 | 0.8280 | 0.8350 | 0.8390 | 0.8430 | 0.8470 | 0.8500 | 0.8520 | 0.855 | 0.8580 | 0.8600 | 0.861 |
| Slovakia | Central Europe | Central Europe, eastern Europe, and central Asia | 0.6560 | 0.6620 | 0.6680 | 0.6790 | 0.6930 | 0.7020 | 0.7090 | 0.7160 | 0.7240 | 0.7310 | 0.7390 | 0.7460 | 0.7520 | 0.7560 | 0.7600 | 0.7660 | 0.7720 | 0.7770 | 0.7810 | 0.7840 | 0.7890 | 0.7940 | 0.7980 | 0.8010 | 0.8030 | 0.8030 | 0.804 | 0.8050 | 0.8080 | 0.812 |
| Slovenia | Central Europe | Central Europe, eastern Europe, and central Asia | 0.7260 | 0.7310 | 0.7360 | 0.7410 | 0.7460 | 0.7510 | 0.7560 | 0.7620 | 0.7680 | 0.7740 | 0.7800 | 0.7870 | 0.7930 | 0.7970 | 0.8020 | 0.8070 | 0.8110 | 0.8140 | 0.8180 | 0.8200 | 0.8220 | 0.8240 | 0.8250 | 0.8270 | 0.8290 | 0.8310 | 0.833 | 0.8350 | 0.8380 | 0.840 |
| Solomon Islands | Oceania | Southeast Asia, east Asia, and Oceania | 0.2790 | 0.2830 | 0.2880 | 0.2940 | 0.3000 | 0.3060 | 0.3120 | 0.3170 | 0.3210 | 0.3260 | 0.3280 | 0.3300 | 0.3310 | 0.3320 | 0.3330 | 0.3360 | 0.3390 | 0.3430 | 0.3480 | 0.3530 | 0.3580 | 0.3640 | 0.3710 | 0.3770 | 0.3820 | 0.3870 | 0.393 | 0.3980 | 0.4030 | 0.407 |
| Somalia | Eastern Sub-Saharan Africa | Sub-Saharan Africa | 0.0508 | 0.0514 | 0.0522 | 0.0530 | 0.0539 | 0.0547 | 0.0556 | 0.0565 | 0.0574 | 0.0584 | 0.0593 | 0.0603 | 0.0613 | 0.0622 | 0.0632 | 0.0641 | 0.0651 | 0.0661 | 0.0671 | 0.0682 | 0.0692 | 0.0703 | 0.0716 | 0.0728 | 0.0742 | 0.0756 | 0.077 | 0.0785 | 0.0799 | 0.081 |
| South Africa | Southern Sub-Saharan Africa | Sub-Saharan Africa | 0.5520 | 0.5560 | 0.5600 | 0.5640 | 0.5670 | 0.5720 | 0.5760 | 0.5800 | 0.5840 | 0.5880 | 0.5930 | 0.5970 | 0.6020 | 0.6070 | 0.6110 | 0.6160 | 0.6220 | 0.6270 | 0.6320 | 0.6370 | 0.6420 | 0.6470 | 0.6510 | 0.6560 | 0.6600 | 0.6640 | 0.668 | 0.6720 | 0.6760 | 0.678 |
| South Sudan | Eastern Sub-Saharan Africa | Sub-Saharan Africa | 0.2480 | 0.2510 | 0.2530 | 0.2560 | 0.2590 | 0.2610 | 0.2640 | 0.2670 | 0.2710 | 0.2740 | 0.2770 | 0.2810 | 0.2840 | 0.2880 | 0.2920 | 0.2960 | 0.3010 | 0.3050 | 0.3100 | 0.3150 | 0.3200 | 0.3250 | 0.3300 | 0.3340 | 0.3390 | 0.3420 | 0.345 | 0.3510 | 0.3580 | 0.363 |
| Spain | Western Europe | High income | 0.6470 | 0.6550 | 0.6620 | 0.6690 | 0.6750 | 0.6810 | 0.6860 | 0.6910 | 0.6960 | 0.7000 | 0.7050 | 0.7090 | 0.7130 | 0.7170 | 0.7210 | 0.7250 | 0.7280 | 0.7300 | 0.7340 | 0.7390 | 0.7430 | 0.7460 | 0.7490 | 0.7520 | 0.7540 | 0.7560 | 0.759 | 0.7610 | 0.7640 | 0.767 |
| Sri Lanka | Southeast Asia | Southeast Asia, east Asia, and Oceania | 0.5040 | 0.5110 | 0.5180 | 0.5250 | 0.5320 | 0.5390 | 0.5470 | 0.5540 | 0.5610 | 0.5670 | 0.5730 | 0.5780 | 0.5820 | 0.5870 | 0.5920 | 0.5970 | 0.6020 | 0.6090 | 0.6150 | 0.6210 | 0.6280 | 0.6360 | 0.6440 | 0.6510 | 0.6580 | 0.6660 | 0.672 | 0.6780 | 0.6840 | 0.690 |
| Sudan | North Africa and Middle East | North Africa and Middle East | 0.2270 | 0.2340 | 0.2410 | 0.2480 | 0.2560 | 0.2630 | 0.2710 | 0.2800 | 0.2890 | 0.2980 | 0.3080 | 0.3180 | 0.3270 | 0.3370 | 0.3480 | 0.3580 | 0.3700 | 0.3810 | 0.3930 | 0.4040 | 0.4160 | 0.4280 | 0.4400 | 0.4510 | 0.4620 | 0.4740 | 0.485 | 0.4970 | 0.5070 | 0.515 |
| Suriname | Caribbean | Latin America and Caribbean | 0.4980 | 0.5030 | 0.5070 | 0.5110 | 0.5130 | 0.5150 | 0.5180 | 0.5230 | 0.5280 | 0.5330 | 0.5380 | 0.5450 | 0.5520 | 0.5600 | 0.5680 | 0.5740 | 0.5790 | 0.5840 | 0.5880 | 0.5930 | 0.5980 | 0.6020 | 0.6070 | 0.6110 | 0.6160 | 0.6200 | 0.625 | 0.6290 | 0.6330 | 0.636 |
| Sweden | Western Europe | High income | 0.7690 | 0.7750 | 0.7820 | 0.7880 | 0.7940 | 0.8010 | 0.8060 | 0.8110 | 0.8150 | 0.8190 | 0.8230 | 0.8260 | 0.8290 | 0.8320 | 0.8350 | 0.8380 | 0.8400 | 0.8420 | 0.8440 | 0.8460 | 0.8490 | 0.8520 | 0.8550 | 0.8580 | 0.8600 | 0.8630 | 0.865 | 0.8680 | 0.8700 | 0.872 |
| Switzerland | Western Europe | High income | 0.8680 | 0.8690 | 0.8730 | 0.8760 | 0.8780 | 0.8800 | 0.8810 | 0.8830 | 0.8840 | 0.8860 | 0.8890 | 0.8930 | 0.8940 | 0.8960 | 0.8980 | 0.9000 | 0.9020 | 0.9050 | 0.9080 | 0.9090 | 0.9120 | 0.9140 | 0.9170 | 0.9190 | 0.9210 | 0.9220 | 0.924 | 0.9260 | 0.9280 | 0.929 |
| Syrian Arab Republic | North Africa and Middle East | North Africa and Middle East | 0.3670 | 0.3760 | 0.3870 | 0.3980 | 0.4090 | 0.4210 | 0.4320 | 0.4430 | 0.4540 | 0.4650 | 0.4760 | 0.4860 | 0.4970 | 0.5080 | 0.5210 | 0.5350 | 0.5480 | 0.5600 | 0.5720 | 0.5830 | 0.5940 | 0.6010 | 0.6050 | 0.6060 | 0.6070 | 0.6080 | 0.610 | 0.6130 | 0.6160 | 0.619 |
| Taiwan (Province of China) | East Asia | Southeast Asia, east Asia, and Oceania | 0.6670 | 0.6780 | 0.6850 | 0.6940 | 0.7020 | 0.7110 | 0.7190 | 0.7310 | 0.7430 | 0.7470 | 0.7540 | 0.7630 | 0.7720 | 0.7790 | 0.7870 | 0.7950 | 0.8020 | 0.8100 | 0.8170 | 0.8240 | 0.8300 | 0.8330 | 0.8380 | 0.8430 | 0.8480 | 0.8520 | 0.856 | 0.8600 | 0.8650 | 0.868 |
| Tajikistan | Central Asia | Central Europe, eastern Europe, and central Asia | 0.4680 | 0.4730 | 0.4740 | 0.4740 | 0.4720 | 0.4680 | 0.4620 | 0.4570 | 0.4510 | 0.4450 | 0.4410 | 0.4400 | 0.4430 | 0.4480 | 0.4560 | 0.4630 | 0.4700 | 0.4770 | 0.4830 | 0.4890 | 0.4950 | 0.5000 | 0.5050 | 0.5110 | 0.5160 | 0.5210 | 0.526 | 0.5310 | 0.5350 | 0.539 |
| Thailand | Southeast Asia | Southeast Asia, east Asia, and Oceania | 0.5080 | 0.5180 | 0.5270 | 0.5360 | 0.5440 | 0.5530 | 0.5620 | 0.5690 | 0.5740 | 0.5780 | 0.5830 | 0.5890 | 0.5940 | 0.5990 | 0.6050 | 0.6100 | 0.6160 | 0.6230 | 0.6290 | 0.6330 | 0.6380 | 0.6430 | 0.6490 | 0.6550 | 0.6600 | 0.6660 | 0.671 | 0.6760 | 0.6820 | 0.687 |
| Timor-Leste | Southeast Asia | Southeast Asia, east Asia, and Oceania | 0.2740 | 0.2800 | 0.2860 | 0.2930 | 0.3000 | 0.3080 | 0.3170 | 0.3260 | 0.3340 | 0.3390 | 0.3450 | 0.3520 | 0.3580 | 0.3640 | 0.3740 | 0.3880 | 0.4060 | 0.4210 | 0.4360 | 0.4480 | 0.4580 | 0.4690 | 0.4800 | 0.4880 | 0.4930 | 0.4980 | 0.503 | 0.5080 | 0.5110 | 0.514 |
| Togo | Western Sub-Saharan Africa | Sub-Saharan Africa | 0.2660 | 0.2720 | 0.2780 | 0.2810 | 0.2860 | 0.2910 | 0.2960 | 0.3020 | 0.3060 | 0.3100 | 0.3130 | 0.3170 | 0.3200 | 0.3230 | 0.3270 | 0.3300 | 0.3340 | 0.3380 | 0.3420 | 0.3470 | 0.3520 | 0.3580 | 0.3640 | 0.3710 | 0.3790 | 0.3860 | 0.394 | 0.4020 | 0.4110 | 0.417 |
| Tokelau | Oceania | Southeast Asia, east Asia, and Oceania | 0.4270 | 0.4320 | 0.4380 | 0.4440 | 0.4500 | 0.4560 | 0.4630 | 0.4700 | 0.4770 | 0.4840 | 0.4910 | 0.4980 | 0.5040 | 0.5110 | 0.5190 | 0.5260 | 0.5340 | 0.5420 | 0.5500 | 0.5570 | 0.5650 | 0.5730 | 0.5800 | 0.5880 | 0.5950 | 0.6020 | 0.608 | 0.6150 | 0.6210 | 0.626 |
| Tonga | Oceania | Southeast Asia, east Asia, and Oceania | 0.5100 | 0.5170 | 0.5220 | 0.5270 | 0.5330 | 0.5380 | 0.5430 | 0.5470 | 0.5510 | 0.5550 | 0.5590 | 0.5630 | 0.5680 | 0.5720 | 0.5760 | 0.5800 | 0.5830 | 0.5870 | 0.5900 | 0.5940 | 0.5980 | 0.6020 | 0.6060 | 0.6100 | 0.6140 | 0.6180 | 0.622 | 0.6270 | 0.6320 | 0.636 |
| Trinidad and Tobago | Caribbean | Latin America and Caribbean | 0.6180 | 0.6220 | 0.6260 | 0.6300 | 0.6340 | 0.6390 | 0.6440 | 0.6500 | 0.6560 | 0.6620 | 0.6690 | 0.6750 | 0.6820 | 0.6890 | 0.6960 | 0.7040 | 0.7110 | 0.7180 | 0.7240 | 0.7280 | 0.7320 | 0.7360 | 0.7390 | 0.7420 | 0.7450 | 0.7480 | 0.751 | 0.7530 | 0.7550 | 0.757 |
| Tunisia | North Africa and Middle East | North Africa and Middle East | 0.4340 | 0.4440 | 0.4550 | 0.4660 | 0.4760 | 0.4870 | 0.4980 | 0.5080 | 0.5180 | 0.5280 | 0.5380 | 0.5480 | 0.5560 | 0.5650 | 0.5740 | 0.5820 | 0.5910 | 0.5990 | 0.6070 | 0.6140 | 0.6220 | 0.6280 | 0.6340 | 0.6400 | 0.6460 | 0.6510 | 0.657 | 0.6620 | 0.6670 | 0.672 |
| Turkey | North Africa and Middle East | North Africa and Middle East | 0.4730 | 0.4830 | 0.4930 | 0.5040 | 0.5130 | 0.5230 | 0.5340 | 0.5450 | 0.5560 | 0.5660 | 0.5770 | 0.5870 | 0.5970 | 0.6070 | 0.6190 | 0.6300 | 0.6410 | 0.6520 | 0.6630 | 0.6710 | 0.6800 | 0.6890 | 0.6980 | 0.7070 | 0.7150 | 0.7230 | 0.729 | 0.7360 | 0.7430 | 0.748 |
| Turkmenistan | Central Asia | Central Europe, eastern Europe, and central Asia | 0.5480 | 0.5510 | 0.5540 | 0.5570 | 0.5570 | 0.5580 | 0.5580 | 0.5570 | 0.5560 | 0.5570 | 0.5610 | 0.5650 | 0.5700 | 0.5760 | 0.5820 | 0.5880 | 0.5950 | 0.6010 | 0.6060 | 0.6110 | 0.6160 | 0.6220 | 0.6280 | 0.6350 | 0.6420 | 0.6480 | 0.654 | 0.6600 | 0.6660 | 0.670 |
| Tuvalu | Oceania | Southeast Asia, east Asia, and Oceania | 0.4260 | 0.4340 | 0.4420 | 0.4500 | 0.4580 | 0.4650 | 0.4700 | 0.4760 | 0.4830 | 0.4900 | 0.4960 | 0.5020 | 0.5090 | 0.5140 | 0.5190 | 0.5230 | 0.5270 | 0.5310 | 0.5370 | 0.5410 | 0.5450 | 0.5490 | 0.5530 | 0.5580 | 0.5620 | 0.5670 | 0.573 | 0.5790 | 0.5840 | 0.589 |
| Uganda | Eastern Sub-Saharan Africa | Sub-Saharan Africa | 0.1670 | 0.1690 | 0.1720 | 0.1760 | 0.1810 | 0.1870 | 0.1940 | 0.2010 | 0.2080 | 0.2160 | 0.2250 | 0.2340 | 0.2430 | 0.2530 | 0.2620 | 0.2720 | 0.2820 | 0.2930 | 0.3030 | 0.3140 | 0.3250 | 0.3350 | 0.3450 | 0.3550 | 0.3640 | 0.3730 | 0.382 | 0.3910 | 0.3990 | 0.404 |
| Ukraine | Eastern Europe | Central Europe, eastern Europe, and central Asia | 0.6530 | 0.6570 | 0.6610 | 0.6650 | 0.6660 | 0.6670 | 0.6670 | 0.6670 | 0.6660 | 0.6650 | 0.6640 | 0.6650 | 0.6680 | 0.6720 | 0.6790 | 0.6850 | 0.6920 | 0.6990 | 0.7060 | 0.7100 | 0.7130 | 0.7180 | 0.7210 | 0.7250 | 0.7270 | 0.7290 | 0.730 | 0.7320 | 0.7340 | 0.736 |
| United Arab Emirates | North Africa and Middle East | North Africa and Middle East | 0.6210 | 0.6370 | 0.6530 | 0.6690 | 0.6850 | 0.7000 | 0.7140 | 0.7270 | 0.7400 | 0.7510 | 0.7620 | 0.7730 | 0.7830 | 0.7920 | 0.8010 | 0.8100 | 0.8190 | 0.8290 | 0.8380 | 0.8460 | 0.8530 | 0.8590 | 0.8630 | 0.8680 | 0.8700 | 0.8720 | 0.874 | 0.8760 | 0.8790 | 0.880 |
| United Kingdom | Western Europe | High income | 0.7450 | 0.7490 | 0.7550 | 0.7610 | 0.7660 | 0.7690 | 0.7710 | 0.7740 | 0.7780 | 0.7840 | 0.7890 | 0.7930 | 0.7970 | 0.7990 | 0.8020 | 0.8040 | 0.8060 | 0.8080 | 0.8110 | 0.8130 | 0.8160 | 0.8200 | 0.8250 | 0.8300 | 0.8340 | 0.8370 | 0.839 | 0.8420 | 0.8450 | 0.847 |
| United Republic of Tanzania | Eastern Sub-Saharan Africa | Sub-Saharan Africa | 0.2600 | 0.2650 | 0.2690 | 0.2730 | 0.2760 | 0.2800 | 0.2840 | 0.2880 | 0.2920 | 0.2960 | 0.3010 | 0.3050 | 0.3100 | 0.3150 | 0.3200 | 0.3260 | 0.3310 | 0.3370 | 0.3420 | 0.3480 | 0.3540 | 0.3610 | 0.3680 | 0.3750 | 0.3830 | 0.3910 | 0.399 | 0.4080 | 0.4160 | 0.423 |
| United States of America | High-income North America | High income | 0.7680 | 0.7710 | 0.7750 | 0.7780 | 0.7820 | 0.7850 | 0.7880 | 0.7890 | 0.7910 | 0.7940 | 0.7970 | 0.8020 | 0.8060 | 0.8090 | 0.8110 | 0.8120 | 0.8110 | 0.8140 | 0.8190 | 0.8260 | 0.8320 | 0.8350 | 0.8390 | 0.8420 | 0.8450 | 0.8490 | 0.853 | 0.8560 | 0.8580 | 0.859 |
| United States Virgin Islands | Caribbean | Latin America and Caribbean | 0.6670 | 0.6800 | 0.6890 | 0.6970 | 0.7040 | 0.7100 | 0.7150 | 0.7200 | 0.7240 | 0.7280 | 0.7310 | 0.7340 | 0.7420 | 0.7490 | 0.7560 | 0.7620 | 0.7680 | 0.7730 | 0.7780 | 0.7820 | 0.7850 | 0.7880 | 0.7900 | 0.7910 | 0.7920 | 0.7940 | 0.795 | 0.7960 | 0.7980 | 0.799 |
| Uruguay | Southern Latin America | Latin America and Caribbean | 0.5810 | 0.5840 | 0.5880 | 0.5910 | 0.5940 | 0.5970 | 0.6000 | 0.6060 | 0.6120 | 0.6180 | 0.6220 | 0.6260 | 0.6280 | 0.6310 | 0.6330 | 0.6360 | 0.6390 | 0.6420 | 0.6450 | 0.6490 | 0.6530 | 0.6580 | 0.6630 | 0.6680 | 0.6730 | 0.6780 | 0.684 | 0.6880 | 0.6930 | 0.697 |
| Uzbekistan | Central Asia | Central Europe, eastern Europe, and central Asia | 0.4900 | 0.4920 | 0.4940 | 0.4960 | 0.4980 | 0.5010 | 0.5050 | 0.5100 | 0.5150 | 0.5200 | 0.5250 | 0.5310 | 0.5360 | 0.5410 | 0.5460 | 0.5510 | 0.5560 | 0.5610 | 0.5670 | 0.5720 | 0.5780 | 0.5840 | 0.5900 | 0.5970 | 0.6030 | 0.6090 | 0.616 | 0.6220 | 0.6270 | 0.631 |
| Vanuatu | Oceania | Southeast Asia, east Asia, and Oceania | 0.3610 | 0.3650 | 0.3690 | 0.3730 | 0.3770 | 0.3810 | 0.3860 | 0.3900 | 0.3950 | 0.3990 | 0.4030 | 0.4070 | 0.4100 | 0.4130 | 0.4170 | 0.4200 | 0.4250 | 0.4300 | 0.4350 | 0.4400 | 0.4460 | 0.4510 | 0.4550 | 0.4600 | 0.4640 | 0.4680 | 0.473 | 0.4770 | 0.4810 | 0.485 |
| Venezuela (Bolivarian Republic of) | Central Latin America | Latin America and Caribbean | 0.5090 | 0.5140 | 0.5220 | 0.5300 | 0.5370 | 0.5420 | 0.5460 | 0.5510 | 0.5540 | 0.5570 | 0.5590 | 0.5600 | 0.5580 | 0.5520 | 0.5480 | 0.5500 | 0.5570 | 0.5660 | 0.5750 | 0.5820 | 0.5860 | 0.5910 | 0.5960 | 0.6000 | 0.6040 | 0.6070 | 0.608 | 0.6080 | 0.6080 | 0.607 |
| Viet Nam | Southeast Asia | Southeast Asia, east Asia, and Oceania | 0.3900 | 0.3970 | 0.4040 | 0.4120 | 0.4200 | 0.4290 | 0.4380 | 0.4470 | 0.4550 | 0.4630 | 0.4710 | 0.4780 | 0.4860 | 0.4930 | 0.5010 | 0.5090 | 0.5170 | 0.5250 | 0.5330 | 0.5410 | 0.5490 | 0.5580 | 0.5660 | 0.5730 | 0.5810 | 0.5890 | 0.596 | 0.6040 | 0.6110 | 0.617 |
| Yemen | North Africa and Middle East | North Africa and Middle East | 0.1760 | 0.1830 | 0.1910 | 0.1980 | 0.2070 | 0.2150 | 0.2240 | 0.2340 | 0.2430 | 0.2530 | 0.2630 | 0.2730 | 0.2830 | 0.2930 | 0.3030 | 0.3140 | 0.3250 | 0.3350 | 0.3460 | 0.3560 | 0.3660 | 0.3750 | 0.3840 | 0.3930 | 0.4020 | 0.4070 | 0.410 | 0.4120 | 0.4130 | 0.412 |
| Zambia | Eastern Sub-Saharan Africa | Sub-Saharan Africa | 0.2990 | 0.3040 | 0.3070 | 0.3110 | 0.3140 | 0.3160 | 0.3190 | 0.3230 | 0.3260 | 0.3300 | 0.3340 | 0.3400 | 0.3450 | 0.3520 | 0.3590 | 0.3670 | 0.3760 | 0.3860 | 0.3960 | 0.4070 | 0.4180 | 0.4290 | 0.4400 | 0.4510 | 0.4620 | 0.4720 | 0.481 | 0.4910 | 0.4990 | 0.505 |
| Zimbabwe | Southern Sub-Saharan Africa | Sub-Saharan Africa | 0.3940 | 0.4030 | 0.4090 | 0.4150 | 0.4210 | 0.4260 | 0.4320 | 0.4370 | 0.4410 | 0.4440 | 0.4460 | 0.4480 | 0.4480 | 0.4470 | 0.4440 | 0.4410 | 0.4370 | 0.4330 | 0.4280 | 0.4250 | 0.4250 | 0.4270 | 0.4320 | 0.4380 | 0.4450 | 0.4520 | 0.459 | 0.4650 | 0.4710 | 0.476 |
